# Molecular Mingling: Multimodal Predictions of Ligand Promiscuity in Pentameric Ligand-Gated Ion Channels

**DOI:** 10.3389/fmolb.2022.860246

**Published:** 2022-05-09

**Authors:** Filip Koniuszewski, Florian D. Vogel, Konstantina Bampali, Jure Fabjan, Thomas Seidel, Petra Scholze, Philip B. Schmiedhofer, Thierry Langer, Margot Ernst

**Affiliations:** ^1^ Department of Pathobiology of the Nervous System, Center for Brain Research, Medical University Vienna, Vienna, Austria; ^2^ Department of Pharmaceutical Sciences, Division of Pharmaceutical Chemistry, University of Vienna, Vienna, Austria

**Keywords:** pentameric ligand-gated ion channels, cys-loop receptors, allosteric ligands, protein-ligand interactions, neuropsychiatric adverse events

## Abstract

**Background:** Human pentameric ligand-gated ion channels (pLGICs) comprise nicotinic acetylcholine receptors (nAChRs), 5-hydroxytryptamine type 3 receptors (5-HT_3_Rs), zinc-activated channels (ZAC), γ-aminobutyric acid type A receptors (GABA_A_Rs) and glycine receptors (GlyRs). They are recognized therapeutic targets of some of the most prescribed drugs like general anesthetics, anxiolytics, smoking cessation aids, antiemetics and many more. Currently, approximately 100 experimental structures of pLGICs with ligands bound exist in the protein data bank (PDB). These atomic-level 3D structures enable the generation of a comprehensive binding site inventory for the superfamily and the *in silico* prediction of binding site properties.

**Methods:** A panel of high throughput *in silico* methods including pharmacophore screening, conformation analysis and descriptor calculation was applied to a selection of allosteric binding sites for which *in vitro* screens are lacking. Variant abundance near binding site forming regions and computational docking complement the approach.

**Results:** The structural data reflects known and novel binding sites, some of which may be unique to individual receptors, while others are broadly conserved. The membrane spanning domain, comprising four highly conserved segments, contains ligand interaction sites for which *in vitro* assays suitable for high throughput screenings are critically lacking. This is also the case for structurally more variable novel sites in the extracellular domain. Our computational results suggest that the phytocannabinoid Δ^9^-tetrahydrocannabinol (Δ^9^-THC) can utilize multiple pockets which are likely to exist on most superfamily members.

**Conclusion:** With this study, we explore the potential for polypharmacology among pLGICs. Our data suggest that ligands can display two forms of promiscuity to an extent greater than what has been realized: 1) Ligands can interact with homologous sites in many members of the superfamily, which bears toxicological relevance. 2) Multiple pockets in distinct localizations of individual receptor subtypes share common ligands, which counteracts efforts to develop selective agents. Moreover, conformational states need to be considered for *in silico* drug screening, as certain binding sites display considerable flexibility. In total, this work contributes to a better understanding of polypharmacology across pLGICs and provides a basis for improved structure guided *in silico* drug development and drug derisking.

## Introduction

In mammalian organisms, the superfamily of pentameric ligand gated ion channels (pLGICs, also often termed cys-loop receptors) comprises ligand gated cation and anion channels, many of which are highly expressed in the mammalian nervous system. Several members of the superfamily are well established targets of important neuropsychiatric medications (Young and Snyder, 1973; Walstab et al., 2010; Dineley et al., 2015; Sieghart and Savic, 2018; Tregellas and Wylie, 2019; Breitinger and Breitinger, 2020; Caton et al., 2020). Beyond clinically approved drugs, several pLGICs are also targets of recreational and illicit drugs, and mediate toxicological effects by many synthetic, plant- and animal derived toxins (Ho and Flood, 2004; Johnston et al., 2006). Thus, all of the pLGIC genes and their resulting protein products which are found in nervous system tissues comprise a large group of potential off-targets that might mediate neuropsychiatric toxicological liabilities.

The EU funded NeuroDeRisk project (https://neuroderisk.eu/) is concerned with improving the preclinical prediction of neuropsychiatric adverse drug effects, which cause considerable problems in drug development. Its scope covers three types of drug induced adverse events (AEs): 1) seizures and convulsions; 2) psychological and psychiatric adverse events; and 3) peripheral neuropathies. The overarching project goal is to improve *in silico* and *in vitro* alerts for these three groups of nervous system AEs. Table 1 provides some examples of pLGIC mediated toxicities of interest for the NeuroDeRisk project.

**TABLE 1:** Examples of known neuropsychiatric adverse events from the NeuroDeRisk scope elicited by compounds which bind at the different pLGIC family members.

Protein family	Ligand	Adverse effects
GABA_A_Rs	Picrotoxin, bicuculline	Seizures
	Diazepam/benzodiazepines	Amnestic effects, unwanted over-sedation, addiction and many others
	Barbiturates, propofol	Addiction
GlyRs	Strychnine, tranexamic acid	Convulsions
nAChR	Nicotine	Addiction, insomnia, nausea/vomiting
	Varenicline	Psychiatric adverse events, seizures, headache, nausea
	Mecamylamine	Amnestic effect, negative influence on cognition and learning
5-HT_3_Rs	Antiemetics (setrons)	Headache, fatigue, malaise

**FIGURE 1:**
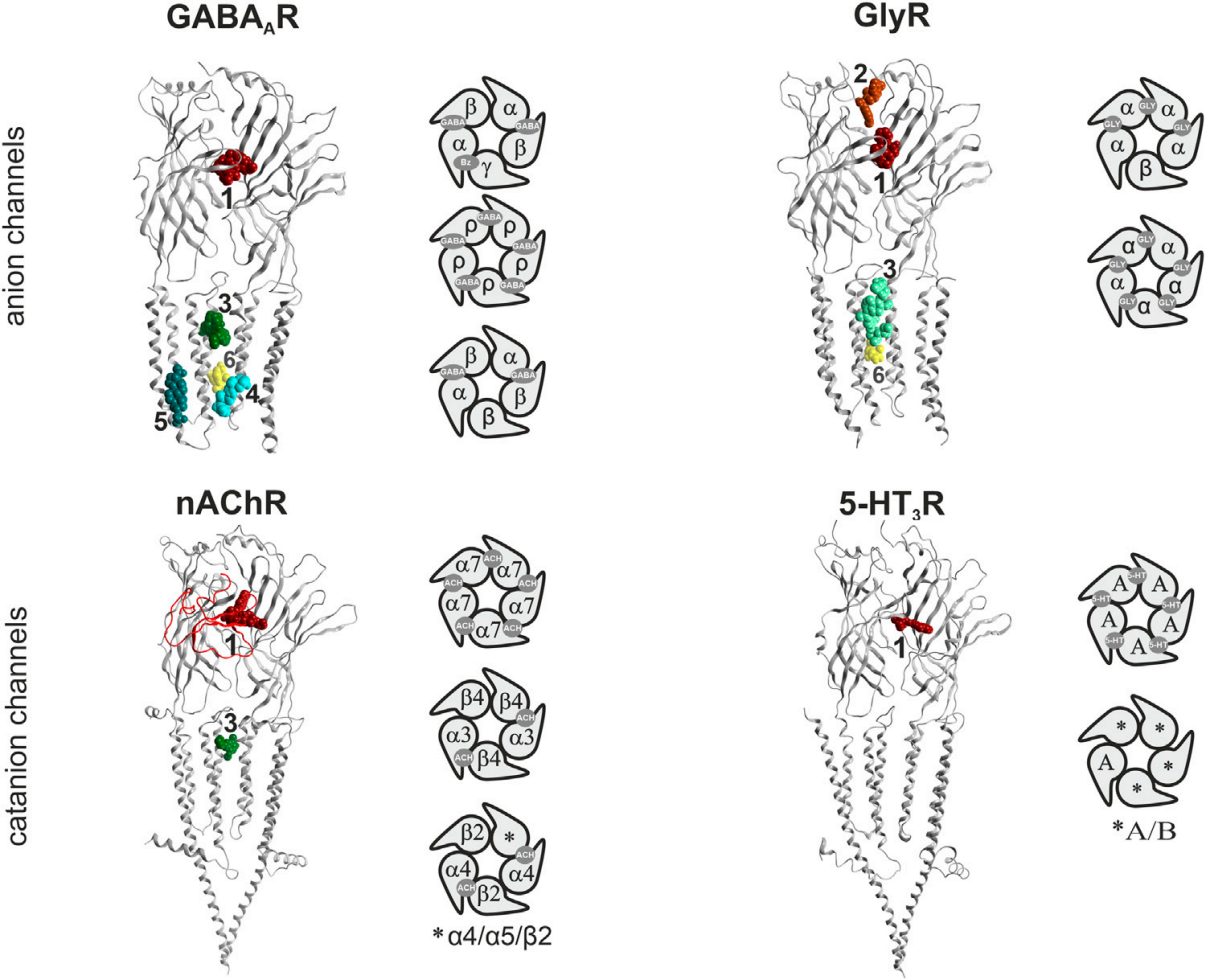
Binding sites in pLGICs. For each pLGIC family, one representative subunit dimer is displayed as ribbon structure, in superposition with selected ligand bound structures. The superposition of multiple ligands (where available) serves to give an impression of the overall volume of the pockets. The displayed structures are as follows: GABA_A_Rs: ribbon and picrotoxin (yellow, channel blocker site 6): 6X40; canonical ECD site 1 (red in all panels): superposition of diazepam/6HUP, bicuculline/6X3S, flumazenil/6X3U; upper TMD interface site 3 (green shades in all panels): etomidate/6X3V and propofol/6X3T; lower TMD interface site 4 (cyan): alphaxalone/6CDU; TM3/TM4- lipid associated site 5 (ocean blue): pregnenolone sulfate/5OSC. GlyR: ribbon and glycine in canonical ECD site: 5BKG; upper ECD interface site 2 (brown): AM-3607/5TIO; site 3: ivermectin/5VDI; channel blocker site 6: 6UD3. nAChR: ribbon with alpha-bungarotoxin at site 1 in red tube: 7KOO, site 1: varenicline/6UR8 and EVP-6124/7EKT, site 3: PNU-120596/7EKT. 5-HT_3_R: ribbon and site 3 granisetron/6NP0. Next to the ribbon renderings, some representative pentameric arrangements are shown schematically. For each family, the binding sites for the orthosteric agonists, and the high affinity benzodiazepine site (Bz) of GABA_A_ receptors are displayed in the selected pentameric arrangements.

The human pLGICs comprise the γ-aminobutyric acid- (GABA) and glycine- gated anion channels (GABA_A_Rs, GlyRs), and the cation permeable acetylcholine-, 5-HT- type 3, and zinc- activated channels (nAChRs, 5-HT_3_Rs, ZAC). As far as it is known, all vertebrate pLGICs have agonist binding sites in the extracellular domain at specific subunit interfaces. The interface forming subunit sides are traditionally called “principal” or (+), and “complementary” or (−). All pLGICs also feature a site for channel blocking ligands in the channel pore, and multiple allosteric binding sites in the extracellular and the transmembrane domains (Figure 1) (Thompson et al., 2010). Thus, drug effects can arise from multiple ligand interaction sites.

The GABA_A_ receptor (GABA_A_Rs) assemblies are drawn from a panel of 19 mammalian subunits (six α, three β, three γ, one δ, three ρ, one ε, one π and one θ) and their respective variants (splice isoforms, RNA-editing variants). Their ubiquitous presence in neuronal synaptic and extra-synaptic compartments (Olsen and Sieghart, 2008; Brickley and Mody, 2012) and in many glial cell types (such as astrocytes and microglia), along with a large number of identified and potential subunit assemblies, results in great pharmaco- toxicological complexity. Many subunit assemblies are thought to consist of two α, two β and one γ subunit (Sieghart, 2015). The canonical GABA binding site is located at the extracellular domain (ECD) β+/α− subunit interface of these receptors, while the homologous α+/γ− subunit interfaces form the high affinity benzodiazepine (Bz) binding sites (Sieghart, 2015). Many allosteric binding sites have been described and mediate the complex mode of action of GABA_A_R targeting compounds (Sieghart, 2015; Puthenkalam et al., 2016; Sieghart and Savic, 2018). Most drugs in current use, such as the well-known and widely used Bzs, are allosteric modulators of these receptors and as such alter GABA elicited channel activity (Rudolph and Knoflach, 2011; Sieghart and Savic, 2018). Broadly speaking, enhancing GABA effects leads to CNS depressant effects such as sedation, and to an increase of seizure threshold in anti-epileptic treatment, while reduction of GABA effects leads to CNS stimulatory effects such as arousal, anxiogenesis, and seizurogenesis. For example, the channel blocker picrotoxin induces seizures. This broad stroke picture needs to be interpreted cautiously, as evidenced by low dose paradoxical effects which are a common unwanted effect of many GABA_A_R targeting sedatives (Mancuso et al., 2004). Unwanted neuropsychiatric effects associated with GABA_A_Rs cover a very broad range and include seizures and convulsions, sedation, amnesia, addiction, mood changes such as the low dose aggression induction observed for Bzs, and many more (Bond, 1998; Paton, 2002; Mancuso et al., 2004; Albrecht et al., 2014). Despite many years of intense preclinical research and a large body of clinical studies, the exact molecular substrates of many unwanted effects mediated by human GABA_A_R populations remain unclear with the literature containing many controversies (Skolnick, 2012; Sieghart and Savic, 2018).

Glycine receptors (GlyRs) are a smaller family of pLG anion channels, featuring five human GlyR subunits (α1–4, β) (Lynch, 2004; Lynch et al., 2017). The α4 subunit encoding gene is a pseudogene in humans due to an early stop codon (Simon et al., 2004). Similar to GABA_A_ receptors, they chiefly mediate inhibitory effects in the CNS. Their spatial expression is more restricted compared to the almost omnipresent GABA_A_Rs. In the spinal cord and brain stem they are mainly located at the post-synapse, whereas in the brain pre-synaptic and extra-synaptic GlyRs are more abundant (Lynch, 2004). Despite the high number of GlyR modulators, so far no specific glycine receptor targeting therapeutic has emerged, in contrast to the widely targeted GABA_A_Rs. A pharmacological distinction between GABA_A_Rs and GlyRs can be made with the highly specific orthosteric antagonist strychnine, a plant derived toxin. Pathophysiologically, strychnine-induced convulsions occur at low doses due to the block of GlyR-mediated inhibition, whereas GABA_A_Rs are blocked at much higher concentrations (Breitinger and Breitinger, 2020). Additionally, tranexamic acid and ε-aminocaproic acid are two clinically used antifibrinolytics which can cause convulsions as a GlyR-mediated side effect (Lecker et al., 2012). The GlyRs high promiscuity within the pLGIC family is reflected by the number and diversity of compounds that bind to and modulate the receptors, such as taurine, beta-alanine, ivermectin, bicuculline, gabazine, as well as fluoxetine and clozapine (Breitinger and Breitinger, 2020). Many more clinically used therapeutics modulate GlyRs and might elicit some fraction of their adverse effects via unwanted modulation of these receptors, especially at higher dosages.

nAChRs are pLG cation channels that are found both in the central and peripheral nervous system including the neuromuscular junction. They are built out of a choice of 16 subunits (α1–7, α9–10, β1–4, γ, δ and ε) assembled around a central sodium- and calcium-selective pore. Muscle specific and neuron specific hetero-meric and homopentameric nAChRs have been described (Gotti et al., 2006; Gotti et al., 2007; Zoli et al., 2018). The endogenous ligand acetylcholine binds at the extracellular domain of the nAChR, at the interface between an α (+) and a β (−) subunit or two α7− subunits. Both α4β2− in the central nervous system, and α3β4− receptors in the peripheral nervous system can contain additional accessory subunits. These confer altered physiological, pharmacological and/or trafficking properties as has been shown for the α5-subunit (Scholze and Huck, 2020). As for GABA_A_Rs and GlyRs, allosteric modulatory sites have been described (Chatzidaki and Millar, 2015). Interestingly, allosteric ligands of nAChRs can mediate additional metabotropic-like signal transduction behavior (Horenstein and Papke, 2017). All nAChR targeting therapeutics in current use bind at agonist sites with different subtype preferences, and are chiefly used as smoking cessation aids. Varenicline and nicotine (applied orally as chewing gum, as nasal spray, or as transdermal patch), are approved by the U.S. Food And Drug Administration (FDA) and the European Medicines Agency (EMA), and proved to be effective medications to reduce smoking (Rosen et al., 2018). Nicotine patches have been described to cause side-effects such as addiction, insomnia, nausea and vomiting (Tønnesen et al., 1999; Schnoll et al., 2010). Although no direct effects on peripheral neuropathy is known to date, nicotine also worsens nerve regeneration after injury due to cytokine interactions (Rodriguez-Fontan et al., 2020)*.* Initially varenicline was suspected to trigger epileptic seizures in rats as well as in humans (Erken et al., 2014), but a recent case-control study showed no significant association (Chopra et al., 2019). Neuropsychiatric side effects (depression, anxiety, panic attacks, etc.) are however still discussed (Menkü et al., 2021). Mecamylamine is a nAChR competitive antagonist. It was initially developed as a ganglion-blocker that inhibits the action of the sympathetic nervous system, and has antihypertensive properties. It has been used for this purpose for several decades before finally being replaced (Young et al., 2001).

5-HT_3_Rs, similar to nAChRs, are cation selective and the only ion channels among serotonin (5-HT) receptors. Five 5-HT_3_R subunits are known (namely A, B, C, D and E) with various post-translational modifications and splice variants (Brüss et al., 2000; Tzvetkov et al., 2007; Walstab et al., 2010). Only the 5-HT_3_A subunit is able to form functional homopentamers. Furthermore, its presence in a heteropentamer seems to be necessary since it has been shown to play a key role in assembly and trafficking (Niesler et al., 2007; Holbrook et al., 2009) [heterogeneity of native receptors reviewed in (Jensen et al., 2008)]. The majority of 5-HT_3_Rs in the brain are located presynaptically as evidenced in rodent studies, with the exception of the hippocampus where they are localized mainly postsynaptically in somatodendritic regions (Miquel et al., 2002). Most ligands interact with 5-HT_3_Rs through the orthosteric site (ECD interface between two subunits) and the channel site (Thompson and Lummis, 2013) [molecular determinants of ligand binding reviewed also in (Thompson et al., 2010)]. An allosteric intersubunit site has also been described in the transmembrane domain (TMD) (Trattnig et al., 2012). Currently, the main therapeutic application of drugs, specifically antagonists, targeting 5-HT_3_Rs is for the management of chemotherapy- or radiation-induced and post-operative nausea and vomiting. 5-HT_3_R antagonists used as antiemetics, also called setrons, include ondansetron, granisetron, dolasetron and palonosetron. They target 5-HT_3_Rs on the vagal afferent nerve in the gut and on the chemoreceptor trigger zone in the brainstem (Basak et al., 2020). Adverse effects are common among 5-HT_3_R antagonists and include mild to moderate headache which is the most frequently observed side effect, appearing in approximately 20%–30% of patients (Hainsworth, 2014). Other side effects include fatigue and malaise occurring in up to 13% of patients (Theriot et al., 2021).

Another, sometimes termed atypical, member of the cys-loop receptor superfamily is the zinc-activated channel (ZAC) (Davies et al., 2003). ZAC is directly gated by the ions Zn^2+^, Cu^2+^ and H^+^, a property that seems to be unique among mammalian cys-loop receptors (Trattnig et al., 2016; Madjroh et al., 2021). Additionally, ZAC has distinct gating characteristics as seen with homomeric ZACs expressed in heterologous systems, such as considerable spontaneous activity, and slow activation and desensitization kinetics (Madjroh et al., 2021). However, little is known about the physiological role of this receptor. It has low amino acid sequence homology with the other members of the superfamily, but contains most of the major structural elements of a cys-loop receptor subunit (Madjroh et al., 2021). ZAC is expressed in several organs, such as pancreas, placenta, prostate, thyroid, as well as the adult and fetal brain (Davies et al., 2003; Houtani et al., 2005). The exploration of pharmacological tools to specifically target ZAC has proven to be challenging due to off-target action of several allosteric modulators in other cys-loop receptors (Madjroh et al., 2021), thus it is likely also an unknown player in adverse events elicited by pLGIC targeting substances.

In total, 45 human cys-loop receptor subunits contribute to a wide range of toxicological liabilities, which are only in part understood as the physiological function of many family members is still unknown. In the spectrum of unwanted effects, seizures and convulsions, adverse psychological and psychiatric effects, and some indirect impact on processes involved in peripheral neuropathies, place them in the center of NeuroDeRisk’s attention. In this study we thus examine all binding sites present in vertebrate pLGICs with structural evidence for their existence. Moreover, we examine a panel of *in silico* tools which facilitate prediction of off-target effects coming from binding sites that are highly conserved across the different family members. The complete list of structures that was integrated into our study comprises 31 GABA_A_Rs, 41 GlyRs, 26 nAChRs and 16 5-HT_3_Rs structures (Supplementary Item S1). These structures cover a total of 20 unique subunits, or fragments of subunits.

All cys-loop receptor subunits are glycoproteins with three domains (Figure 1). The ECDs and TMDs are highly conserved across the entire superfamily, while the intracellular domains (ICDs) are much more variable. Atom-level structural knowledge provides most data for the TMDs and partial or complete ECDs of multiple superfamily members. So far, fragments of ICDs have been structurally resolved only for nAChRs and 5-HT_3_Rs (Polovinkin et al., 2018; Gharpure et al., 2019).

The available structures comprise mainly homopentameric and a few heteropentameric GABA_A_R and nAChR assemblies. For all families which contain heteropentameric assemblies, the existing subunit stoichiometries and arrangements are still under investigation and in part controversially debated in the literature (Thompson et al., 2010) (see schematic pentamers in Figure 1). For heteromeric GABA_A_Rs, the subunit arrangement for α1βγ2 assemblies is known (Figure 1). Binary α1β3 assemblies feature 2α:3β stoichiometry (Kasaragod et al., 2022), containing two GABA sites and a histamine site. Information for arrangements with other subunits is lacking or controversially debated at the time of writing. Concerning GlyRs, the arrangement and stoichiometry of heteromeric receptors has been extensively debated, however recent studies show an arrangement of 4α:1β, as depicted in Figure 1 (Yu et al., 2021; Zhu and Gouaux, 2021). In neurons, heteromeric nAChRs consisting of combinations of α2, 3, 4, or 6 with β2 or 4 and homomeric α7 receptors are most dominant (Gotti et al., 2006; Gotti et al., 2007; Zoli et al., 2018). The arrangement of the muscle type receptor is also known (Zuber and Unwin, 2013), and arrangements with and without the accessory α5 subunit have been described (Scholze and Huck, 2020). Conflicting evidence regarding the exact arrangement of subunits in 5-HT_3_AB receptors exists. Barrera et al. showed using atomic force microscopy a subunit stoichiometry of 2A:3B and a proposed arrangement of B-A-B-B-A (Barrera et al., 2005). This is in contrast to other studies suggesting an A-A interface being present in 5-HT_3_AB receptors (Brady et al., 2001; Lochner and Lummis, 2010). However, it is acknowledged that the incorporation of the 5-HT_3_A subunit is necessary in order to form functional heteromeric receptors (Figure 1) (Jensen et al., 2008).

For homo- and heteropentameric mammalian pLGICs or closely homologous vertebrate family members, ligand bound pockets with a broad diversity of ligands are by now available in the PDB (Supplementary Figure S1). Multiple structures of ECD ligand bound family members are available with several ligands for all families with the exception of the ZAC (Figure 1; Supplementary Item S1). The known sites at the ECD subunit interface form either the orthosteric agonist/inverse agonist sites, or allosteric modulatory sites, depending on the subunits which contribute to the interface. At the ECD interface, a novel pocket has been observed in GlyRs (Figure 1) (Huang et al., 2017). Ligated pockets have been reported at the upper TMD interface for nAChRs, GABA_A_Rs and GlyRs, while the lower TMD modulatory steroid site and a lipid associated steroid bound site (Laverty et al., 2017; Miller et al., 2017) have so far only been studied in GABA_A_Rs (Figure 1).

Additional candidate binding sites have been observed in more remote members of the pLGIC family that belong to evolutionary distant organisms and share in part low sequence identity. Their 3D architecture reveals that the overall structural features are conserved from bacteria to complex eukaryotes (Supplementary Figure S1) (Salari et al., 2014). Moreover, cholesterol and derivatives thereof have been identified in a number of structures (Laverty et al., 2017; Walsh et al., 2018; Zhao et al., 2021; Zhu and Gouaux, 2021). Recently, mutational evidence for a novel, intrasubunit site in the ECD of GABA_A_R α5 subunits was presented (Bampali et al., 2022).

While it is well established that the pLGICs can mediate a wide range of neuropsychiatric AEs including seizures, alterations in various cognitive and mood functions (e.g., sedation, amnesia, anxiogenesis, paradoxical excitation and aggression, addiction and many more), the currently available *in vitro* high throughput screens still miss interactions of small molecules with pLGICs. This is partly due to the fact that standard screening assays cover only a fraction of the existing binding sites (Chatzidaki and Millar, 2015; Puthenkalam et al., 2016). Moreover, in the case of many compounds, functional studies show they bind at, and interact with specific pLGICs, but fail to identify the binding sites through which the effects are elicited. The binding sites displayed in Figure 1 form the basis for the multimodal *in silico* prediction of polypharmacology alerts we undertook with the aim to generate testable predictions for binding sites lacking radioligands.

## Methods

### Protein Sequences and Structures

All human canonical sequences have been obtained from UniProt. The full list of UniProt IDs and the corresponding gene names are listed in the Supplementary Table S1.

### Variant Data Mining

Data for the genes was downloaded from GnomAD versions 3.1.1 and 2.1.1. We used the former for the analysis and the latter portion of the data for cases of variants for which we could not find an amino acid match in the reference sequences. The data was fetched manually and then analyzed using Python 3 (Van Rossum and Drake Jr, 2009) and Pandas (McKinney, 2010; team, T.p.d., 2020). As the variants have a wide spread of screened alleles we filtered the outliers (Supplementary Figure S2). Next, we filtered out the variants, which have the frequency lower than 0.001%. Furthermore, we retained only missense variants. Those we transferred onto a reference numbering based on an alignment of all reference protein sequences.

### PDB Files

All experimental structures analyzed in this study have been taken from the PDB (Supplementary Item S1) (Unwin and Fujiyoshi, 2012; Miller et al., 2017; Basak et al., 2018a; Basak et al., 2018b; Chen et al., 2018; Gharpure et al., 2019; Noviello et al., 2021; Zhang et al., 2021; Zhu and Gouaux, 2021).

### Drug Dataset Preparation and Pharmacophore Screening Database Generation

The molecular structures of the majority of the 168 investigated drugs (Supplementary Item S2) were downloaded from DrugBank (https://go.drugbank.com/) (Wishart et al., 2018) (154 in total). 12 of the 168 drugs were not available on DrugBank and have been retrieved from PubChem (https://pubchem.ncbi.nlm.nih.gov/) (Kim et al., 2016) instead. The remaining two drugs—ivermectin and picrotoxin represent receptor-bound ligand structures and were extracted from the PDB complexes 5VDH and 6X40 (Berman et al., 2000), respectively. Structures of drugs taken from PubChem and DrugBank were all downloaded and stored as SD-files containing 2D atom coordinates. This format was chosen because several of the investigated drugs get administered as mixtures of stereoisomers and information about undefined stereocenters is not present in SD-files containing 3D atom coordinates. The individual drug SD-files were then concatenated to a single SD-file for further processing by the software Flipper (https://docs.eyesopen.com/applications/omega/flipper.html, version 3.1.1.2, with default settings for all non-mandatory options) in order to enumerate all possible stereoisomers of drugs having one or more undefined stereocenters. The resulting SD-file contained a total of 246 structures which were then subjected to molecule conformer ensemble generation using the program OMEGA (http://www.eyesopen.com/, version 3.1.1.2, with default settings for all non-mandatory options). For the generation of the pharmacophore screening database, a simple KNIME workflow (Berthold et al., 2008) using nodes from the LigandScout KNIME extension (Wolber and Langer, 2005) (version 1.8.0, http://www.inteligand.com/download/LigandScout_Knime_Nodes.pdf, http://www.inteligand.com/ligandscout3/) was created which consisted of a SDF-reader node connected to a LDB-writer node. In the SDF-reader node configuration dialog the multi-conformer SD-file generated by OMEGA was chosen as input file, molecule conformer detection got enabled and “Compare by Name” has been selected as associated conformer matching strategy. After execution of the workflow, a pharmacophore screening database with 167 records has been obtained. Ethanol was not included in the output database since its pharmacophores do not comprise at least three features with distinct spatial positions (a prerequisite for LigandScouts pharmacophore alignment algorithm). In the generated database, every entry corresponds to a particular drug of the dataset and comprises all of its stereoisomers and associated conformers. This means, a drug will be considered as a hit in the screening process if at least one of its stereoisomers matches the query pharmacophore. This mimics the situation in physiological systems when a drug gets administered as a mixture of stereoisomers and not all isomers of the drug are active towards the target receptor.

### Structure-Based Pharmacophore Generation

Pharmacophores of ligands bound to the assessed target binding sites were generated using the software LigandScout (http://www.inteligand.com/ligandscout3/, version 4.4.8) (Wolber and Langer, 2005) by employing the following general procedure: First, the PDB file providing the structure(s) of the receptor bound ligand was loaded into the “structure-based” perspective of LigandScout. Afterwards, for every binding site of interest (Supplementary Table S2), a structure-based pharmacophore of the ligand was generated (using default settings) by zooming into the corresponding site and initiating the pharmacophore generation process. The obtained structure-based pharmacophores were continuously collected in LigandScouts “alignment” perspective and, after all PDB files have been processed, stored in a single file for further processing.

### Generation of “Merged” Pharmacophores

Pharmacophores representing combinations of multiple structure-based pharmacophores were generated in the “alignment” perspective of LigandScout using the “merged” pharmacophore generation functionality. The pharmacophores “[3/Ach (α7+/α7−)_18 Hits]” (merged pharmacophores of PNU-120596 in PDB entry 7EKT), “[3/Gly (α3+/α3−)_119 Hits]” (merged pharmacophores of ivermectin in PDB entry 5VDH), “[3/GABA (α1+/β2−); (γ2+/β2−)_36 Hits]” (merged pharmacophores of phenobarbital in PDB entry 6X3W) and “[4/(GABA (α1+/α1−)_74 Hits” (merged pharmacophores of pregnanolone in PDB entry 5O8F, tetrahydrodeoxycorticosterone in PDB entry 5OSB and alphaxolone in PDB entry 6CDU) (Supplementary Table S2) were all obtained via a feature-based alignment strategy since the ligands in each of these complexes displayed identical binding modes. However, slight differences were observed for the structure-based pharmacophores of ivermectin in 5VDH. Two of the ivermectin features (14 in total) that were not present in all sites of 5VDH have therefore been marked as optional for matching in the performed pharmacophore screening runs.

For the generation of the pharmacophore models “[3/GABA (γ2+/β2−)_103 Hits]” (merged pharmacophores of diazepam in 6X3X [E] and phenobarbital in 6X3W [E]), “[3/GABA (β(2/3)+/α1−)_84 Hits_]” (merged pharmacophores of propofol in 6X3T [D], etomidate in 6X3V [A] and diazepam in 6HUP [E]) a reference point (Cɑs of proximal binding site residues) alignment strategy has been chosen. This was appropriate since the ligands do not share a common binding mode but nevertheless bind to overlapping regions of homologous receptor sites.

### Pharmacophore Screening

The parallel screening of the generated structure-based and merged pharmacophores against the prepared drug database was performed by means of KNIME workflows using the activity profiling node provided by the LigandScout KNIME extension (version 1.8.0). The corresponding inputs of the activity profiling node were connected to a PMZ-reader node providing the screened pharmacophore dataset and a LDB-reader node specifying the drug database LDB file. For screening with the structure-based pharmacophore dataset, the retrieval mode in the activity profiling node configuration has been set to “Get best matching conformation,” exclusion volume checks were enabled and the omitted feature count was set to zero. For screening with the merged pharmacophore dataset, the retrieval mode was set set to “Stop after first matching conformation,” exclusion volume checks were enabled and the omitted feature count has been set to 9 in order to require only 3 matching drug features for any of the query pharmacophores (even in the case of the largest pharmacophore “[3/Gly (α3+/α3−)_119 Hits]” with 12 mandatory features). After execution of the screening workflows, the obtained results were saved in CSV format via the heatmap view of the activity profiling node.

### Generation of Reference Alignment for Variant Analysis

Based on the data from GnomAD versions 3.1.1 and 2.1.1 and the canonical sequences, an alignment was generated: The alignment was produced using MOE (Inc, C.C.G, 2016) by first aligning AlphaFold-predicted structures (Tunyasuvunakool et al., 2021) for all the main isoforms. For the section of the sequences before strand 1 the published structures were used for reference. The intracellular part of the proteins between TM3 and TM4 was separately aligned for minimization of gaps. After finishing the alignment of the major isoforms, other isoforms were added to the alignment with the exception of the ones too short and diverging for a confident mapping to be possible. Alignment was subsequently used in the Python environment through Biopython (Cock et al., 2009). The alignment is available as Supplementary Item S3.

### Sequence and Sequence-To-Structure Alignments and Similarity Calculation

Sequence alignments have been produced with Clustal X (Larkin et al., 2007) and Promals3D (Pei et al., 2008). Sequence-to-structure alignments were produced with Promals3D, PDBeFold (Krissinel and Henrick, 2004) and MOE. Specific alignments and supporting information are provided as individual FASTA files (Supplementary Item S3) and Supplementary Figures S3, S10. Pairwise sequence similarity was calculated by using the sequence similarity monitor script from MOE (ULC, 2022) with PAM180 substitution matrix.

### Analysis of Binding Sites

Binding sites have been identified through the 3D structures of the pentameric ligand-gated ion channels that are found in the PDB. These structures with ligands in the binding sites of interest were further analyzed in MOE. The ligplot function and visual inspection of the binding sites were used to identify the pocket-forming amino acids for subsequent local sequence similarity calculations and the descriptor analysis. For the TMD, the chosen amino acids (colored in the sequence alignment in the Supplementary Item S4) were used for all subsequent procedures (similarity and descriptor calculation, mapping of amino acids and variants to a representative ribbon structure, computation of Cɑ distances for the conformational analysis).

### Pocket Properties, Descriptor Set Calculations

For the proteochemometric (PCM) modeling to model the ligand-target interaction space the z-Scales(3) (Sandberg et al., 1998) and the MSWIM (Zaliani and Gancia, 1999) descriptors have been calculated using the R package “peptides” (Osorio et al., 2015) for each binding site in RStudio 1.4.1717 (http://www.rstudio.com).

### Analysis and Visualization of Descriptors

The z-Scale(3) and MSWHIM have been plotted in a 3D scatter plot and all pairwise euclidean distances have been calculated by using the Plotly (Plotly Technologies Inc., 2015) and Numpy (Harris et al., 2020) libraries in Python 3.8. Through these distances, a dendrogram and a heatmap have been created with hierarchical clustering by using Seaborn (Waskom et al., 2021). The clusters have been visualized by using Plotly.

### Analysis and Visualization of the Variants

All the following visualizations were produced using Matplotlib (Hunter, 2007).

### Ribbon Heatmaps of Conserved Regions

For each position in the reference sequence the sum of minor allele count per 100,000 was calculated. The values were transferred to 7EKT [A] (Zhao et al., 2021). The conserved regions of the proteins were also mapped onto 7EKT [A] and for those positions the values were transformed into RGB values. The values for the rest of the sequence were set to yellow (0xb3b300). The colors were then transferred to MOE and applied to the 7EKT [A] structure.

### Heatmaps of Variable Regions

Variable regions were mapped on the reference alignment. For each gene and each region, we calculated the sum of minor allele count per 100,000, divided by the length of the region in the gene’s major isoform.

### Binding Site Heatmaps

The amino acids involved in the formation of binding sites were first assigned to the GABRA1 sequence used in AlphaFold predictions. Then they were transferred to the reference numbering. For each of the binding sites of interest (and the pore facing amino acids on TM2) the per-amino acid number of variants and the sum of minor allele count per 100,000 were calculated and separately visualized.

### Conformational Analysis

The lists of pocket forming amino acids for each binding site were used to extract the Cɑ coordinates for each individual amino acid in the original PDB file using a Python script. To identify the different local conformations of the protein binding sites, distances and angles formed by the selected Cɑ were calculated using the Python library Biopython (https://biopython.org/). For the site present on a single subunit (5OSC), single subunits were used. For the sites at subunit interfaces, all dimers were used. All datasets of distances and angles (per subunit, or per dimer) have been processed using the principal component analysis (PCA) to reduce the dimensionality to three dimensions. The PCA was performed by using the Python library scikit-learn (Pedregosa et al., 2012).

All structures that are listed in Supplementary Item S1 were used in the analyses, except those that have missing segments or amino acids (4X5T, 6QFA, 4BOT, 4BOR, 4BOI, 4BON, 4BOO, 4AQ5) or are partly unfolded or misfolded (6D6T, 6D6U).

### Homology Modeling and Computational Docking

Homology modeling of GABA_A_R α6 was performed by the use of MODELLER 9.21 (Webb and Sali, 2016). The alignment (which is free of INDELS) was done with Promals3D with 6HUO as the template. All the dockings were performed using GOLD (Jones et al., 1997) from Hermes 2020.2.0. The ligand was set flexible by allowing to flip ring corners and detecting internal H bonds. 300 poses were generated per docking run with the option to generate diverse solutions turned on. The CHEMPLP was set as the main scoring function and chemscore for the rescoring. The best 20 docking poses from both scoring functions were used for further analysis.

### Flexible Side-Chains

site 2 (GlyR α3+/α3−): 5TIO: F13, R27, I28, R29, F32, Y78, L83, D84, L85, D86
site 3 (nAChR α7+/α7−): 7EKT: L235, N236, I244, T273, F275, M276, L277, L278, Q295, M301
site 3 (GABA_A_ β2+/α1−): 6X3T: I228, Q229, L232, M236, T262, N265, D282, L285, M286, F289
site 4 (GABA_A_ α1+/α1−): 5OSB: I238, Q241, V242, W245, F297, I301, T305, Y308, F309, R396
site 5 (GABA_A_ α1): 6HIO: I296, E300, F303, L307, V432, L433, L436, L437, I440, Y441
site 5 (GABA_A_ α6): α6 homology model: F233, F284, S287, E291, K317, I318, Y321, S322, L325, F326 (numbering as in 6HUO, with ICD numbers missing and TM4 numbering offset).

## Results

**FIGURE 2:**
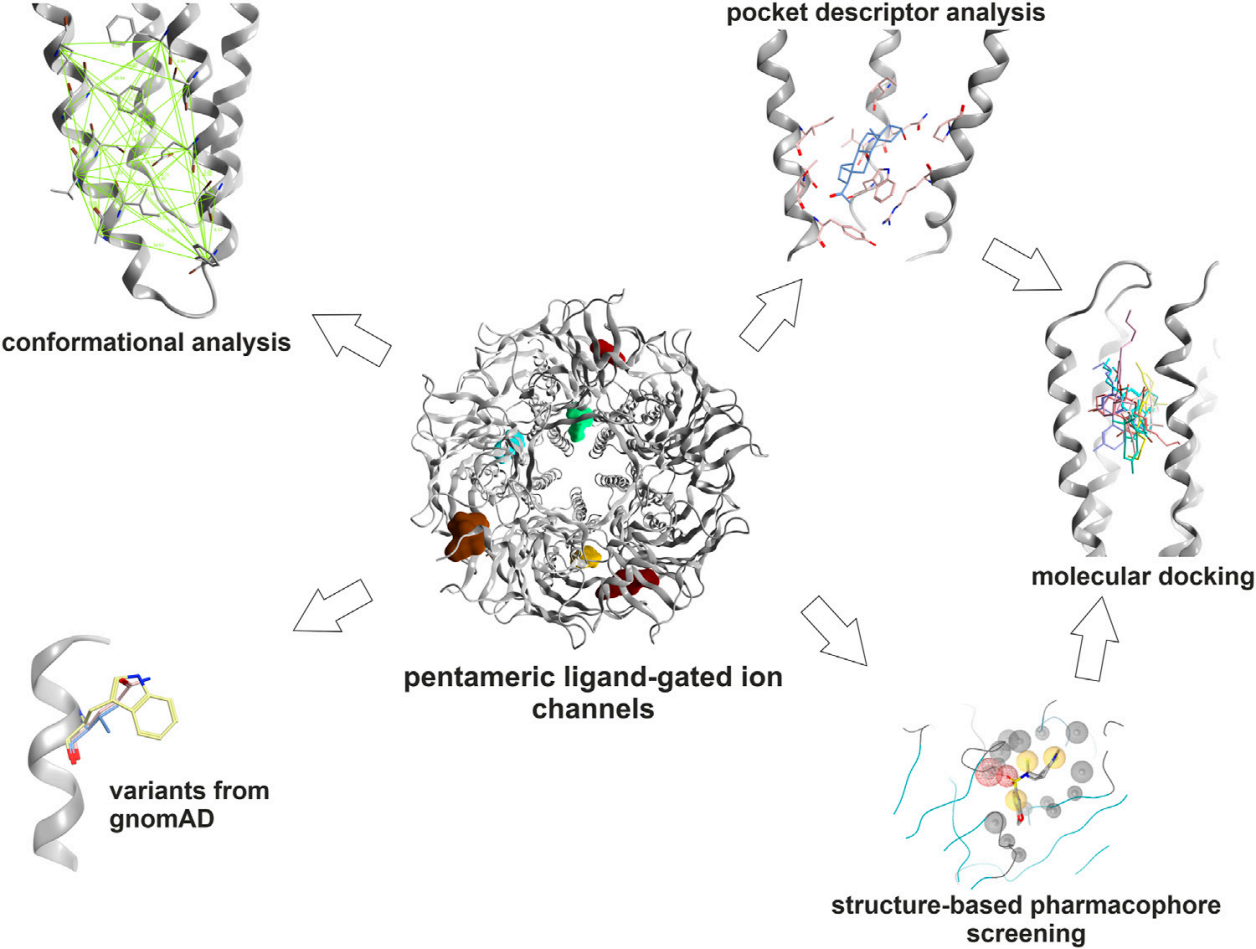
Graphical methods: The binding sites which were selected for this study were subjected to an array of computational tools: Amino acids contributing to the pockets were extracted. Subsequently, local structure based alignments and sequence- to- structure alignments were employed to identify or predict their topological equivalents in the other subunits where possible. These pocket forming segments were then subjected to local sequence similarity and pocket descriptor computation. Hierarchical clustering of pairwise euclidean distances between pocket descriptor coordinates was performed to predict similarities (see *Methods*). Pocket conformations were analyzed in order to guide the selection of structures for computational docking. The relative abundance of variants at and near ligand binding sites was extracted from gnomAD. Structure based pharmacophore screening was employed to derive selected bound state hypotheses to complement the pocket analysis. For dronabinol (the phytocannabinoid Δ^9^-THC), multimodal predictions from literature search, mining of Drug Central, pharmacophore screening and pocket similarity predictions were ranked and strong candidates were followed op with homology modeling and computational docking.

The binding sites for which structural data exists were analyzed from the viewpoint of similarities across the sub-families. The study’s focus is on sites for which no radioligands are available, but structural data allows a variety of *in silico* predictions. Specifically, sites 2, 3, 4 and 5 (Figure 1) were analyzed in more detail with the methods shown in Figure 2 as applicable. All chosen methods except for the computational docking are suitable for simple high throughput *in silico* screenings.

In a first step, the available bound state structures and homologous apo- states were analyzed to generate an exact inventory of binding site segments, and side-chain contributing amino acids. Where structural equivalence allows this, they were extrapolated to subunits with unknown structures. The high structural variability in the ECD (Supplementary Figures S3, S4) leads to ambivalent sequence to structure alignments in several binding site segments for site 1 and 2, but can be performed for smaller subsets of subunits. Pocket descriptor calculation was thus applied to all TMD sites, and to a smaller set of subunits for site 1. Pharmacophore screening of a small library of known binders as reflected by Drug Central was performed for representative models of sites 1, 3, 4 and 5. The predicted pockets for sites 3, 4 and 5 which largely lack INDELS were examined with the two descriptor methods to derive predictions for highly similar pockets. Consensus predictions derived from pharmacophore screening and pocket analysis were examined and followed up with homology modeling and computational docking for the case of the phytocannabinoid Δ^9^-THC. Prior to the selection of structures for computational docking, conformational analysis was performed as ligand bound states often display induced fit conformations, and apo-states can feature collapsed pockets.

Drug effects display large individual variability, ranging from non-responding to rare side effects observed only in a small fraction of treated individuals. As an example, midazolam, which is popular as a sedative for unpleasant medical procedures such as dental work, can induce severe paradoxical effects (Mancuso et al., 2004). Among the possible causes of such effects, genetic variants that directly impact the effect of a drug on its targets can play a role. These variants can be positioned in the binding site and thus affect the interaction of the ligand with the protein. On the other hand, variants positioned near the binding site can affect the transfer of the ligand’s conformational effect. Thus, we also investigated the occurrence and frequency of missense variants in the greater region of drug binding sites.

### Binding Sites in the ECD

The ECD features a modified immunoglobulin fold (Brejc et al., 2001), consisting of several highly conserved segments with rather large variable regions at the N-termini, and interspersed into the domain in multiple places (Supplementary Figures S3, S4). The canonical agonist site is localized at ECD interfaces of specific subunits in all families. Allosteric sites are formed by other specific subunit interfaces, such as the high affinity Bz-binding site of GABA_A_Rs (Figure 1). For the agonist sites of all families, as well as for the Bz-site, high affinity radioligands exist for efficient *in vitro* screenings. The binding site forming segments comprise conserved and variable parts (Supplementary Figures S3, S4), and the variable parts contain fairly large INDELS. Existing structures provide a glimpse into the structural and conformational variability of the canonical interface pockets, which renders them very challenging targets for rapid *in silico* methods due to the ambiguities of the INDEL positioning and the appropriate choice of ligand bound conformations (Puthenkalam et al., 2016). While there is considerable interest in allosteric sites for which radioligands are lacking (Ramerstorfer et al., 2011; Puthenkalam et al., 2016), the need for more elaborate *in silico* methods for suitable INDEL placement go beyond the scope of this study.

For a subset of GABA_A_R receptor subunits which align well, an explorative computation of pocket descriptors was performed. For this, we used existing Bz bound structures and applied the pocket descriptor calculation in order to test and compare [z-Scales(3) and MSWHIM]. As the arrangements of α-β-γ containing receptors are generally assumed to be identical with the known situation for α1-β-γ2 as depicted in Figure 1, and α-β receptors are assumed to have a β subunit in the place of the γ subunit, we constructed all hypothetical α+/β−, β+/α−, α+/γ−, γ+/β− and β+/β− interfaces. Based on the available bound states we extracted the binding site forming sidechains and examined the resulting z-Scales(3) and MSWHIM descriptors (Supplementary Figures S5–S7). Both descriptors separate the GABA binding β+/α− interfaces from the Bz-binding α+/γ− interfaces, thus recapitulating the known pharmacology (Supplementary Figure S5). In line with experimental findings, the modulatory α+/β− interfaces share more properties with the Bz-sites than with the GABA sites (Supplementary Figure S6).

**FIGURE 3:**
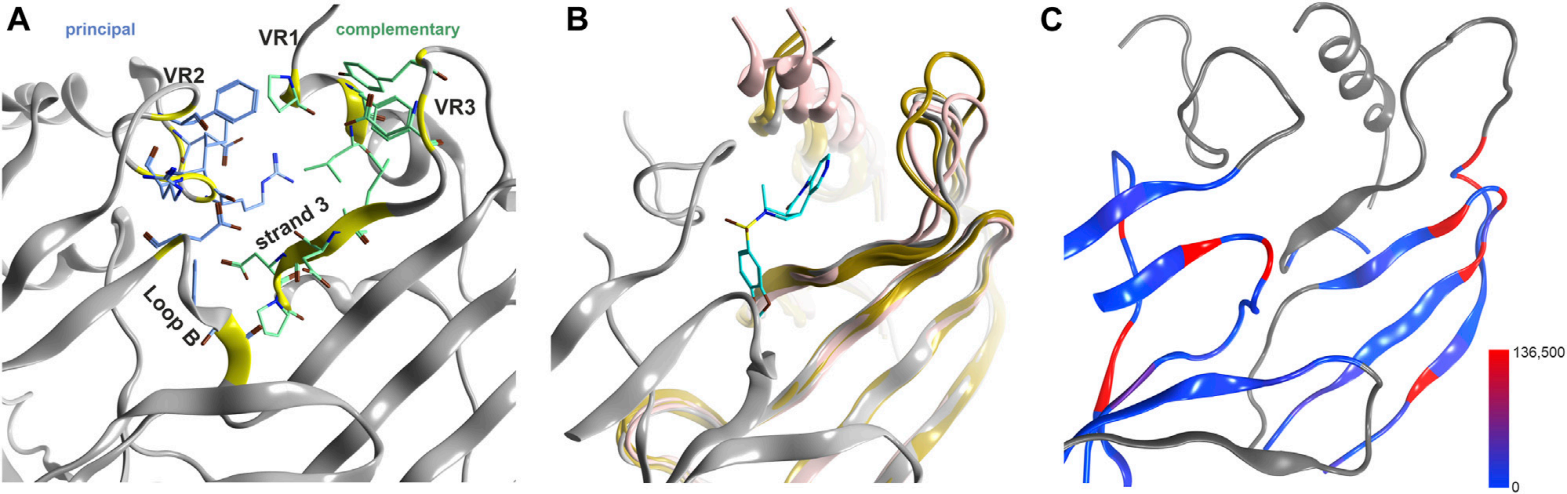
Novel binding site at the upper ECD interface of GlyR α3. **(A)** the binding site from 5TIO with pocket forming segments labeled (see Supplementary Figure S4 for the topology) and amino acids displayed in stick rendering. **(B)** 5TIO (in grey) with the ligand AM-3607 bound at the novel pocket in superposition with closely homologous proteins glycine α2 (5BKF chain D in gold), glycine β (5BKF chain E in blue), GABA_A_ α1 (6X3V chain B in pink) and GABA_A_ β2 (6X3V chain C in red) at the complementary subunit to show the structural variability in VR3. **(C)** Amount of variants across all genes of interest in the region of this binding site mapped onto 7EKT. Ribbon heatmap represents the sum of minor allele counts per 100,000 at each aligned position. Variable regions, where the 44 subunits cannot be aligned to a single reference structure, are shown in gray.

In addition to the canonical interface pocket, a novel pocket has been observed in a crystal structure of a GlyR α3 homopentamer (Figures 1, 3) (Huang et al., 2017). Structural superposition with other superfamily members and sequence to structure alignments indicate that this site comprises conserved and variable segments and considerable structural diversity (Figures 3B,C). Sequence similarity analysis for the conserved parts of this site suggest that homologous sites likely exist at interfaces of other subunits across the entire superfamily. The principal component of GABA_A_R β, δ and θ subunits is most similar, the much more variable complementary component of GABA_A_R β, δ, θ and γ subunits are the strongest candidates for sites that might be used by ligands common to multiple family members. However, the overall size and shape is strongly affected by the variable region 3. A pharmacophore screen into this site provides first insights into the chemical space of candidate ligands. Of the small library we used here (Supplementary Item S2; Supplementary Figure S11), 46 drugs with a wide range of chemical scaffolds generated hits in this site (Supplementary Figures S12, S13). This result is suggestive of a possible contribution from this novel binding site to the polypharmacology within the pLGICs.

Generally, the large number of variable segments present at and near ECD binding site forming regions (Supplementary Figures S3, S4) indicates that these binding sites are of concern only for limited polypharmacology (e.g., the interactions of Bz-site binders with the homologous GABA_A_ receptor α+/β− interfaces (Iorio et al., 2020), or for the interactions of e.g., taurine with glycine- and GABA_A_ receptors. The mapping of variants to a representative structure is also not possible for large parts of the ECD and needs to be considered per- subunit. An overview of the variable segments is provided in Supplementary Figure S8.

### Binding Sites in the TMD

The most conserved domain is the TMD, where all anion channels share >75% sequence similarity. Among nAChR subunits, conservation is as high, but 5-HT_3_Rs and the ZAC- protein feature remarkable variability within the 5-HT_3_ family and also relative to nAChR subunits (Supplementary Figure S9). The similarity index for the whole domain is not informative about the individual binding sites, it is only suggesting that the TMD is likely to contain binding sites conserved across many superfamily members. This is also evidenced by the observation that the channel blocker picrotoxin binds to most superfamily members with comparable affinity (Erkkila et al., 2004; Das and Dillon, 2003; Das and Dillon, 2005; Yang et al., 2007; Thompson et al., 2010). The channel blocker site is the only site in the TMD for which radioligands exist, thus, there is a big need to better characterize the other allosteric sites present in this domain and to develop reliable *in silico* screening methods for these.

**FIGURE 4:**
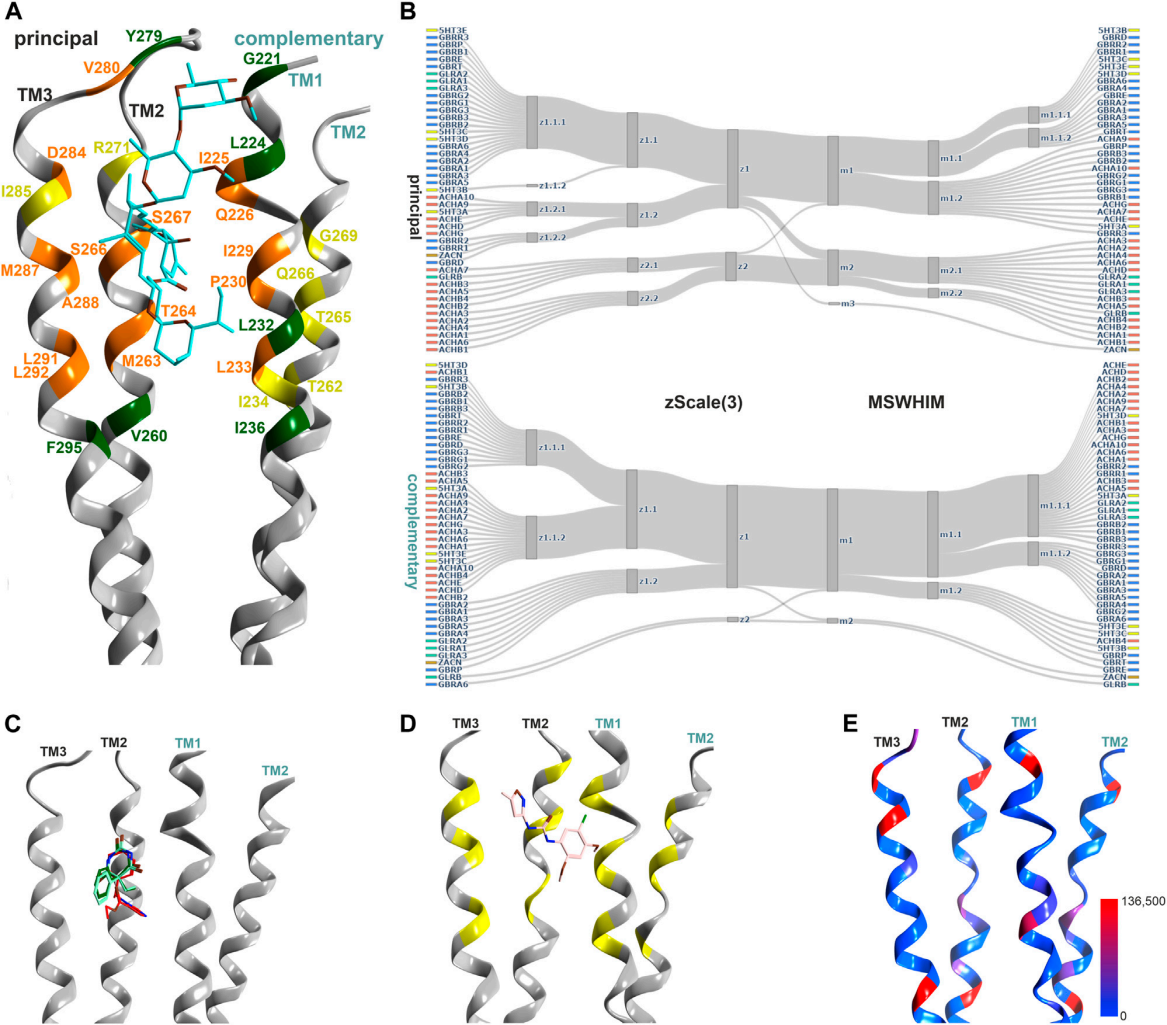
Representative structures of the upper TMD interface pocket, and descriptor analysis: **(A)** Overview of the TMD interface with ivermectin (cyan sticks) in 5VDH. The ribbon segments that provide ligand contacts with ivermectin and with small molecules are orange, those with small molecule contacts only are yellow, and those with ivermectin only contacts are green. Amino acid numbering as in 5VDH. **(B)** Comparison diagrams for the subsite used by small molecules derived from hierarchical clustering of z-Scale(3) and MSWHIM descriptors as estimates for pocket similarities. Supplementary Figure S14A depicts the full dendrograms, Supplementary Figures S14B,C depict the analogous comparison for the ivermectin site. **(C)** Ribbon of the GABA_A_ R β+/α− structure with etomidate (6X3V in red), phenobarbital (6X3W in light- and dark-green). **(D)** 7EKT with PNU-120596. The yellow positions on the ribbon indicate the binding site forming segments which were used for panel **(B)**. **(E)** Amount of variants across all genes of interest in the region of this binding site mapped onto 7EKT. Ribbon heatmap represents the sum of minor allele counts per 100,000 at each aligned position. See Supplementary Figures S18,S19 for detailed heatmaps.

The best characterized site in the TMD is localized at the upper portion of the subunit interfaces, where 9 ligand bound structures exist which cover GABA_A_Rs, nAChRs and GlyRs (Figure 4; Supplementary Item S1). The TMD is nearly free of INDELS, and structurally very conserved. Thus, it is ideally suited to compare pockets on the basis of local sequence similarity, and with the descriptor methods that have been demonstrated as computationally very inexpensive tools to estimate overlapping chemical spaces across pockets. The two descriptors used here (z- Scale(3) and MSWHIM) broadly agree on the similarities within and across family members for both the principal and the complementary components of this site (Figure 4B). The principal component contributing subunits fall into two clusters, one containing all GABA_A_R subunits and individual members from all other families. The smaller cluster comprises the β subunits and the majority of the α subunits from the nAChR family, alongside with the GlyR β subunit. The complementary component segments cluster differently, with almost all subunits sharing high similarity for the MSWHIM descriptor with only the ZAC protein and the GLRB protein forming a small separate cluster (Figure 4B; Supplementary Figure S14B). This suggests that the upper TMD interface is likely to have very broadly overlapping ligands for many superfamily members. This analysis was limited to the amino acids that contribute to the binding sites for small molecules as determined from 7EKT, 6HUO, 6X3V, 6X3X, 6X3T and 6X3W. In order to gauge the performance for more detailed predictions, we re-analyzed the amino acid lists for the upper TMD pockets of specific GABA_A_Rs, as was done for the ECD interface pockets. The resulting clusters follow closely the known distinctions between barbiturate- preferring and etomidate- selective pockets, see Supplementary Figure S14A: Both descriptors generate a homogenous α+/β− cluster, reflecting one of the barbiturate preferring groups. The other barbiturate preferring interface, localized at γ+/β− interfaces, forms clusters containing several β+/α− interfaces for the z-Scale(3) and MSWHIM, suggesting that barbiturate- and etomidate- preferring pockets might have common ligands. This is in fact observed for the low affinity diazepam sites (Kim et al., 2020). The etomidate insensitive β1+/α− form a separate cluster, again recapitulating the known pharmacology.

The ivermectin binding site, also localized at the upper TMD interface, is larger and overlaps partly with the sites for the small molecule ligands discussed above, see Figure 4. In our pharmacophore screen, we observed that a stringent screen into ivermectin bound state, as expected, cannot retrieve small molecule binders, but a less stringent screen will produce many weak hits and is prone to false negatives. Applying the descriptor analysis to the amino acids that form ivermectin contacts results in partly controversial findings between the two used methods, see Supplementary Figure S14C: For example, the principal component of ZACN clusters with GABA_A_R subunits in the z-Scale(3) and MSWHIM dataset. It would be interesting to have experimental data with which the results could be validated, requiring more subtypes of pLGICs to be tested. At the present time, several high and low affinity interactions are known suggesting a smaller group of high affinity sites, and a very broadly promiscuoius profile in the micromolar range (Lynagh and Lynch, 2010).

The high similarity for the pockets not only within, but also across families suggests common ligands. Most of the experimental structures that can be employed for structure-based pharmacophore screens are ligand-bound GABA_A_Rs. Since homologous upper TMD binding pockets exist within receptor families (low affinity diazepam/etomidate site and both barbiturate sites) as well as in different pLGIC family members (7EKT for nAChR and 5VDH for glycine receptors), multiple pharmacophore screens to this binding site were performed (Supplementary Figures S11, S12). Most pharmacophores derived from ligand bound GABA_A_Rs predict a wide range of drugs to be potentially accommodated by upper TMD interface sites (Supplementary Figure S12), in agreement with the promiscuity of this pocket according to literature (Iorio et al., 2020). All of the 18 substances that produced drug-target hits from pharmacophore screens derived from the homologous site in the nAChR α7 receptor (PDB ID: 7EKT) also generated a hit in at least one homologous site of GABA_A_Rs or GlyRs. As an example, the plant compound dronabinol (Δ^9^-THC), was predicted to potentially bind at the upper TMD sites of nAChRs, GABA_A_Rs and GlyRs (Supplementary Figures S11, S12).

**FIGURE 5:**
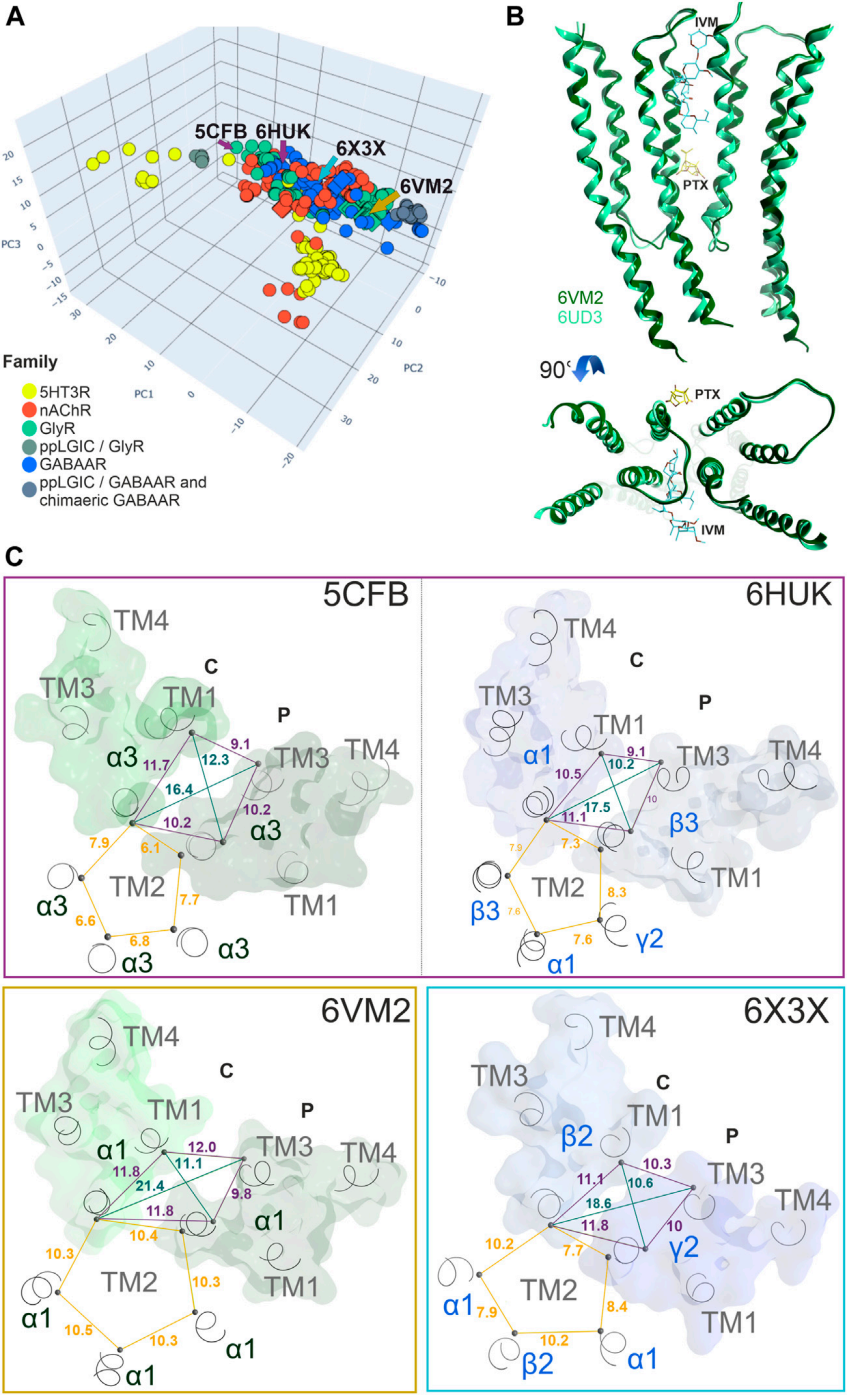
Conformation analysis of the upper TMD interface pocket: **(A)** Scatter plot of the pocket conformations. Each dot represents the results for an individual upper TMD interface, diamond shaped symbols indicate the presence of a ligand in the analysed site. The colour of the dots indicates the protein family as defined in the legend, gray is used for diverse chimeras. ppLGIC: procaryotic pentameric ligand gated ion channel. Arrows and PDB IDs indicate the localization of structures which are rendered in panels **(B)** and **(C)**, arrow and box colours match across the panels. **(B)** Superposition and comparison of two interface forming subunits of the ivermectin bound Gly R structure 6VM2 and the picrotoxin bound 6UD3, featuring nearly identical conformations of the entire TMD. **(C)** Different conformations result in different distances between alpha carbon atoms: A plane through the upper TMD is depicted with all five TM2 segments and two subunits. Distances between alpha carbons are shown to illustrate backbone conformation, where yellow lines are distances between homologous pore forming TM2 residues as an indication for asymmetry, purple lines are distances between pocket forming residues defining the circumference, and green lines the pocket “diagonals”.

Conformational analysis indicates, in line with previous work (Puthenkalam et al., 2016), substantial flexibility of this region. Structures of all family members share overlapping conformations, irrespective of the occupancy of the upper TMD binding site (Figure 5A), but for the cation channel branch of the superfamily, additional conformations have been observed. In these, the RMSD of the pocket backbone differs by up to 2.8 Å from the more frequently observed conformations (Supplementary Figure S17A). The majority of structures populate a conformation space shared by receptors with ligands present at one or several upper TMD interfaces and receptors without ligands in this pocket (apo- site 3, Figures 5B,C). This indicates that ligands of site 3 do not lead to induced fit conformations. Intriguingly, the picrotoxin bound 6UD3 and an ivermectin bound 6VM2 GlyRα1 structure display nearly identical conformation not only of site 3 in the apo- state versus ivermectin- bound state, but of the entire TMD. In contrast, a very different conformation of the upper TMD interface is featured in the GlyR α3 5CFB in complex with the orthosteric antagonist strychnine, see Figures 5A,C. Following on this observation we selected further structures from the region around 5CFB and noted many instances of orthosteric antagonist bound structures in this region of the conformation space, including the bicuculline bound 6HUK β3+/α1− interface, Figures 5A,C and Supplementary Figure S17A. For nAChRs, we observe the same dominant effect of orthosteric antagonists on the TMD pocket conformation, see Supplementary Figure S17A. The strongest effect appears to be a contraction of the upper TMD pocket, mainly on the “long” diagonal between complementary TM2 and principal TM3, see Figure 5C. These observations suggest that orthosteric ligands are dominant drivers of the conformation in the upper TMD, which is further weakly influenced by other factors. Of further interest is the high degree of asymmetry relative to a pseudo symmetrical pentagon that occurs in many structures, such as the 6X3X GABA_A_R with diazepam present in the upper TMD, Figure 5C. Interestingly, we find the conformations of chimeric constructs with ECDs from bacterial homologues to occupy distinct regions of the conformation space (Figure 5A; Supplementary Figure S17A).

**FIGURE 6:**
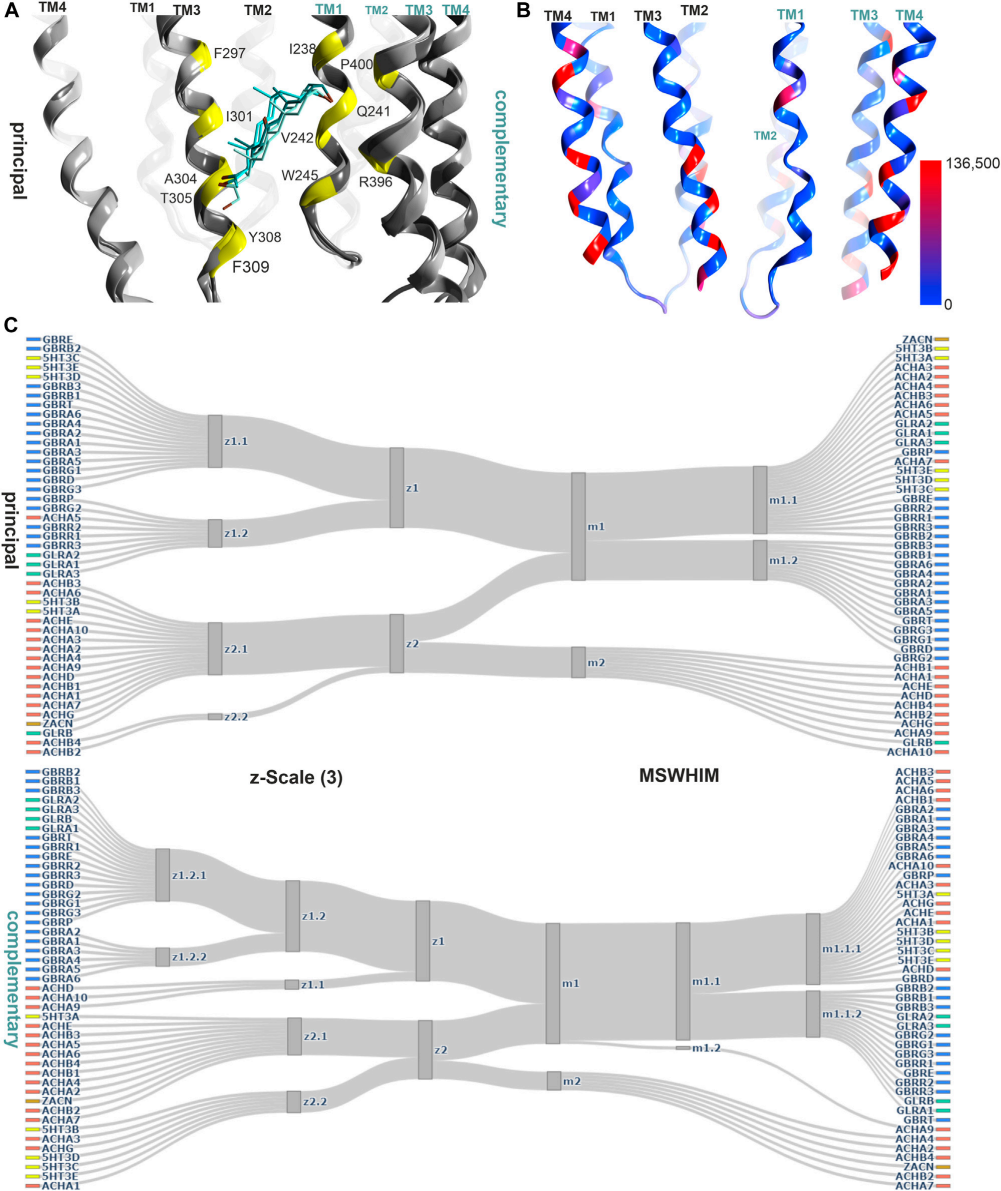
Representative structures of the lower TMD interface steroid pocket, and descriptor analysis: **(A)** Ribbon of the structures with modulatory steroids at the lower TMD interface: 6CDU—alphaxalone (dark green), 5O8F—pregnanolone (cyan), 5OSB—THDOC (light green). The yellow places on the ribbon indicate the binding site forming positions which were used for the pocket analysis computations. **(B)** Amount of variants across all genes of interest in the region of this binding site mapped onto 7EKT. Ribbon heatmap represents the sum of minor allele counts per 100,000 at each aligned position. See Supplementary Figures S18,S19 for detailed heatmaps. **(C)** Comparison diagrams derived from hierarchical clustering of z- Scale(3) and MSWHIM descriptors as estimates for similarities. Supplementary Figures S15,S16 depict the full dendrograms.

The lower TMD interface also contains a binding site, which has been characterized as a modulatory site for neuroactive steroids (Figure 6) (Laverty et al., 2017; Miller et al., 2017; Chen et al., 2018). There is big interest in the development of neurosteroid-mimetics as putative drug candidates (Blanco et al., 2018), which also raises the question how prone to cross-reactivity such ligands might be. The analysis we performed based on local sequence similarity and pocket- descriptors indicates that the 45 subunits fall into two clusters, which are similar for the principal and the complementary component (Figure 5C; Supplementary Figure S15). In GABA_A_Rs receptors, α subunits contribute a glutamine sidechain from TM1, which drives the high efficacy modulation and is absent from all other subunits. The remainder of the site is quite conserved for GABA_A_Rs and GlyR α subunits, and a few members from the cation permeable family members fall into this cluster. Thus, the unique high efficacy determinant present exclusively on GABA_A_R α subunits might render this site sufficiently unique to be of less concern for toxicological alerts.

**FIGURE 7:**
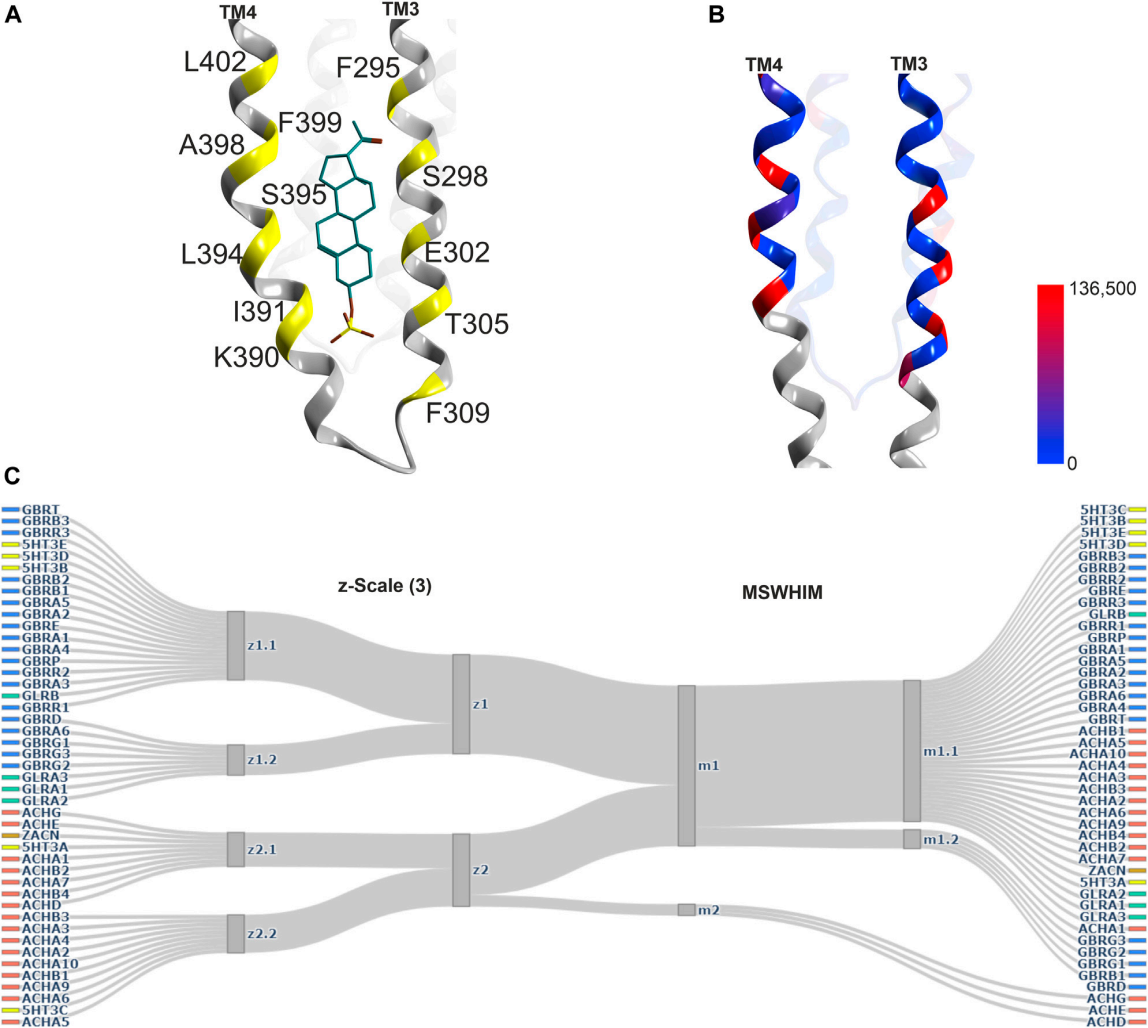
Representative structures of the lipid/TM3/TM4 associated site, and descriptor analysis: **(A)** Ribbon of the 5OSC structure with pregnanolone sulfate bound. The yellow places on the ribbon indicate the binding site forming positions which were used for the pocket analysis computations. **(B)** Amount of variants across all genes of interest in the region of this binding site mapped onto 7EKT. Ribbon heatmap represents the sum of minor allele counts per 100,000 at each aligned position. See Supplementary Figures S18,S19 for detailed heatmaps. **(D)** Comparison diagrams derived from hierarchical clustering of z-Scale(3) and MSWHIM descriptors as estimates for similarities. Supplementary Figure 16 depicts the full dendrograms.

An additional steroid binding site has been described, which is localized in a position between the lower end of TM3 and TM4 of a GABA_A_R α1− subunit and the lipid collar, the TM3/TM4 lipid associated site (Miller et al., 2017, see Figure 7). Interestingly, in another structure, a PIP2 molecule was found in an overlapping localization (Laverty et al., 2019). This region has been subjected to mutational studies aimed at the identification of binding sites for endo- and phytocannabinoids (Sigel et al., 2011; Xiong et al., 2011; Bakas et al., 2017), and is thus considered to be an interaction site for both endogenous and exogenous lipophilic ligands.

The descriptor analysis does not give a consensus picture, as the z-Scales(3) predicts two clusters (one comprising all nAChR subunits, the A and C subunits of the 5-HT_3_R and the ZAC protein), while MSWHIM predicts high similarity across the entire superfamily except for ACHD, ACHE and ACHG (Figure 7C; Supplementary Figure S16).

**FIGURE 8:**
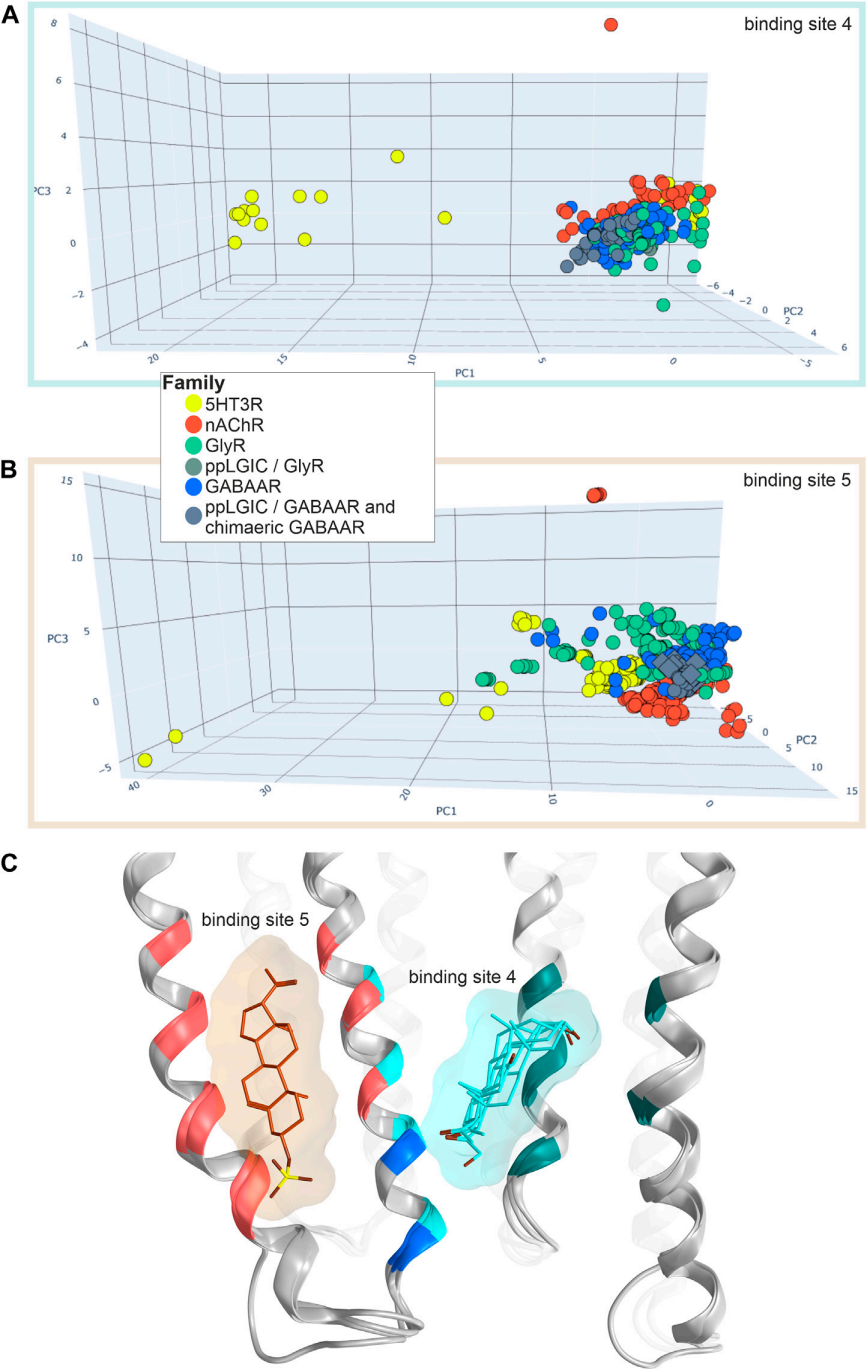
Conformation analysis of the lower TMD with sites 4 and 5: **(A,B)** Scatter plots of the pocket conformations. Each dot represents the results for an individual site, diamond shaped symbols indicate the presence of a ligand in the analysed site. The colour of the dots indicates the protein family as defined in the legend, gray is used for diverse chimeras. ppLGIC: procaryotic pentameric ligand gated ion channel. **(C)** Superposition and comparison of 5OSB, 5OSC, 5O8F and 6CDU. Pregnenolone sulfate bound to site 5 is rendered in brown sticks, THDOC, alphaxalone and pregnenolone bound at site 4 in cyan sticks. Ribbon colouring indicates contributions to site 5 (red), 4 (cyan) or both (blue).

Conformation analysis of the lower TMD reveals that the majority of structures of all superfamily members cluster very tightly for both sites, indicative of very similar conformations irrespective of the presence or absence of ligands in this site, see Figure 8. Additionally, a few structures of nAChRs and 5-HT_3_ARs adopt a very different conformation with a local RMSD up to 2.6 Å (Supplementary Figure S17C). In the case of the lipid associated site, this reflects very different conformations of the TM4 helices in the different experimental structures, resulting in local RMSD differences > 3 Å (Supplementary Figure S17D). For this site, the 5-HT_3_R structures are uniquely different from all others, see Figure 8B and Supplementary Figure S17D. Surprisingly, the lower TMD ligands seem to not induce any induced fit at all, as the structure with the inhibitory steroid preganolone sulfate in site 5 (5OSC) and all structures with modulatory steroids in sites 4 (5OSB, 6CDU, and 5O8F) superpose very tightly, see Figure 8C.

### Cross-Reactivity Within Non-Homologous Sites in Individual Receptor Pentamers

An unexpected finding in our pharmacophore screens is the extensive overlap of hits for the novel ECD site 2 and the upper TMD interface sites. This raises the question whether it reflects spurious hits, or hints at a genuine overlap of ligand space shared by non-homologous pockets. A similar situation is well established for GABA_A_ receptors: Diazepam binds with high affinity at the canonical ECD α+/γ− interfaces of certain receptor subtypes, and additionally with lower affinity at two of the upper TMD sites (Masiulis et al., 2019; Kim et al., 2020).

In this study, we did not include the canonical ECD interface sites, as the large number of distinct interfaces and the multiple segments with INDELS would have exceeded the scope of this paper. Our results, however, suggest that the multi-site usage of ligands as displayed by diazepam is not limited to the sites 1 and 3, but may also affect other combinations of binding sites. For the sites localized in the TMD, structural data is yet not very suggestive of ligand promiscuity between the different sites (3, 4, and 5). In contrast, photoaffinity studies strongly suggest that steroid derivatives have multiple binding sites not limited to the lower TMD, but also in the upper TMD (Chen et al., 2019; Sugasawa et al., 2019). It would be very interesting to find out whether endogenous ligands also interact with multiple sites in the ECD and TMD.

While our limited pharmacophore screen does not suggest steroids as hits for the upper TMD sites, we note that 17 compounds, representing considerable chemical diversity, are predicted with moderate to strong scores to match with the pharmacophore models from all three types of sites. This is the case for dronabinol (Δ^9^-THC), another example is the 5-HT_3_R interacting antiemetic metoclopramide (Supplementary Figures S11, S12). The latter thus is potentially another example for a compound which interacts with the canonical ECD interface site and additional sites in the TMD, akin to the Bzs. In the screens performed here, we also note that a fraction of the screened Bzs, as well as the Bz-site ligand alpidem, score with 0.7 or higher for all three TMD sites in at least one family member (Supplementary Figures S11, S12).

### Assessing Candidate Binding Sites for the Phytocannabinoid Δ^9^-THC

For phytocannabinoids, the literature demonstrates interactions with multiple family members (Barann et al., 2002; Oz et al., 2004; Yang et al., 2008; Yang et al., 2010; Xiong et al., 2011; Bakas et al., 2017; Schmiedhofer, 2017). Effects differ depending on the protein subtype, as well as the cannabinoid molecule. For example, Δ^9^-THC is relatively ineffective at inhibiting α7 nAChRs, while inhibiting 5-HT_3_Rs with similar potency to CB_1_ receptors (Barann et al., 2002; Oz et al., 2004). On the other hand, CBD and Δ^9^-THC are potentiating GlyRs and most GABA_A_R subtypes (Xiong et al., 2011; Schmiedhofer, 2017). One candidate binding site has been delineated by mutational studies: In GlyR α1 and α3 subunits, a site which was proposed to correspond with the later described pregnanolone sulfate site on GABA_A_ R α1 subunits in the 5OSC structure, was shown to mediate a large fraction of a strong PAM effect elicited by CBD (Xiong et al., 2011). For 5-HT_3_Rs an allosteric modulatory binding site has been suggested (Barann et al., 2002).

**FIGURE 9:**
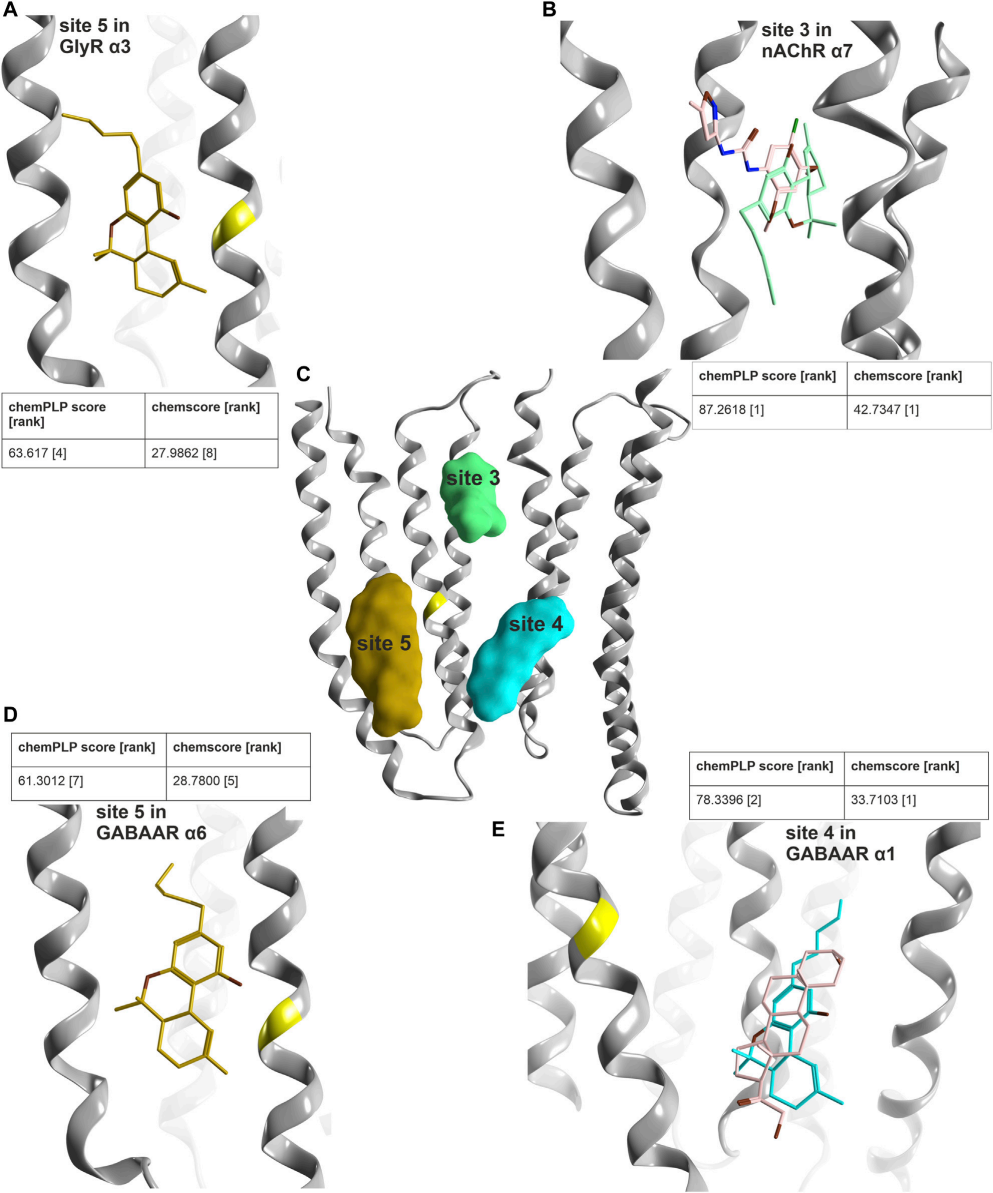
Computational docking into candidate binding sites for Δ^9^-THC. **(A)** Consensus binding mode for site 5 in the GlyR α3 subunit from 5VDI which is also present in the GABA_A_R α6 docking in panel **(D)**. **(B)** The highest ranked consensus scored representative binding mode from the docking into site 3 from 7EKT with Δ^9^-THC in blue superposed with PNU-120596 in pink. **(C)** All three sites of interest displayed on a representative ribbon structure with GlyR α3 Ser307 indicated by a yellow segment on TM3. **(D)** Docking result of Δ^9^-THC into site 5 of a GABA_A_R α6 homology model. **(E)** Representative binding mode from the docking into 5OSB superposed with THDOC in pink.

Our PH4 screen indicates matches for Δ^9^-THC with the TM3/TM4 lipid associated site 5, as well as with most upper TMD interface sites (3), and with the steroid bound lower TMD-interface (site 4) (Supplementary Figure S12). In addition, another match occurred for the novel upper ECD interface site. We thus performed an exploratory computational docking screen into all matched sites. In turn, we ruled out the upper ECD interface as it displays multiple clashes, and the docking results cannot recapitulate the overlap of the pharmacophore features at all. This leaves the binding sites in the TMD as candidates. We again turned to the literature to search for evidence on binding sites, and to prioritize docking for family members with known effects. For GABA_A_Rs, both CBD and Δ^9^-THC have been investigated in multiple subunit combinations (Bakas et al., 2017; Schmiedhofer, 2017). Both phytocannabinoids elicit similar effects in most GABA_A_R subunit combinations that were tested, enhancing GABA currents in most of them and reducing currents in β1-containing assemblies (Schmiedhofer, 2017). A mutation in TM3 of the GlyR α3 subunit (Xiong et al., 2011) is localized in a position shared by sites 4 and 5, and in close proximity to site 3 (Figure 9).

In order to select subunits for further computational docking, we examined the predicted similarity between GlyR α3 and other candidate subunits. The descriptor analysis of the candidate sites is ambiguous for the upper TMD interface, where the two descriptors place GlyR α3 into different clusters. The placement by MSWHIM with nAChR subunits (Supplementary Figure S14b) would be consistent with the strong hit in the 7EKT structure’s site 3, and was thus followed up in more detail. For the lower TMD interface site, both descriptors cluster the GlyR α3 subunit with GABA_A_R subunits (Supplementary Figure S15), in line with the hit for this steroid binding site. Interestingly, for the TM3/TM4 lipid associated site, where MSWHIM suggests broad conservation, the z-Scales(3) places GlyR α3 into a cluster with the GABA_A_R α6 subunit (Supplementary Figure S16). This is interesting because the current enhancement by Δ^9^-THC that was observed in a pilot experimental study is stronger for α6-containing assemblies compared to other α- isoforms (Schmiedhofer, 2017).

In order to derive testable structural hypotheses for pockets with combined experimental and computational evidence, we generated docking poses for site 3 in the nAChR α7 homomer, in the steroid bound site 4 of the GABA_A_R lower TMD α1+/α1− (original PDBID: 5OSB), and in site 5 in GlyR α3 [to compare with the CBD docking from (Xiong et al., 2011)] as well as in a homology model of GABA_A_R α6. The docking results for these four cases were then subjected to two scoring functions to obtain poses with highly ranked consensus scores (Figure 9).

For all four dockings, multiple binding modes with high scores were obtained with both scoring functions. With additional restraints coming from indirect experimental evidence, poses were identified for site 5 which feature GlyR α3 Ser307 in close proximity to the benzo[C]-chromen-1-ol hydroxy group on the ligand, which is in agreement with the pharmacophore screen as well. In addition, this pose has a high overlap with a pose in the GABA_A_R α6 model, where both are among the top ten of both scoring functions, see Figure 9. For the docking into the site 3 of the nAChR α7 homomer, the top pose of both scoring functions features high overlap with the PAM ligand of the experimental structure, and thus is in agreement with all available evidence. Lastly, for the results obtained for site 4, the best ranked binding mode overlaps very closely with the ligand from the experimental structure (Figure 9E). An alternative binding mode brings the ligand closer to the TM3 of the principal subunit and the site of mutagenesis (Supplementary Figure S20) (Xiong et al., 2011). These structural hypotheses can be used to guide further mutational studies, see Supplementary Figure S21.

## Discussion

pLGICs mediate drug induced seizures and convulsions as well as a broad range of neuropsychiatric adverse effects impacting on sleep, vigilance, mood, memory, autonomic NS functions, and many more, including indirect effects on peripheral nerve regeneration. The NeuroDeRisk project (https://neuroderisk.eu/) aims to improve *in silico* methods for the efficient prediction of such liabilities. The need for such methods is underscored by the large number of allosteric binding sites present on pLGICs, where the development of *in vitro* screening tools such as specific high affinity radioligands is lagging behind. We thus took advantage of the recent surge in structural data and compiled an inventory of allosteric sites for further study.

A multitude of molecules and drugs exist that target several family members. Histamine is a positive modulator of some GABA_A_R subtypes, whereas it was shown to inhibit GlyRs currents with low potency (Breitinger and Breitinger, 2020). Similarly, neuroactive steroids are potent positive or negative allosteric modulators of GABA_A_Rs, while in contrast inhibiting glycine elicited chloride current (Breitinger and Breitinger, 2020). The non-steroidal anti-inflammatory drug niflumic acid, which also binds to GABA_A_Rs where it acts as a positive or negative modulator depending on the subtype (Rossokhin et al., 2019), inhibits GlyRs (Breitinger and Breitinger, 2020).

Apart from their well-recognized use as antiemetics, preclinical studies have suggested a possible therapeutic use of 5-HT_3_ receptor antagonists in depression, substance abuse, cognitive and psychiatric disorders, and pain (Costall and Naylor, 2004; Thompson and Lummis, 2007; Rajkumar and Mahesh, 2010; Walstab et al., 2010). However, clinical studies have so far failed to substantiate the use of 5-HT_3_ receptor antagonists in the treatment of CNS disorders (Greenshaw and Silverstone, 1997). Interestingly, the 5-HT_3_ inhibiting antiemetic therapeutic tropisetrone potentiates glycine elicited effects of some GlyR subtypes (Breitinger and Breitinger, 2020). The literature thus provides many examples of largely unwanted promiscuity of ligands across the pLGIC families, resulting in a need to study it systematically and to develop *in vitro* tools for ligand interaction sites which are highly conserved among the pentameric ligand gated ion channel superfamily.

Several methods are available to perform computationally inexpensive, and thus high throughput, *in silico* predictions for multiple aspects of ligand-protein interactions and the properties and target space of a binding site with known structure. Pharmacophore screening has a long and successful history and serves well to rank libraries of test compounds as potential hits for a site which can be characterized by mandatory and optional interaction features. By itself, the method has known limitations (Kaserer et al., 2015): The stringency of the screen needs to be carefully optimized for each application in order to optimize enrichment. Here, we reasoned that rapid, simple screens with moderate optimization can generate useful hypotheses, and that the combination of multiple *in silico* methods that cover different aspects of the protein-ligand interaction theme can be combined to produce ranked toxicological alerts for more elaborate follow-up investigations. In this vein, less strict screenings and accepting false positive hits is a compromise that shifts a more costly problem of false negatives into a less costly acceptance of true hits mixed with false positives in the “early alert” stage.

If structural information is available in sufficient amount and quality, *in silico* methods that result in simple models of the binding sites can be added to the workflow and further prioritize the experimental follow-up work. A large protein superfamily, such as the pLGICs with 45 human subunits that comprise four families of transmitter receptors, is inherently prone to issues with polypharmacology. The efforts that would be needed for systematic *in vitro* testing of drug candidates on all proteins in the superfamily are not feasible. Given enough experimental structures, ligand binding pockets can be characterized in terms of their structural variability. Currently, available data shows that binding sites present in the ECD of pLGICs are composed of a mixture of conserved and variable protein segments, and thus ligand promiscuity is theoretically limited to well-defined groups of family members. In contrast, binding sites in the TMD share high structural conservation and differ only in sidechains and a few very minor INDEL bearing segments. This is an ideal scenario for the use of methods which rest on the properties which amino acid sidechains contribute to ligand binding pockets independent of their 3D arrangement. Various descriptors have been developed and are widely used to predict properties of peptides, but the concept is increasingly also applied to binding sites (van Westen et al., 2013). The application of descriptors to binding site forming amino acids at the canonical ECD interface site and the upper TMD “anaesthetics” sites of GABA_A_Rs demonstrates that the gross pharmacology is recapitulated, but also that the method is very sensitive to the selected amino acids. If used to predict shared ligand space of a pocket, it is essential to use strictly those amino acids that contribute with sidechains to the pocket in question, which is a considerable limitation for practical applications. Thus, for its routine use, automated extraction of binding site forming sidechains and separate predictions for smaller and larger ligands would still need to be developed and benchmarked. Here, we used the results to guide the selection of candidate pockets for the exploratory computational docking that was performed for Δ^9^-THC.

Prior to embarking on computationally more expensive methods such as more elaborate computational docking and different types of molecular dynamics simulations, it pays off to select among the experimental structures those that are suited to address a given scientific question. One of the inherent limitations is the static nature of methods such as cryo-EM and X-ray crystallography, which captures proteins in long-lived conformational states. The automated analysis of protein conformations which was employed here provides a bias-free approach to compare the pocket of interest across all proteins in which the pocket is found, independent of absence or presence of ligands, and independent of global protein conformations (such as closed- desensitized). An interesting finding along these lines was the dominant influence of ECD ligands on the conformations of TMD binding sites, and even more strikingly, the apparent absence of steroid impact on the TMD conformations (see Figures 5, 8). This underscores the importance to consider all available structures as templates for high throughput methods such as structure based pharmacophore screening, and the inherent limitations of static structural snapshots. The increase in computational performance we currently witness may soon allow certain MD methods to be integrated into high throughput workflows, but at this time, MD still cannot be termed a fast computational technique.

Evidence accumulated that ligands display two types of distinct cross-reactivity: Interactions with homologous pockets of other family members, and interactions with non-homologous pockets in the same receptor. Benzodiazepines, and likely many other compounds interact with multiple sites in the same receptor. In the case of Bz-site ligands, the differences in affinity seem to be high, but data still is scarce (Iorio et al., 2020). While the small scale pharmacophore screen we performed here has clear limitations due to high heterogeneity in the resolution and number of available structures per binding site and the small size of the library, as well as lacking decoys, it does recapitulate known findings well, and it does suggest that the ligand promiscuity as depicted by the data at DrugCentral is severely underestimated (Supplementary Figure S13).

Many of the screened compounds are seen to be moderately or highly scored hits at two and more binding sites, including dronabinol (the trade name of a specific Δ^9^-THC formulation). Since the literature confirms that it has functional effects on all pLGIC families (Barann et al., 2002; Oz et al., 2004; Yang et al., 2008; Yang et al., 2010; Xiong et al., 2011; Bakas et al., 2017; Schmiedhofer, 2017), we explored these hits in more detail. The computational docking results are consistent with most of the high scoring pharmacophore hits, leaving us with three candidate interaction sites for this phytocannabinoid in the pLGICs—the upper and lower TMD interface sites, and the TM3/TM4 lipid associated site. The mutational work which was performed for CBD in previous studies (Xiong et al., 2011; Bakas et al., 2017) probed an amino acid which is localized in a position in close proximity to all three of these sites, thus, further mutational work should be structure guided and probe amino acids that are unique to the individual pockets (see Supplementary Figure S21). The published functional studies show a mix of enhancement or reduction of agonist mediated effects (Barann et al., 2002; Oz et al., 2004; Yang et al., 2008; Yang et al., 2010; Xiong et al., 2011; Bakas et al., 2017; Schmiedhofer, 2017). Of note, in GABA_A_R a limited pilot study observed the net effect to depend on the isoform of α and β subunits (Schmiedhofer, 2017). This is more compatible with a site of action at a subunit interface, or with the use of multiple binding sites in the same receptor. The GABA_A_R β isoforms are known for a single amino acid difference in the upper TMD interface complementary component, but also feature different amino acids in the TM3/TM4 segments that contribute to an endocannabinoid site in the β2 subunit (Sigel et al., 2011). Thus, further experimental studies are needed to clarify the mechanism of action by which Δ^9^-THC modulates 5-HT_3_R, nAChR, GlyR and GABA_A_Rs. Future studies can be guided by the bound state models presented here.

The panel of methods employed in this study, which we benchmarked with a small library of drugs and selected showcases, can deliver useful toxicological alerts with inexpensive *in silico* toolchains that are easy to implement with many available software packages. For proteins with many allosteric sites, multimodal computational screening can help to prioritize the slow and expensive functional studies that need to be performed when radioligands are lacking.

## Data Availability

The conformational analysis scripts can be found under https://github.com/FilipKon/CoDiP. To see only the results (3D scatter plots) and distances with the corresponding amino acids, visit https://fkoniuszewski.pythonanywhere.com/. The scripts used to compare the protein sequences and the variant analysis are available at https://github.com/JureFabjan/GABR_genetics. Other data is available upon request to the corresponding author.

## Supplementary Material

Supplementary Figure S1 Superposed structures and their pockets derived from remote homologues. Chlorpromazine from PDB ID 5LG3 (violet), isoflurane/propofol from PDB ID 4Z90/5MUO (green), ketamine from PDB ID 4F8H (red), bromoethanol from PDB ID 5SXV (blue), mematine from PDB ID 4TWD (pink).

Supplementary Figure S2 Total number of alleles per variant in each gene of interest. On the *x*-axis the respective genes of interest are listed; the *y*-axis reflects the total number of alleles per variant. Each point represents a variant. Red colored variants were excluded from the analysis since they did not pass the outlier test (more than 3 standard deviations away from the mean of total number of alleles). The retained variants (less than 3 standard deviations away from the mean) are colored in gray.

Supplementary Figure S3 Structure-based alignment of the N-terminally truncated ECDs of a GABAA β3 subunit, a GlyR α3 subunit, a nAChR α7 subunit, a 5-HT3R A subunit, a nAChR α3 subunit and a nAChR β4 subunit in this order to visualize the variable regions which are schematically depicted on the topology diagram in Supplementary Figure S4.

Supplementary Figure S4 Topology diagram of cys loop receptor extracellular domain (ECD): The ECD features a modified immunoglobulin-like fold with a twisted beta-sandwich. The topology is as indicated in the image and comprises ten strands (some are broken in many structures) connected as greek key motif. Between conserved blocks, that mainly form the packing core of the domain, multiple variable regions are located (VR1-7) with and without INDELS. Notably, not all variable regions are localized in loop regions, but are in part also interspersed in strands. Strand 9 has a short INDEL. The image depicts the variable regions resulting from a structural comparison of all families, i.e., GABA-A, GlyR, nAChR, and 5HT3-R subunits. Within families, fewer variable regions occur. A representative alignment derived from a structural superposition generated with PDBe Fold is shown in Supplementary Figure S3.

Supplementary Figure S5 3D scatter plots of z-Scale(3) and MSWHIM results for the selected subunit ECD interface binding sites in which the principal and complementary sites were merged to a full pocket amino acid list without the loop F. Both descriptors predict a clear separation between the GABA binding sites and the benzodiazepine binding sites. The diazepam sensitive (DS) sites and the diazepam insensitive (DI) sites, which are formed by different principal subunits, also separate, as indicated on the images. In a next step, pairwise euclidean distances can be used for hierarchical clustering to extract from the descriptor results to clusters (Supplementary Figures S6,S7).

Supplementary Figure S6 z-Scale(3) **(A)** and MSWHIM **(B)** results of the hierarchical clustering for all analyzed ECD interfaces in which the principal and complementary sites were merged to a full pocket amino acid list without loop F.

Supplementary Figure S7 z-Scale(3) **(A)** and MSWHIM **(B)** results of the hierarchical clustering for all analyzed ECD interfaces in which the principal and complementary sites were merged to a full pocket amino acid list, with amino acids on loop F. Note the different results obtained with loop F amino acids, suggesting that smaller and larger pockets need to be analyzed separately.

Supplementary Figure S8
**(A)** Amount of variants across all genes of interest in the dimer onto 7EKT. Ribbon heatmap represents the sum of minor allele counts per 100,000 at each aligned position. **(B)** Variability of variable regions in individual genes. (a) Sum of minor allele numbers per 100,000 in individual variable regions on a logarithmic scale. The ECD variable regions are displayed schematically in Supplementary Figure S4. If not stated otherwise, a variable region is defined to contain insertions or deletions of two or more amino acids. (b) Number of variants divided by the length of the variable region in major isoforms of individual genes.

Supplementary Figure S9 % identity and % similarity for the whole TMD for all pentameric ligand-gated ion channels. The alignment which was used for the calculation is shown in Supplementary Item S4.

Supplementary Figure S10 Summary of molecules investigated in pharmacophore screenings. As described in the methods, all drugs for which positive bioactivity at any of the pLGIC families is reported at DrugCentral were used as a small library which represents an incomplete, but representative snapshot of the chemical space comprising approved drugs with desired or off-target interactions with these receptors, irrespective of subtypes or isoforms. Blue tiles = GABAA receptor targeting; green = 5HT3 targeting; red = nACh receptor targeting; purple = glycine receptor targeting; targeting; gray = multi targeting substances. Note that this gallery reflects strictly the data at Drug Central, while for many of these compounds more promiscuity within the superfamily is known already.

Supplementary Figure S11 Heat map of pharmacophore predicted drug-target interaction for the novel glycine receptor site (2), the five screens into the upper TMD pockets (site 3), the lower TMD interface site (4) and the lipid TM3/TM4 associated site bound with pregnanolone sulfate (5). For a detailed description of the generation of pharmacophores, see the *Methods* section of the manuscript. Hits = number of predicted drug-target interactions for the respective site according to the matched pharmacophore model. The colors represent score values ranging from 0 (light blue) to 1 (dark blue), reflecting the number and quality of overlap of matching features between the respective ligand and the pharmacophore model.

Supplementary Figure S12 Summary of the five screens into upper TMD pockets. Chord diagram of pharmacophore predicted drug-target interactions within the upper TMD binding pocket. For this site most structural templates exist in the pLGIC family (PDB IDs: 5VDH (GlyR- α3 homopentamer), 6HUP (GABAA receptor - α1β3γ2L), 6X3T (GABAA receptor - α1β2γ2) , 6X3V (GABAA receptor - α1β2γ2), 6X3W (GABAA receptor - α1β2γ2), 6X3X (GABAA receptor - α1β2γ2), 7EKT (nAChR - α7 homopentamer). The whole substance library was screened into five merged pharmacophores, indicated by the colored bars. For details regarding the generation of pharmacophores, see *Methods* section. Every substance is connected to the respective pharmacophore models for which a hit is predicted. Note, the number of hits ranges from 18 to 119, showcasing that pharmacophore models based on homologous, but not identical pockets between of the pLGIC family members are sensitive enough to differ between them.

Supplementary Figure S13 Total counts of drugs (*y*-axis) associated with the target families as reflected by DrugCentral only (blue), and with the addition of our pharmacophore screening results (red). The pharmacophore screening reflects only those binding sites for which structural data was available and for which screens were performed with the exception of the screen into the ivermectin site (Supplementary Figure S11).

Supplementary Figure S14
**(A)** Descriptor set results of the hierarchical clustering for the z-Scale(3) and MSWHIM for the GABAARs full pockets of site 3. **(B)** Descriptor set results of the hierarchical clustering for the z-Scale(3) and MSWHIM for the site 3. The numbers on the branching points refer to the comparison diagrams in Figure 4. **(C)** Descriptor set results of the hierarchical clustering for the z-Scale(3) and MSWHIM for the ivermectin binding site. The numbers on the branching points refer to the comparison diagrams in Supplementary Figure S14D. **(D)** Comparison diagram for the ivermectin site in the upper TMD. The left half of the diagram reflects the z- Scale (3) results, and the right half the MSWHIM results.

Supplementary Figure S15 Descriptor set results of the hierarchical clustering for the z-Scale(3) and MSWHIM for the site 4. The numbers on the branching points refer to the comparison diagrams in Figure 6.

Supplementary Figure S16 Descriptor set results of the hierarchical clustering for the z-Scale(3) and MSWHIM for the site 5. The numbers on the branching points refer to the comparison diagrams in Figure 7.

Supplementary Figure S17
**(A)** Multiple views of the 3d scatter plot from Figure 5 with some conformational differences of three different receptor subtypes from the nicotinic acetylcholine receptors (red structures) and a phenobarbital bound site 3 of a GABAA receptor (blue). The numbers in the scatter plots identify the structures used for the RMSD matrix, reflecting representative conformations. Specific examples are displayed in the same style as in Figure 5: Different conformations result in different distances between alpha carbon atoms: A plane through the upper TMD is depicted with all five TM2 segments and two subunits. Distances between alpha carbons are shown to illustrate backbone conformation, where yellow lines are distances between homologous pore forming TM2 residues as an indication for asymmetry, purple lines are distances between pocket forming residues defining the circumference, and green lines the pocket “diagonals.” **(B)** Example of two different conformations of 5HT3A. **(C)** Scatter plots of the site 4 conformation analysis matching Figure 8, with representative RMSD values from the labeled structures. **(D)** Scatter plots of the site 5 conformation analysis matching Figure 8, with representative RMSD values from the labeled structures.

Supplementary Figure S18 Minor allele frequency of variants in TMD subregions. **(A)** Heatmap representing the upper TMD binding site. **(B)** Heatmap representing the extension of the upper TMD binding site with amino acids involved in binding of ivermectin. **(C)** Heatmap representing the steroid binding site. **(D)** Heatmap representing amino acids on TM2 helix oriented with side chains towards the pore. Heatmaps show the sum of minor allele counts per 100,000 in gene and amino acid combinations. Amino acids are numbered according to the reference alignment (Supplementary Item S3). The amino acids correspond to those which are color coded in Supplementary Figure S18 for the upper and lower TMD binding sites, the PS-site.

Supplementary Figure S19 Number of different variants in TMD subregions. **(A)** Heatmap representing the upper TMD binding site. **(B)** Heatmap representing the extension of the upper TMD binding site with amino acids involved in binding of ivermectin. **(C)** Heatmap representing the steroid binding site. **(D)** Heatmap representing amino acids on TM2 helix oriented with side chains towards the pore. Amino acids are numbered according to the reference alignment.

Supplementary Figure S20 An alternative binding mode for the docking of THC into 5OSC showing close proximity to the glycine α3.

Supplementary Figure S21 Tentative THC binding sites and candidate positions for mutational analysis. Ribbon rendering of two subunits’ TMD domains, helices that contribute to sites 3, 4, or 5 are labeled. Colour codes match Figure 9. Site 3 in the upper TMD: Representative docking pose of THC (green) in superposition with PNU-120596 (light pink, 7EKT). Green ribbon segments on principal TM2, TM3 and on complementary TM1 form ligand contacts. The segment numbering matches the table below the image, amino acid numbering as in (PDBID - 5OSB) for all sites. Site 4: Representative docking pose of THC (cyan) in superposition with THDOC (light pink, 5OSB). Cyan ribbon segments on principal TM3 and complementary TM4 form ligand contacts. Site 5: Representative docking pose of THC (gold) in (partial) superposition with preganolone sulfate (PS, light pink, 5OSC). The best scoring docking poses overlap only to a small extent with PS. Golden ribbon segments on TM3 and TM4 form ligand contacts. The bright yellow segment with the * marks the position which has been mutated in the GlyR α3 subunit, see Figure 9.

Supplementary Item S1 This is an Excel file with a comprehensive list of PDB files of pLGICs, those used for this study and many more.

Supplementary Item S2 List of drugs which were used for the structure-based pharmacophore screening.

Supplementary Item S3 FASTA formatted file containing the aligned sequences used in variant analysis.

Supplementary Item S4 This file contains a color coded alignment reflecting the amino acids used for the TMD binding sites.

Supplementary Table S1 Gene names and their corresponding uniprot IDs.

Supplementary Table S2 Structures used for the pharmacophore screens.

## Abbreviations

AEs, adverse events; Bz, benzodiazepine; CBD, cannabidiol; CNS, central nervous system; ACh, acetylcholine; nAChR, nicotinic acetylcholine receptor; ECD, extracellular domain; EMA, European medicines agency; FDA, US Food and drug administration; GABA, gamma- aminobutyric acid; GABA_A_R, GABA_A_ receptor; ICD, intracellular Domain; INDEL, insertion/deletion; PDB, protein database; THC, Δ^9^-tetrahydrocannabinol (= dronabinol); TMD, transmembrane domain; pLGIC, pentameric ligand gated ion channel; ZAC, zinc-activated ligand gated ion channel; 5-HT_3_, 5-hydroxytryptamine type 3 receptors.
