# Supplementary material for: Molecular Mingling: Multimodal Predictions of Ligand Promiscuity in Pentameric Ligand-Gated Ion Channels
Source: Front Mol Biosci. 2022 May 9;9:860246. doi: 10.3389/fmolb.2022.860246 (PMC9124788; doi:10.3389/fmolb.2022.860246)
Supplement: Supplementary file 1 [file DataSheet1.PDF]

Supplementary Material

1 Supplementary Data

Supplementary Item 1

Results/PDB_structures_inventory.xlsx.
This is an Excel file with a comprehensive list of PDB files of pLGICs, those used for this study and many more.

Supplementary Item 2

Drugcentral_drugs.xlsx

List of drugs which were used for the structure-based pharmacophore screening.

Supplementary Item 3

Variants_alignment.fasta

FASTA formatted file containing the aligned sequences used in variant analysis.

Supplementary Item 4

TMD_alignment.xlsx
This file contains a color coded alignment reflecting the amino acids used for the TMD binding sites.

2 Supplementary Figures and Tables

2.1 Supplementary Figures

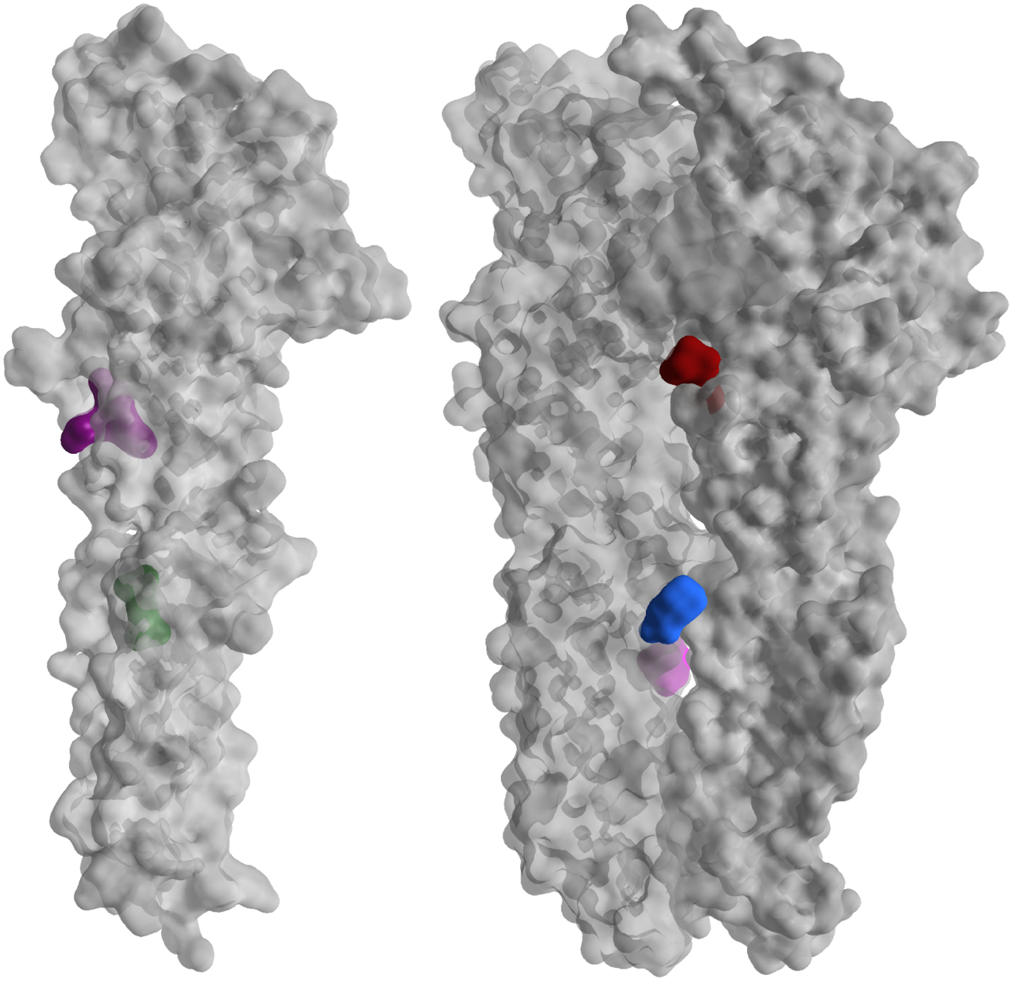

Supplementary Figure 1. Superposed structures and their pockets derived from remote homologues. Chlorpromazine from PDB ID 5LG3 (violet), isoflurane/
propofol from PDB ID 4Z90/5MUO (green), ketamine from PDB ID 4F8H (red), bromoethanol from PDB ID 5SXV (blue), mematine from PDB ID 4TWD (pink).

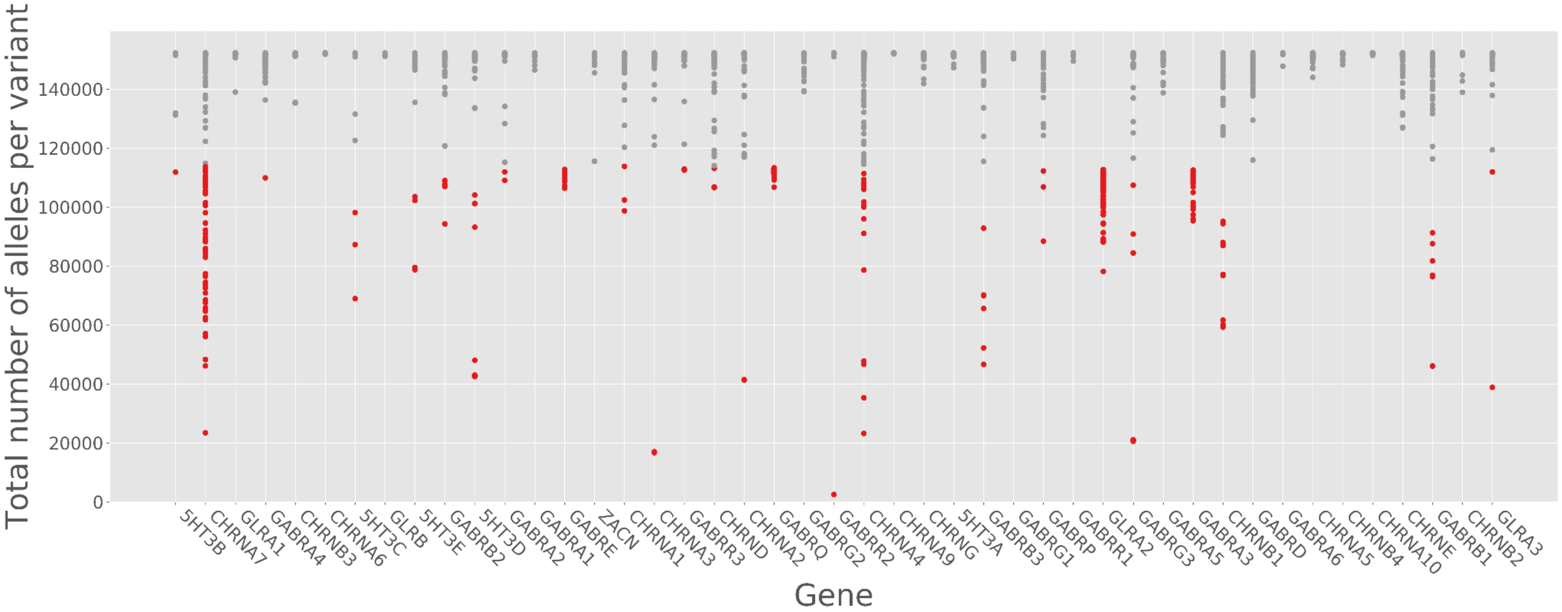

Supplementary Figure 2. Total number of alleles per variant in each gene of interest. On the x-axis the respective genes of interest are listed; the y-axis reflects the total number of alleles per variant. Each point represents a variant. Red colored variants were excluded from the analysis since they did not pass the outlier test (more than 3 standard deviations away from the mean of total number of alleles). The retained variants (less than 3 standard deviations away from the mean) are colored in gray.

Alignment (ECD part) obtained from PDBeFold for the chains as given in the alignment. Secondary structures annotated in a representative sequence:
yellow: strands as identified by PDBeFold purple: N-terminal helix
strand numbers and variable region annotation above the alignment match the topology diagram.

```
                VR1            VR2
                   HHHHHHHHHHH      ............. ....1111111111111.....2222
PDB_4cof_B      ---SFVKETVDKLL---K--GYDIRLRPDF-GGPPVCVGMNIDIASIDMVSEVNMDYTLT
PDB_5vdh_B      -APMSPSDFLDKLMGRTS--GYDARIR-PNFKGPPVNVTCNIFINSFGSIAETTMDYRVN
PDB_7ekt_B      --GEFQRKLYKELV---K--NYNPLERPVANDSQPLTVYFSLSLLQIMDVDEKNQVLTTN
PDB_6hin_B      ---PALLRLSDHLL---A--NYKKGVRPVRDWRKPTTVSIDVIMYAILNVDEKNQVLTTY
PDB_6pv7_A      --SEAEHRLFERLF---E--DYNEIIRPVANVSDPVIIHFEVSMSQLVKVDEVNQIMETN
PDB_6pv7_B      RVANAEEKLMDDLL---NKTRYNNLIRPATSSSQLISIKLQLSLAQLISVNEREQIMTTN
                          . *.        *.    *          :  .: :  :  :  *
                22222222....   VR3       3333 VR4  .......444..5555   VR5
PDB_4cof_B      MYFQQYWRDKRLAYSGI---P--LN-LTLDNRV--ADQLWVPDTYFLNDKKS--F--VHG
PDB_5vdh_B      IFLRQKWNDPRLAYSEY---P--DDSLDLD--PSMLDSIWKPDLFFANEKGA--N--F--
PDB_7ekt_B      IWLQMSWTDHYLQWNVSE-YPGVK--TVRF--P--DGQIWKPDILLYNSADERFDA-T--
PDB_6hin_B      IWYRQYWTDEFLQWTPED-FDNVT--KLSI--P--TDSIWVPDILINEF-VD--VGKSP-
PDB_6pv7_A      LWLKQIWNDYKLKWNPSDYGGA-E--FMRV--P--AQKIWKPDIVLYNNAVGDFQ--V--
PDB_6pv7_B      VWLKQEWTDYRLTWNSSRYEGV-N--ILRI--P--AKRIWLPDIVLYNNADGTYE--V--
                :: :  * *  * :.                        :* **  :  :
                 ..VR5      5555....6666..6666666............777777777.....
PDB_4cof_B      VTVKN-----R-MIRLHPDGTVLYGLRITTTAACMMDLRRYPLDEQNCTLEIESYGYTTD
PDB_5vdh_B      ---HEVTTDNK-LLRIFKNGNVLYSIRLTLTLSCPMDLKNFPMDVQTCIMQLESFGYTMN
PDB_7ekt_B      ---FH-----T-NVLVNSSGHCQYLPPGIFKSSCYIDVRWFPFDVQHCKLKFGSWSYGGW
PDB_6hin_B      ---N------IPYVYVHHRGEVQNYKPLQLVTACSLDIYNFPFDVQNCSLTFTSWLHTIQ
PDB_6pv7_A      ---DD----KT-KALLKYTGEVTWIPPAIFKSSCKIDVTYFPFDYQNCTMKFGSWSYDKA
PDB_6pv7_B      ---SV----YT-NLIVRSNGSVLWLPPAIYKSACKIEVKYFPFDQQNCTLKFRSWTYDHT
                               :   *            :*  :::  :*:*  *  *  :  :  *:  :
                .88888..     VR6              .9999I999999999    VR7  .000000000000
PDB_4cof_B      DIEFYWRGG-DKA--VTGVER---I-ELP-QFSIV-EHRLVSRNV--VFA--TGAYPRLSLSFRLKR-NI
PDB_5vdh_B      DLIFEWQDEAPV----QV--AE-GL-T-LPQFLLKEEKDLRYCTK--HYN--TGKFTCIEVRFHLERQM-
PDB_7ekt_B      SLDLQMQE--A-----DI--SG-YIPN-G-EWDLV-GIPGKRSER--FYECCKEPYPDVTFTVTMRRRT-
PDB_6hin_B      DINITLWRS-PEEVRSDK--SI-FI-NQG-EWELL-EVFPQFKEF--SID-ISNSYAEMKFYVIIRRRP-
PDB_6pv7_A      KIDLVLIG--SS---MNL--KDYWE-S-G-EWAII-KAPGYKHDI--KYNCCEEIYPDITYSLYIRRLP-
PDB_6pv7_B      EIDMVLMT--PT---ASM--DDFTP-S-G-EWDIV-ALPGRRTVNPQD-----PSYVDVTYDFIIKRKP-
                .: :                          :: :                      :  :   . :.*
```

Supplementary Figure 3. Structure-based alignment of the N-terminally truncated ECDs of a GABAA β3 subunit, a GlyR α3 subunit, a nAChR α7 subunit, a 5-HT3R A subunit, a nAChR α3 subunit and a nAChR β4 subunit in this order to visualize the variable regions which are schematically depicted on the topology diagram in Supplementary Figure 4.

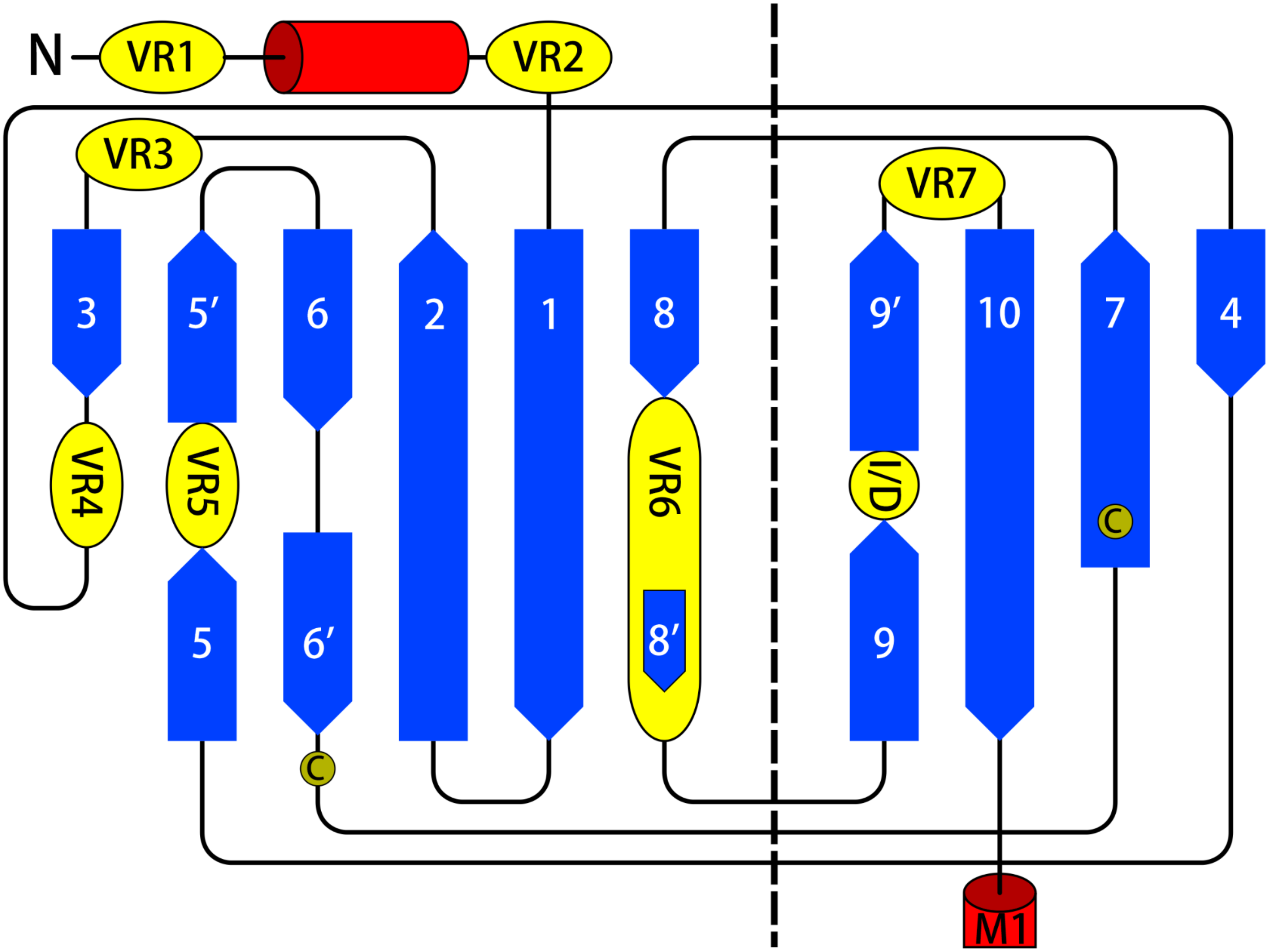

Supplementary Figure 4. Topology diagram of cys loop receptor extracellular domain (ECD): The ECD features a modified immunoglobulin-like fold with a twisted beta-sandwich. The topology is as indicated in the image and comprises ten strands (some are broken in many structures) connected as greek key motif. Between conserved blocks, that mainly form the packing core of the domain, multiple variable regions are located (VR1-7) with and without INDELS. Notably, not all variable regions are localized in loop regions, but are in part also interspersed in strands. Strand 9 has a short INDEL. The image depicts the variable regions resulting from a structural comparison of all families, i.e. GABA-A, GlyR, nAChR and 5HT3-R subunits. Within families, fewer variable regions occur. A representative alignment derived from a structural superposition generated with PDBe Fold is shown in Supplementary Figure 3.

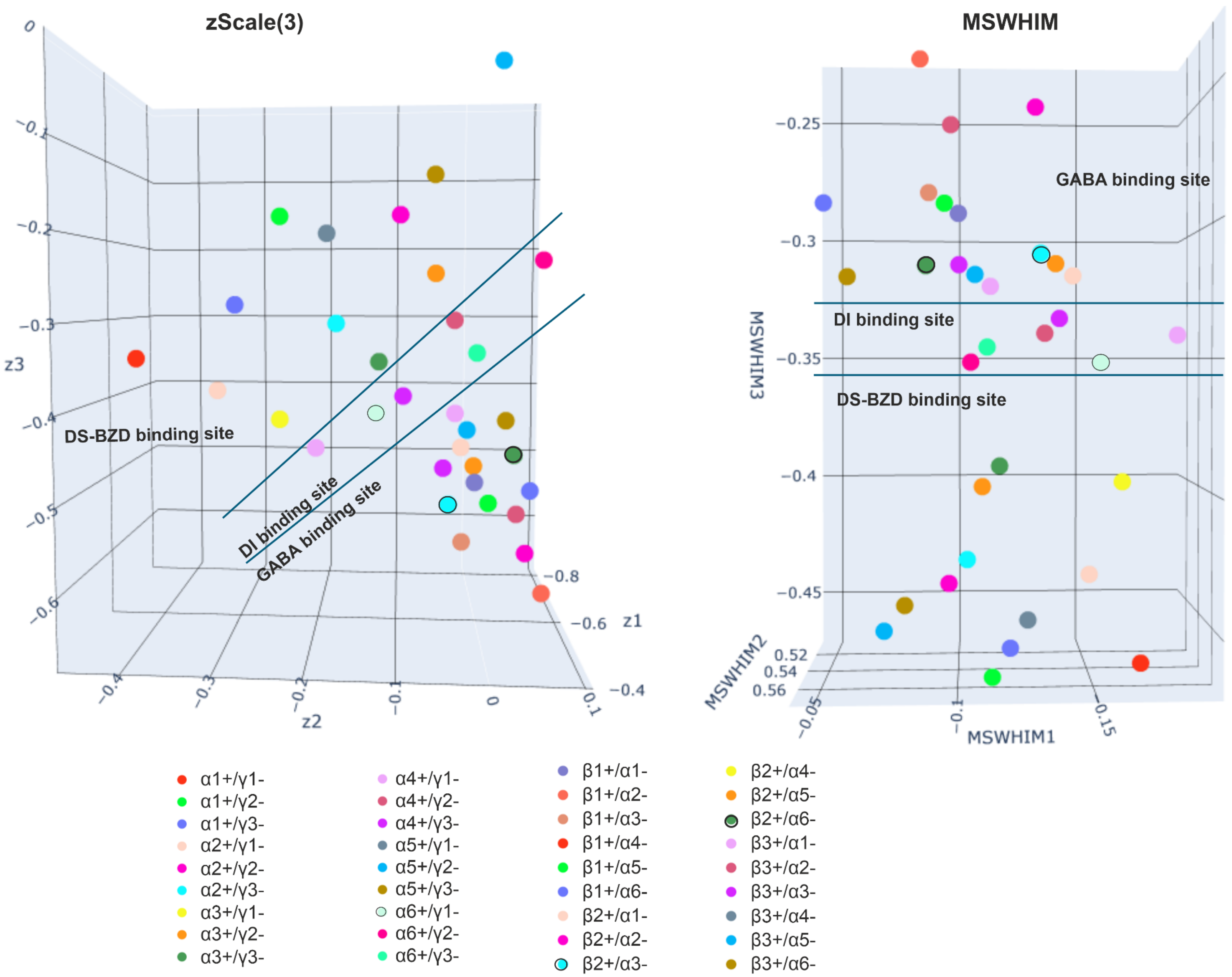

Supplementary Figure 5. 3D scatter plots of z-Scale(3) and MSWHIM results for the selected subunit ECD interface binding sites in which the principal and complementary sites were merged to a full pocket amino acid list without the loop F. Both descriptors predict a clear separation between the GABA binding sites and the benzodiazepine binding sites. The diazepam sensitive (DS) sites and the diazepam insensitive (DI) sites, which are formed by different principal subunits, also separate, as indicated on the images. In a next step, pairwise euclidean distances can be used for hierarchical clustering to extract from the descriptor results to clusters, (Supplementary Figures 6 and 7).

A

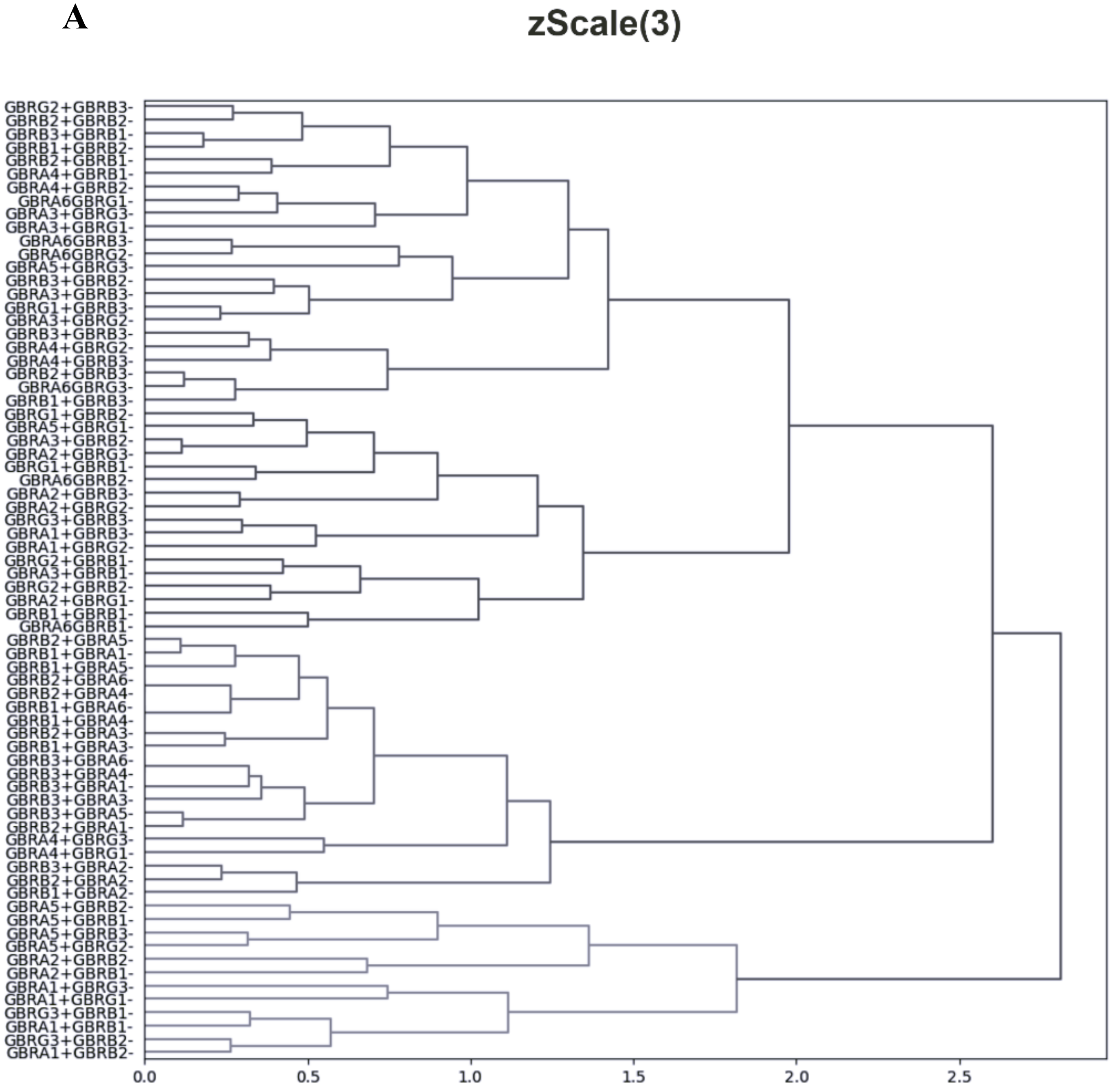
zScale(3)
GBRG2+GBRB3-
GBRB2+GBRB2-
GBRB3+GBRB1-
GBRB1+GBRB2-
GBRB2+GBRB1-
GBRA4+GBRB1-
GBRA4+GBRB2-
GBRA6GBRG1-
GBRA3+GBRG3-
GBRA3+GBRG1-
GBRA6GBRB3-
GBRA6GBRG2-
GBRA5+GBRG3-
GBRB3+GBRB2-
GBRA3+GBRB3-
GBRG1+GBRB3-
GBRA3+GBRG2-
GBRB3+GBRB3-
GBRA4+GBRG2-
GBRA4+GBRB3-
GBRB2+GBRB3-
GBRA6GBRG3-
GBRB1+GBRB3-
GBRG1+GBRB2-
GBRA5+GBRG1-
GBRA3+GBRB2-
GBRA2+GBRG3-
GBRG1+GBRB1-
GBRA6GBRB2-
GBRA2+GBRB3-
GBRA2+GBRG2-
GBRG3+GBRB3-
GBRA1+GBRB3-
GBRA1+GBRG2-
GBRG2+GBRB1-
GBRA3+GBRB1-
GBRG2+GBRB2-
GBRA2+GBRG1-
GBRB1+GBRB1-
GBRA6GBRB1-
GBRB2+GBRA5-
GBRB1+GBRA1-
GBRB1+GBRA5-
GBRB2+GBRA6-
GBRB2+GBRA4-
GBRB1+GBRA6-
GBRB1+GBRA4-
GBRB2+GBRA3-
GBRB1+GBRA3-
GBRB3+GBRA6-
GBRB3+GBRA4-
GBRB3+GBRA1-
GBRB3+GBRA3-
GBRB3+GBRA5-
GBRB2+GBRA1-
GBRA4+GBRG3-
GBRA4+GBRG1-
GBRB3+GBRA2-
GBRB2+GBRA2-
GBRB1+GBRA2-
GBRA5+GBRB2-
GBRA5+GBRB1-
GBRA5+GBRB3-
GBRA5+GBRG2-
GBRA2+GBRB2-
GBRA2+GBRB1-
GBRA1+GBRG3-
GBRA1+GBRG1-
GBRG3+GBRB1-
GBRA1+GBRB1-
GBRG3+GBRB2-
GBRA1+GBRB2-
0.0
0.5
1.0
1.5
2.0
2.5

B

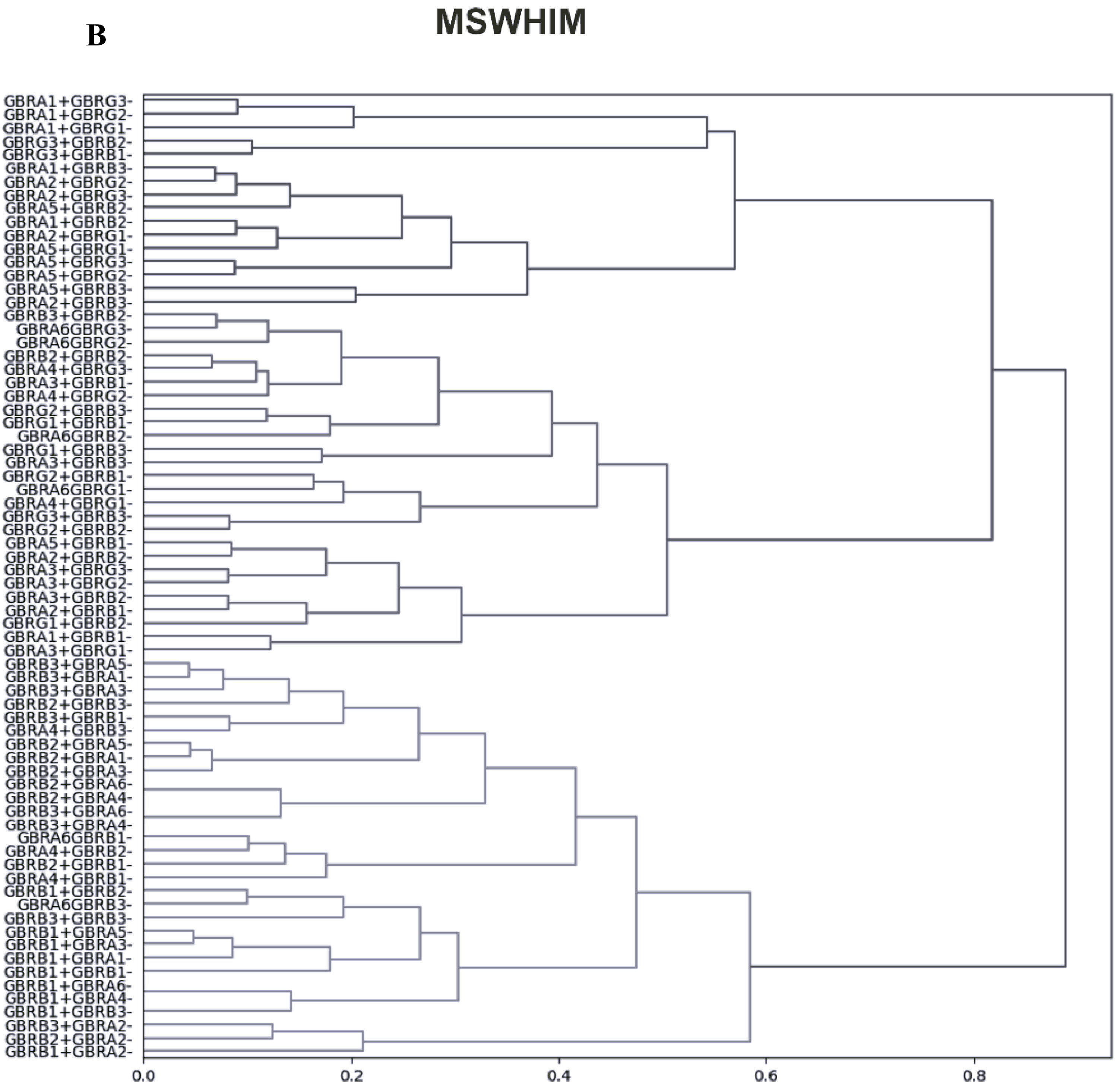

Supplementary Figure 6. z-Scale(3) (**A**) and MSWHIM (**B**) results of the hierarchical clustering for all analyzed ECD interfaces in which the principal and complementary sites were merged to a full pocket amino acid list without loop F.

A

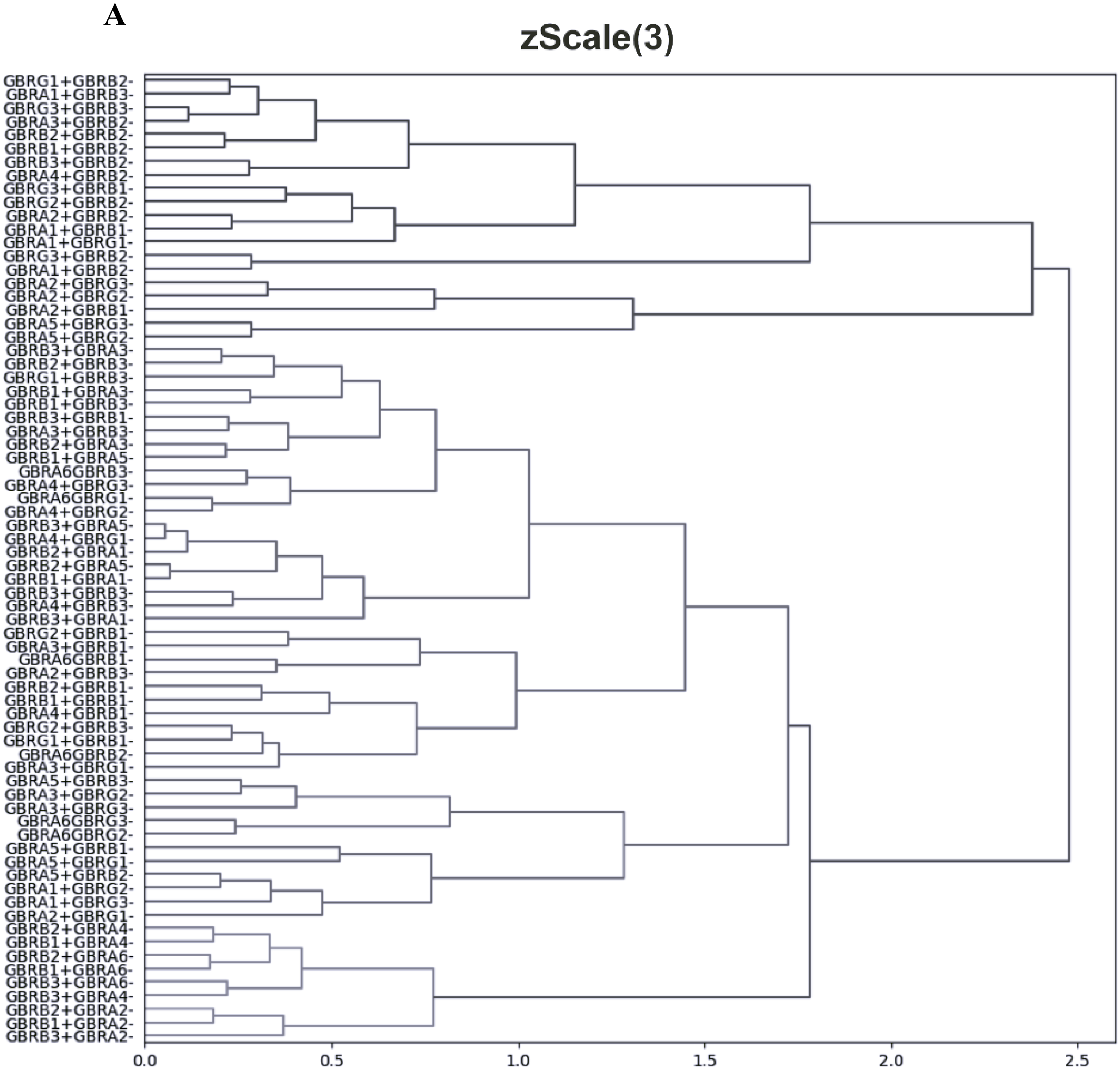
zScale(3)
GBRG1+GBRB2-
GBRA1+GBRB3-
GBRG3+GBRB3-
GBRA3+GBRB2-
GBRB2+GBRB2-
GBRB1+GBRB2-
GBRB3+GBRB2-
GBRA4+GBRB2-
GBRG3+GBRB1-
GBRG2+GBRB2-
GBRA2+GBRB2-
GBRA1+GBRB1-
GBRA1+GBRG1-
GBRG3+GBRB2-
GBRA1+GBRB2-
GBRA2+GBRG3-
GBRA2+GBRG2-
GBRA2+GBRB1-
GBRA5+GBRG3-
GBRA5+GBRG2-
GBRB3+GBRA3-
GBRB2+GBRB3-
GBRG1+GBRB3-
GBRB1+GBRA3-
GBRB1+GBRB3-
GBRB3+GBRB1-
GBRA3+GBRB3-
GBRB2+GBRA3-
GBRB1+GBRA5-
GBRA6GBRB3-
GBRA4+GBRG3-
GBRA6GBRG1-
GBRA4+GBRG2-
GBRB3+GBRA5-
GBRA4+GBRG1-
GBRB2+GBRA1-
GBRB2+GBRA5-
GBRB1+GBRA1-
GBRB3+GBRB3-
GBRA4+GBRB3-
GBRB3+GBRA1-
GBRG2+GBRB1-
GBRA3+GBRB1-
GBRA6GBRB1-
GBRA2+GBRB3-
GBRB2+GBRB1-
GBRB1+GBRB1-
GBRA4+GBRB1-
GBRG2+GBRB3-
GBRG1+GBRB1-
GBRA6GBRB2-
GBRA3+GBRG1-
GBRA5+GBRB3-
GBRA3+GBRG2-
GBRA3+GBRG3-
GBRA6GBRG3-
GBRA6GBRG2-
GBRA5+GBRB1-
GBRA5+GBRG1-
GBRA5+GBRB2-
GBRA1+GBRG2-
GBRA1+GBRG3-
GBRA2+GBRG1-
GBRB2+GBRA4-
GBRB1+GBRA4-
GBRB2+GBRA6-
GBRB1+GBRA6-
GBRB3+GBRA6-
GBRB3+GBRA4-
GBRB2+GBRA2-
GBRB1+GBRA2-
GBRB3+GBRA2-
0.0
0.5
1.0
1.5
2.0
2.5

B

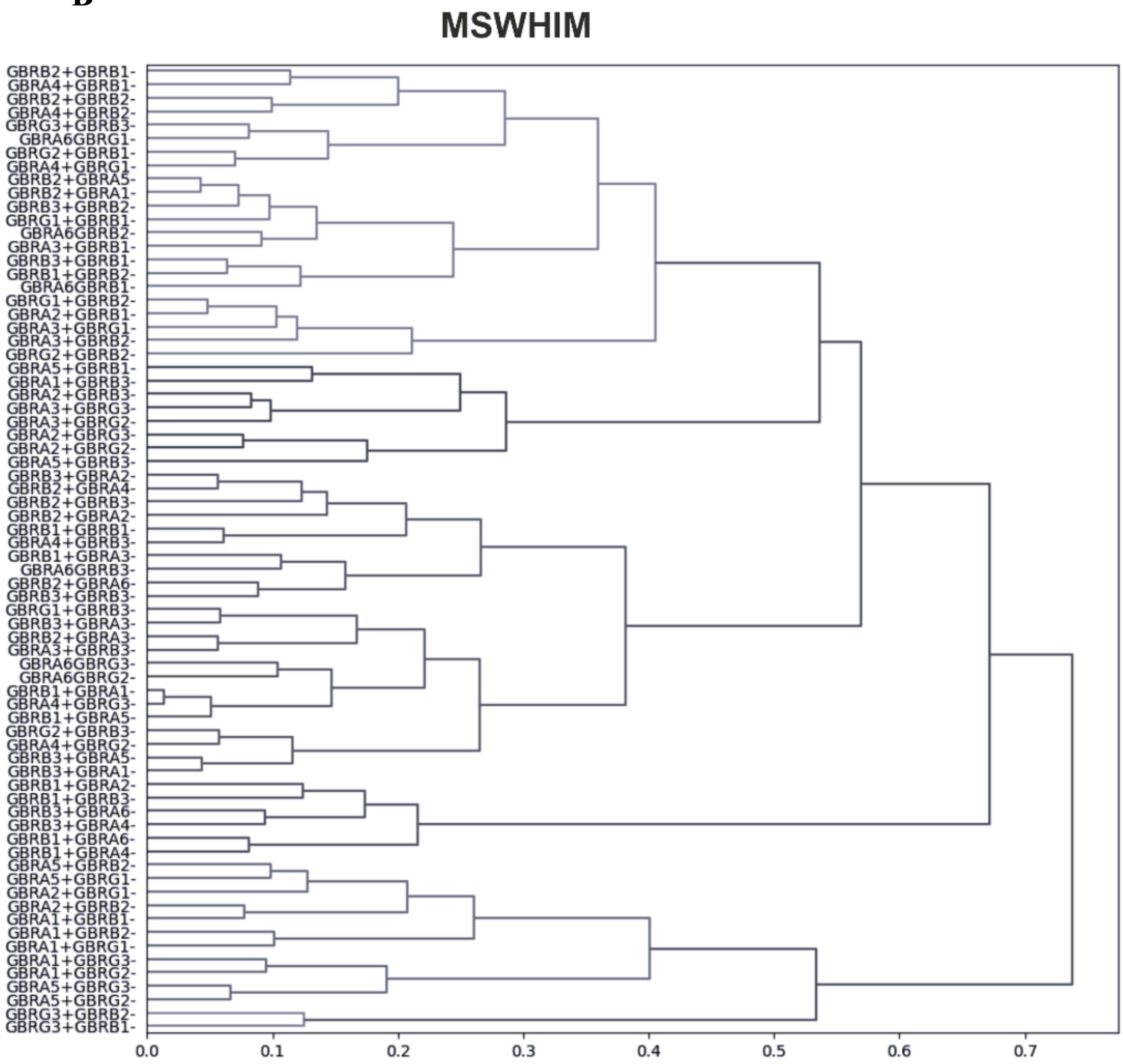

Supplementary Figure 7. z-Scale(3) (**A**) and MSWHIM (**B**) results of the hierarchical clustering for all analyzed ECD interfaces in which the principal and complementary sites were merged to a full pocket amino acid list, with amino acids on loop F. Note the different results obtained with loop F amino acids, suggesting that smaller and larger pockets need to be analyzed separately.

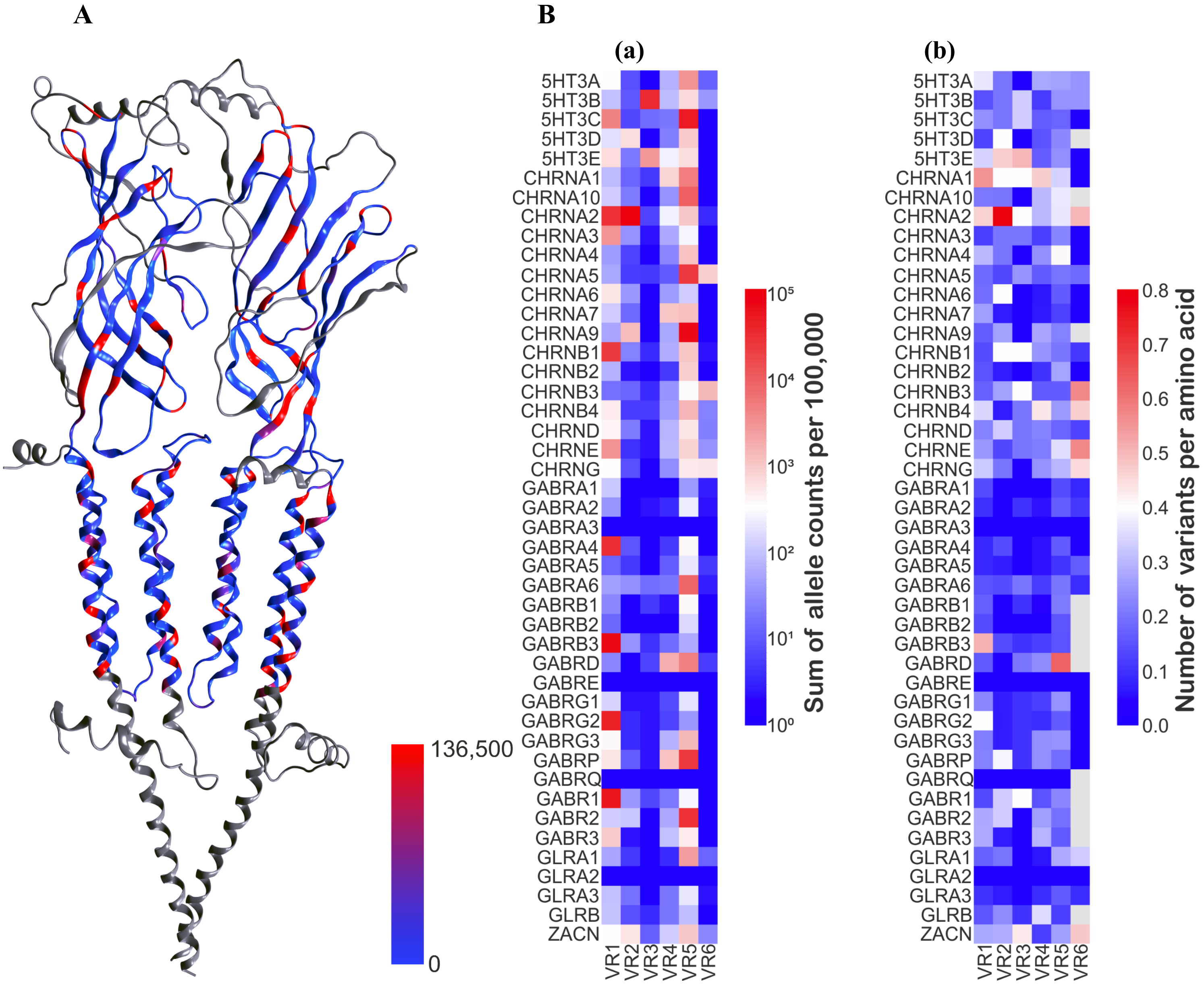

Supplementary Figure 8. A Amount of variants across all genes of interest in the dimer onto 7EKT. Ribbon heatmap represents the sum of minor allele counts per 100,000 at each aligned position. **B** Variability of variable regions in individual genes. (a) Sum of minor allele numbers per 100,000 in individual variable regions on a logarithmic scale. The ECD variable regions are displayed schematically in Supplementary Figure 4. If not stated otherwise, a variable region is defined to contain insertions or deletions of 2 or more amino acids. (b) Number of variants divided by the length of the variable region in major isoforms of individual genes.

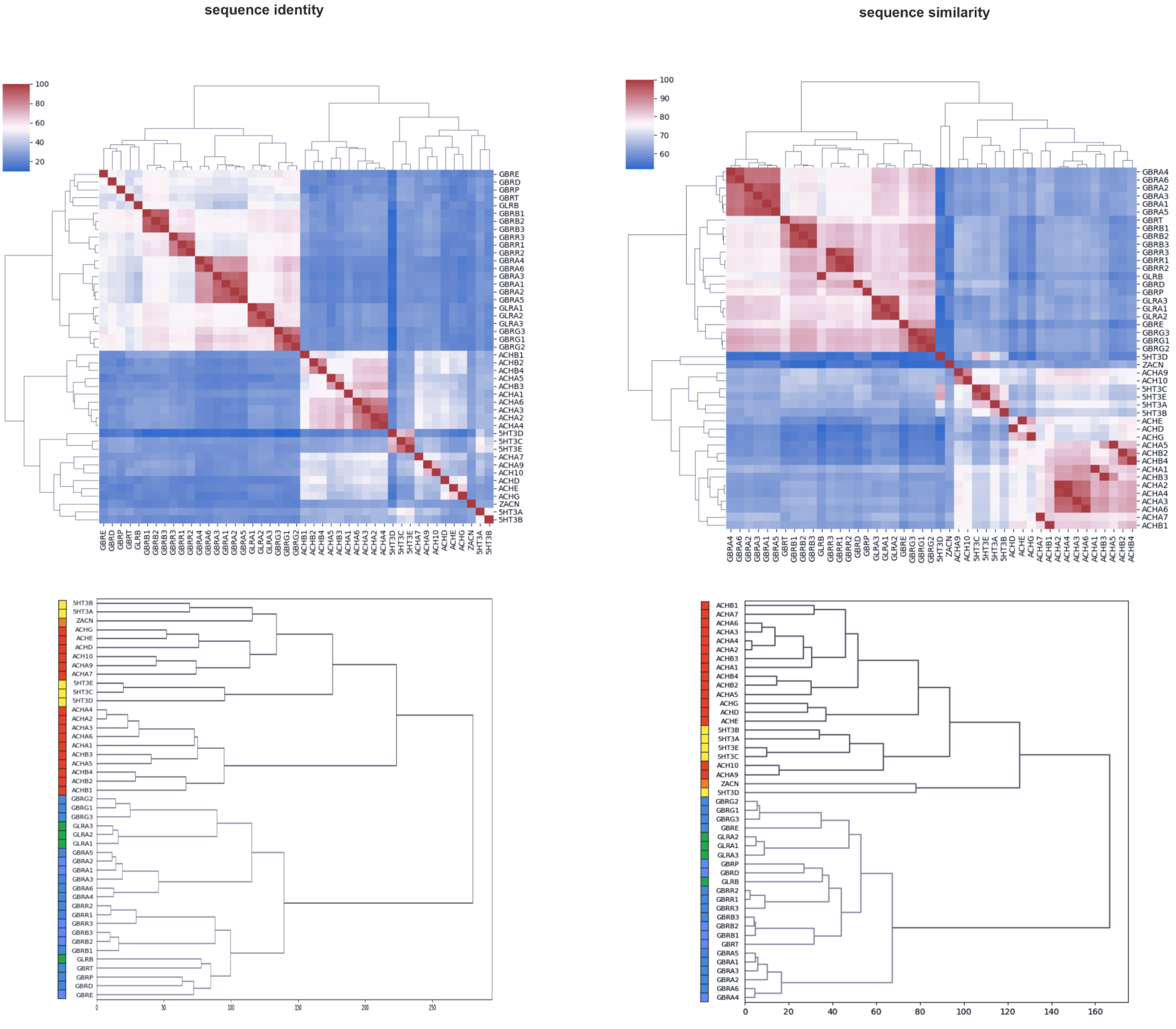

Supplementary Figure 9. % identity and % similarity for the whole TMD for all pentameric ligand-gated ion channels. The alignment which was used for the calculation is shown in Supplementary item 4.

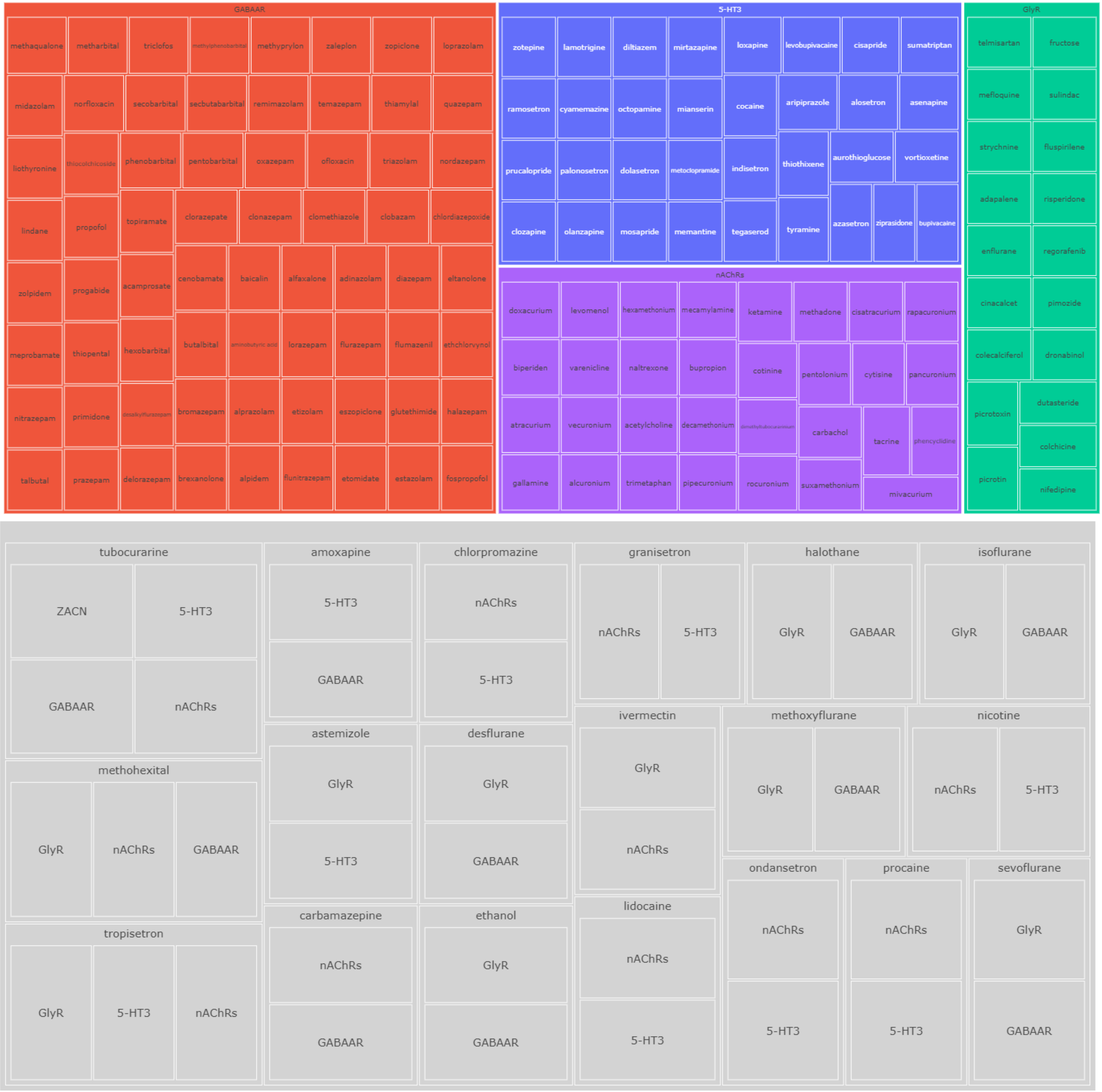

GABAAR
methaqualone
metharbital
triclofos
methyprylon
zaleplon
zopiclone
loprazolam
midazolam
norfloxacin
secobarbital
secbutabarbital
remimazolam
temazepam
thiamylal
quazepam
liothyronine
thiocolchicoside
phenobarbital
pentobarbital
oxazepam
ofloxacin
triazolam
nordazepam
lindane
propofol
topiramate
clorazepate
clonazepam
clomethiazole
clobazam
chlordiazepoxide
zolpidem
progabide
acamprosate
cenobamate
baicalin
alfaxalone
adinazolam
diazepam
eltanolone
meprobamate
thiopental
hexobarbital
butalbital
lorazepam
flurazepam
flumazenil
ethchlorvynol
nitrazepam
primidone
bromazepam
alprazolam
etizolam
eszopiclone
glutethimide
halazepam
talbutal
prazepam
delorazepam
brexanolone
alpidem
flunitrazepam
etomidate
estazolam
fospropofol
5-HT3
zotepine
lamotrigine
diltiazem
mirtazapine
loxapine
levobupivacaine
cisapride
sumatriptan
ramosetron
cyamemazine
octopamine
mianserin
cocaine
aripiprazole
alosetron
asenapine
prucalopride
palonosetron
dolasetron
metoclopramide
indisetron
thiothixene
aurothioglucose
vortioxetine
clozapine
olanzapine
mosapride
memantine
tegaserod
tyramine
azasetron
ziprasidone
bupivacaine
nAChRs
doxacurium
levomenol
hexamethonium
mecamylamine
ketamine
methadone
cisatracurium
rapacuronium
biperiden
varenicline
naltrexone
bupropion
cotinine
pentolonium
cytisine
pancuronium
atracurium
vecuronium
acetylcholine
decamethonium
carbachol
tacrine
phencyclidine
gallamine
alcuronium
trimetaphan
pipecuronium
rocuronium
suxamethonium
mivacurium
GlyR
telmisartan
fructose
mefloquine
sulindac
strychnine
fluspirilene
adapalene
risperidone
enflurane
regorafenib
cinacalcet
pimozide
colecalciferol
dronabinol
picrotoxin
dutasteride
colchicine
picrotin
nifedipine
tubocurarine
ZACN
5-HT3
GABAAR
nAChRs
amoxapine
5-HT3
GABAAR
chlorpromazine
nAChRs
5-HT3
granisetron
nAChRs
5-HT3
halothane
GlyR
GABAAR
isoflurane
GlyR
GABAAR
astemizole
GlyR
5-HT3
desflurane
GlyR
GABAAR
ivermectin
GlyR
nAChRs
methoxyflurane
GlyR
GABAAR
nicotine
nAChRs
5-HT3
methohexital
GlyR
nAChRs
GABAAR
ondansetron
nAChRs
5-HT3
procaine
nAChRs
5-HT3
sevoflurane
GlyR
GABAAR
lidocaine
nAChRs
5-HT3
carbamazepine
nAChRs
GABAAR
ethanol
GlyR
GABAAR
tropisetron
GlyR
5-HT3
nAChRs

Supplementary Figure 10. Summary of molecules investigated in pharmacophore screenings. As described in the methods, all drugs for which positive bioactivity at any of the pLGIC families is reported at DrugCentral were used as a small library which represents an incomplete, but representative snapshot of the chemical space comprising approved drugs with desired or off-target interactions with these receptors, irrespective of subtypes or isoforms. Blue tiles = GABAA receptor targeting; green = $5HT_3$ targeting; red = nACh receptor targeting; purple = glycine receptor targeting; targeting; gray = multi targeting substances. Note that this gallery reflects strictly the data at Drug Central, while for many of these compounds more promiscuity within the superfamily is known already

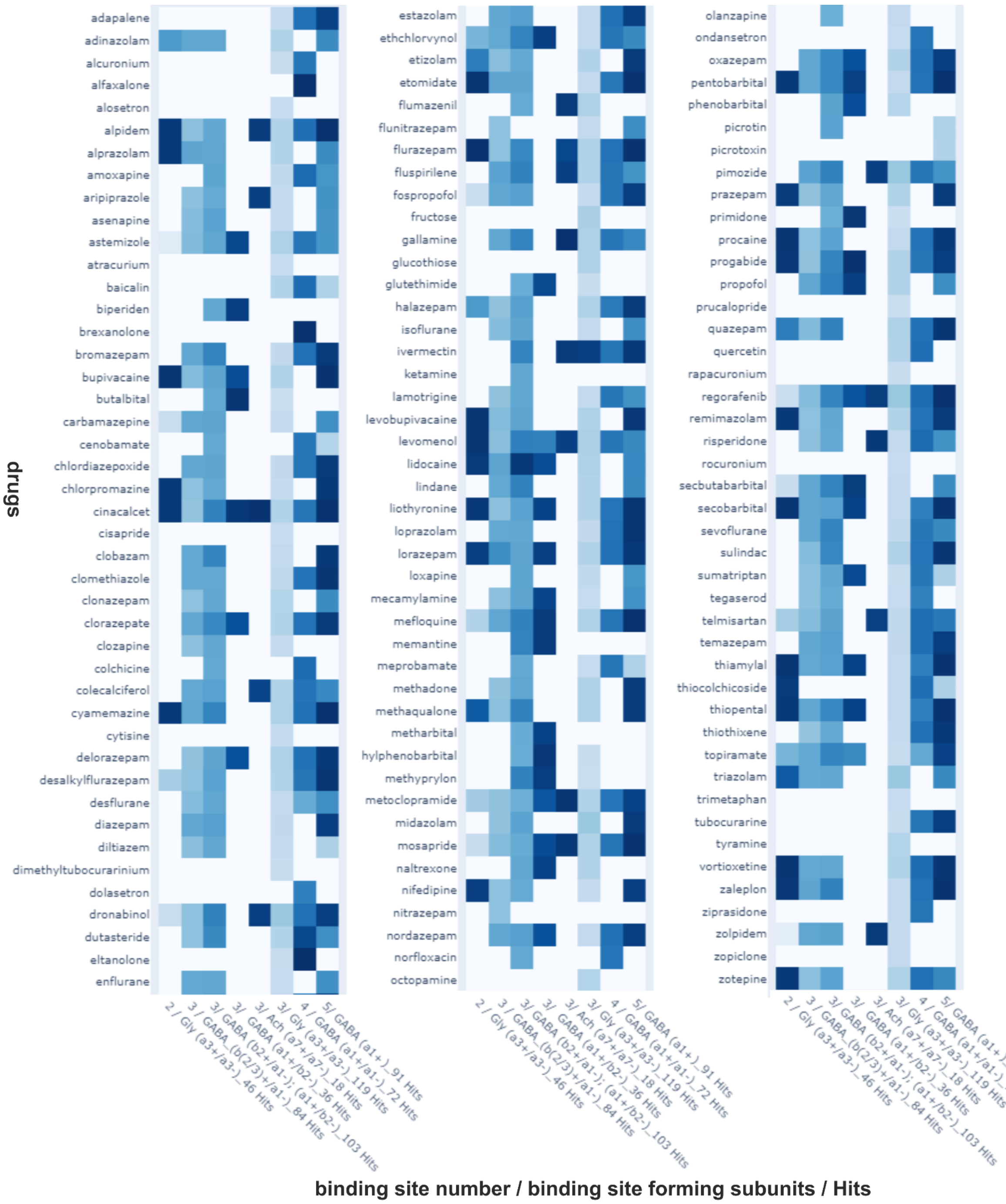

Supplementary Figure 11. Heat map of pharmacophore predicted drug-target interaction for the novel glycine receptor site (2), the five screens into the upper TMD pockets (site 3), the lower TMD interface site (4) and the lipid M3/M4 associated site bound with pregnanolone sulfate (5). For a detailed description of the generation of pharmacophores, see the methods section of the manuscript. Hits = number of predicted drug-target interactions for the respective site according to the matched pharmacophore model. The colors represent score values ranging from 0 (light

blue) to 1 (dark blue), reflecting the number and quality of overlap of matching features between the respective ligand and the pharmacophore model.

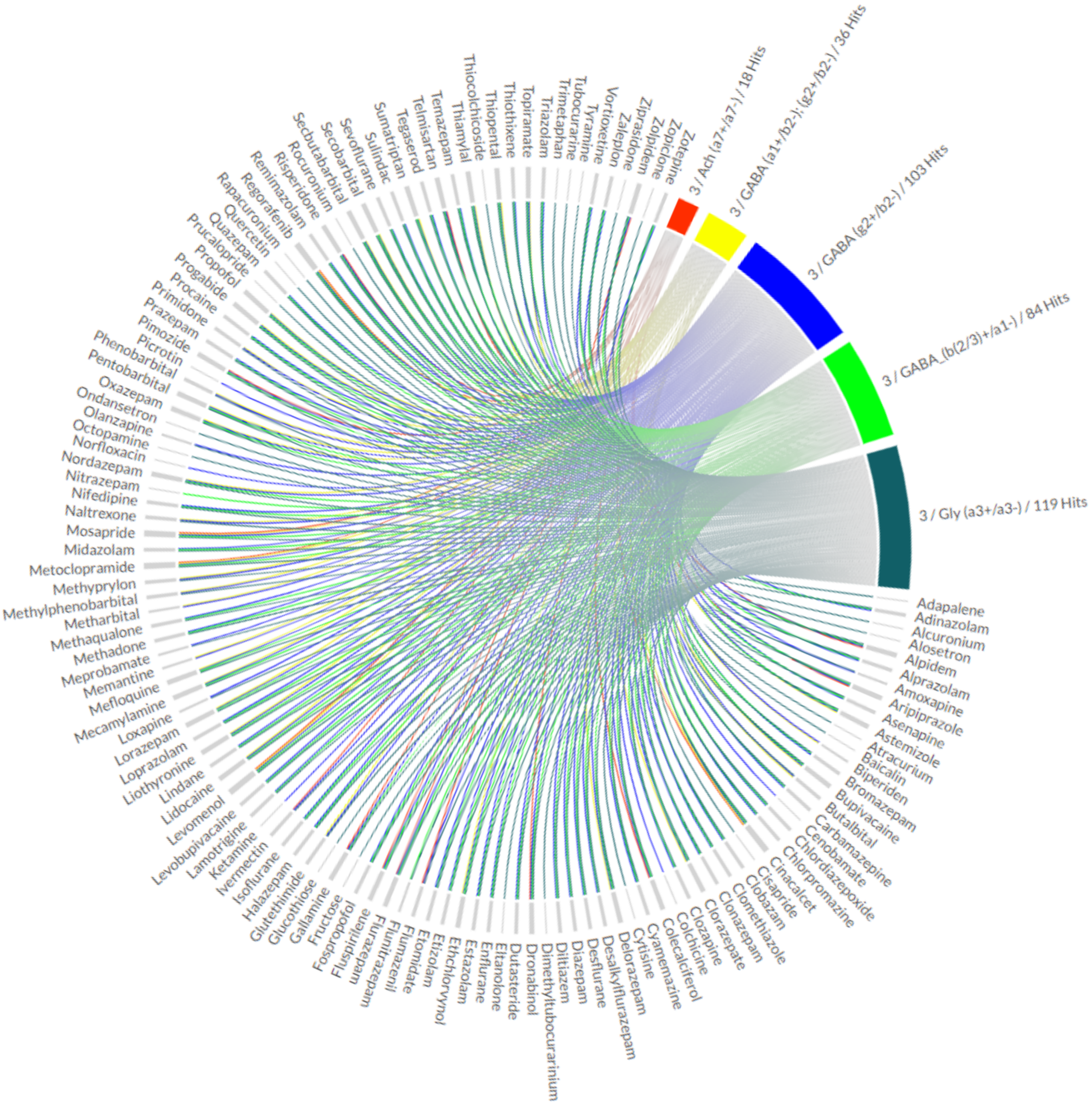

Supplementary Figure 12. Summary of the five screens into upper TMD pockets. Chord diagram of pharmacophore predicted drug-target interactions within the upper TMD binding pocket. For this site most structural templates exist in the pLGIC family (PDB IDs: 5VDH (GlyR - α3 homopentamer), 6HUP ($GABA_A$ receptor - α1β3γ2L), 6X3T ($GABA_A$ receptor - α1β2γ2) ,

6X3V ($GABA_A$ receptor - α1β2γ2), 6X3W ($GABA_A$ receptor - α1β2γ2), 6X3X ($GABA_A$ receptor - α1β2γ2), 7EKT (nAChR - α7 homopentamer). The whole substance library was screened into five merged pharmacophores, indicated by the colored bars. For details regarding the generation of pharmacophores, see methods section. Every substance is connected to the respective pharmacophore models for which a hit is predicted. Note, the number of hits ranges from 18 to 119, showcasing that pharmacophore models based on homologous, but not identical pockets between of the pLGIC family members are sensitive enough to differ between them.

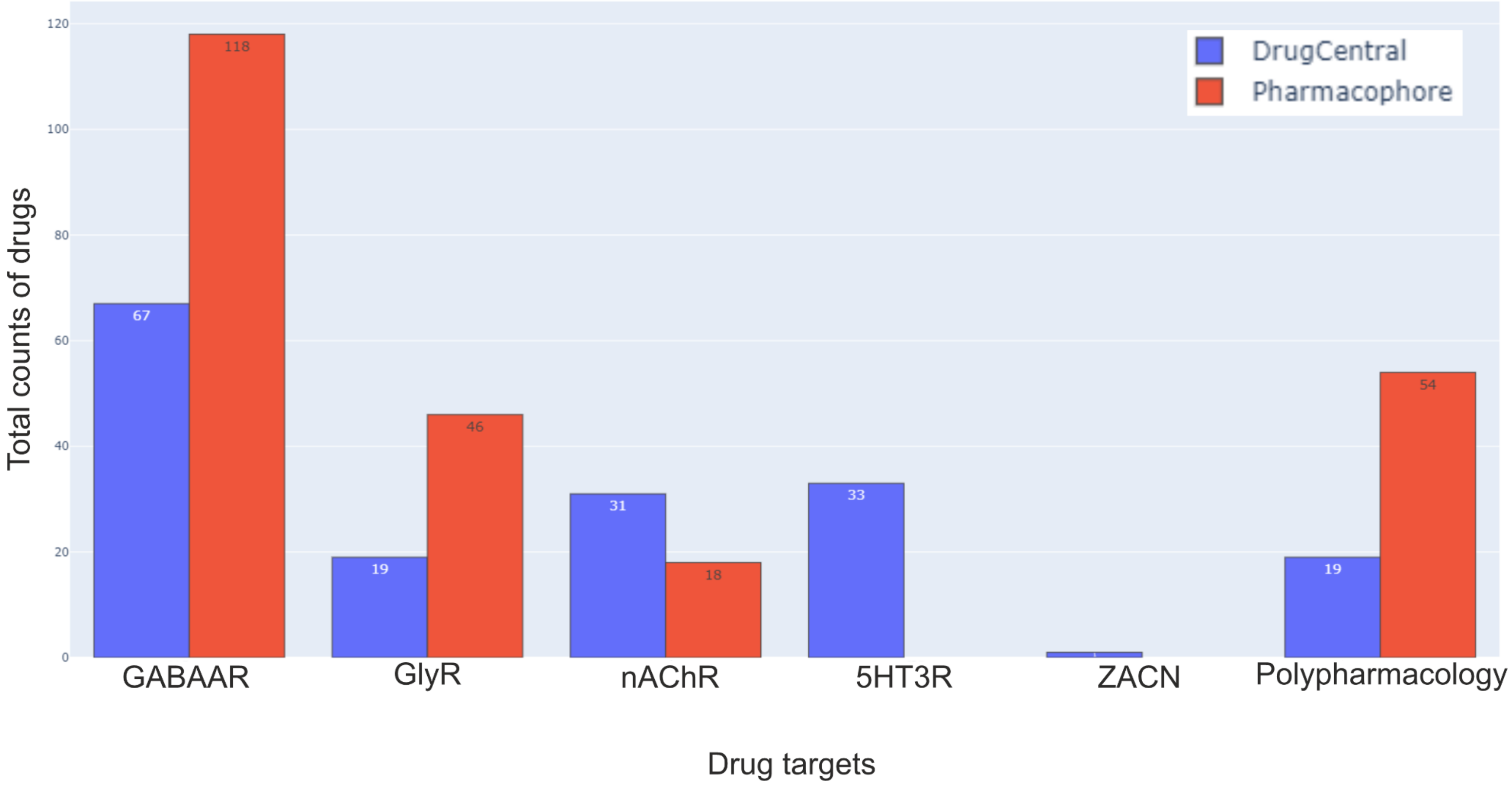

Supplementary Figure 13. Total counts of drugs (y-axis) associated with the target families as reflected by DrugCentral only (blue), and with the addition of our pharmacophore screening results (red). The pharmacophore screening reflects only those binding sites for which structural data was available and for which screens were performed with the exception of the screen into the ivermectin site (Supplementary Figure 11).

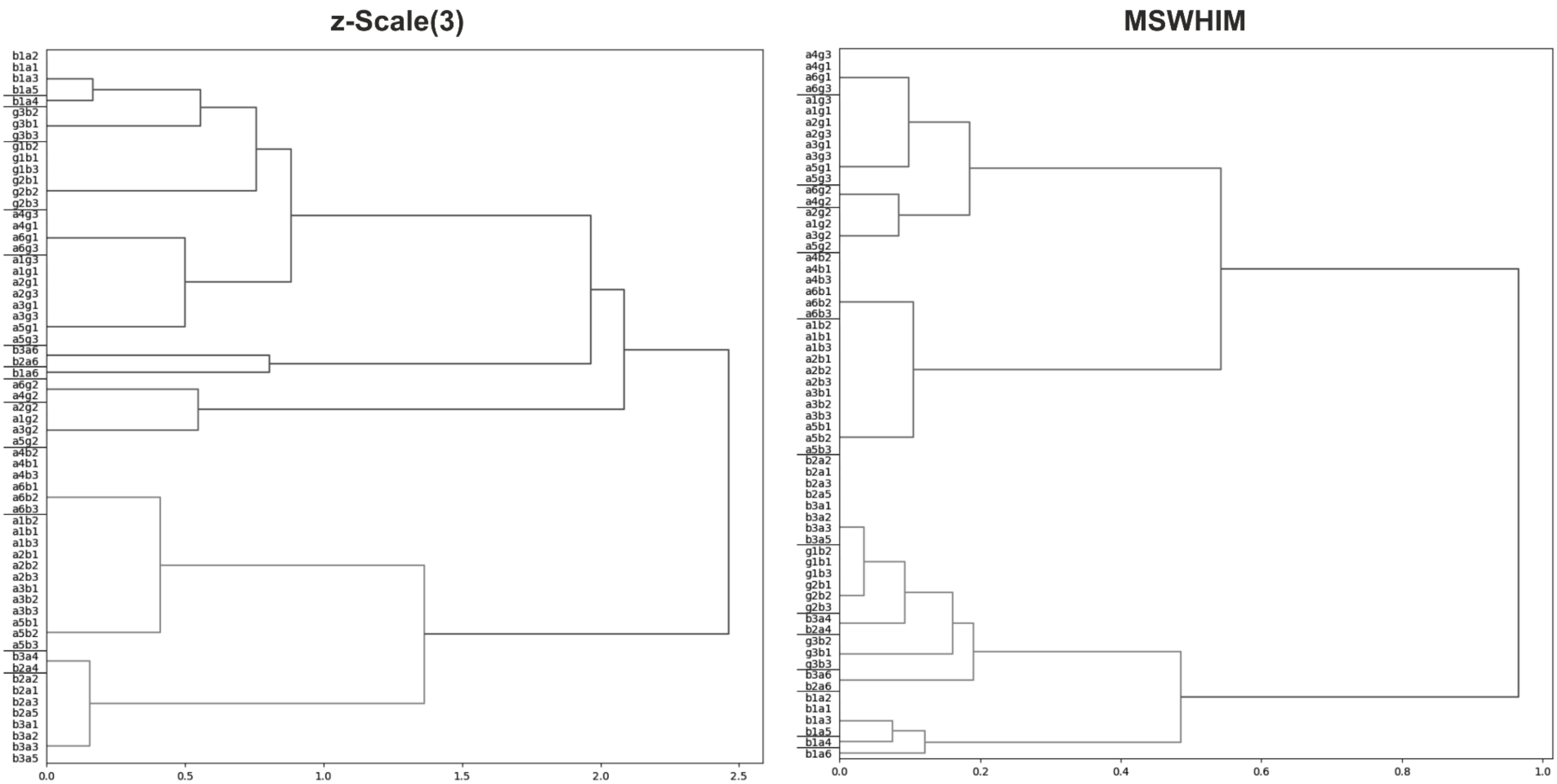

Supplementary Figure 14a. Descriptor set results of the hierarchical clustering for the z-Scale(3) and MSWHIM for the $GABA_A$Rs full pockets of site 3.

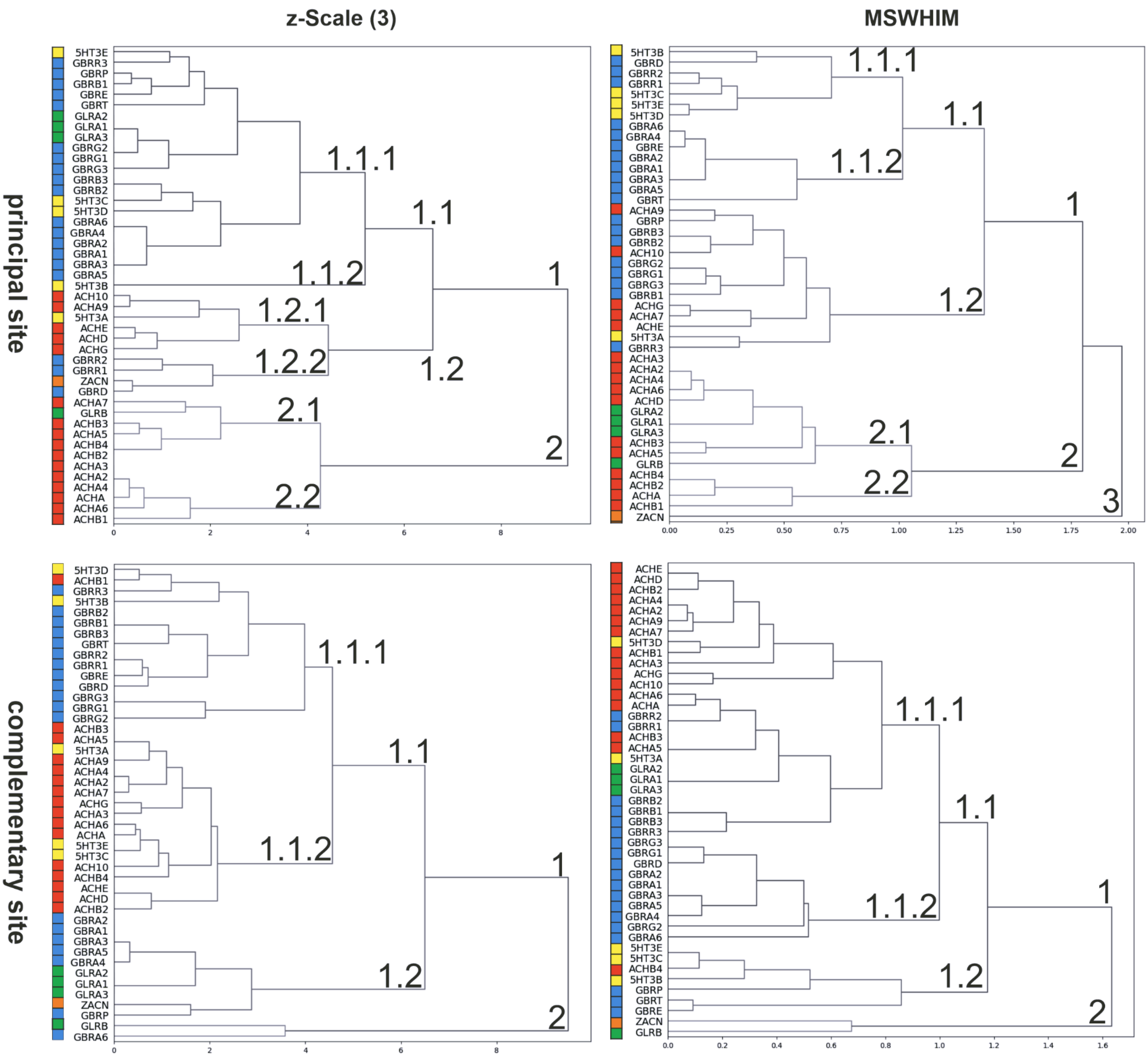

Supplementary Figure 14b. Descriptor set results of the hierarchical clustering for the z-Scale(3) and MSWHIM for the site 3. The numbers on the branching points refer to the comparison diagrams in Figure 4.

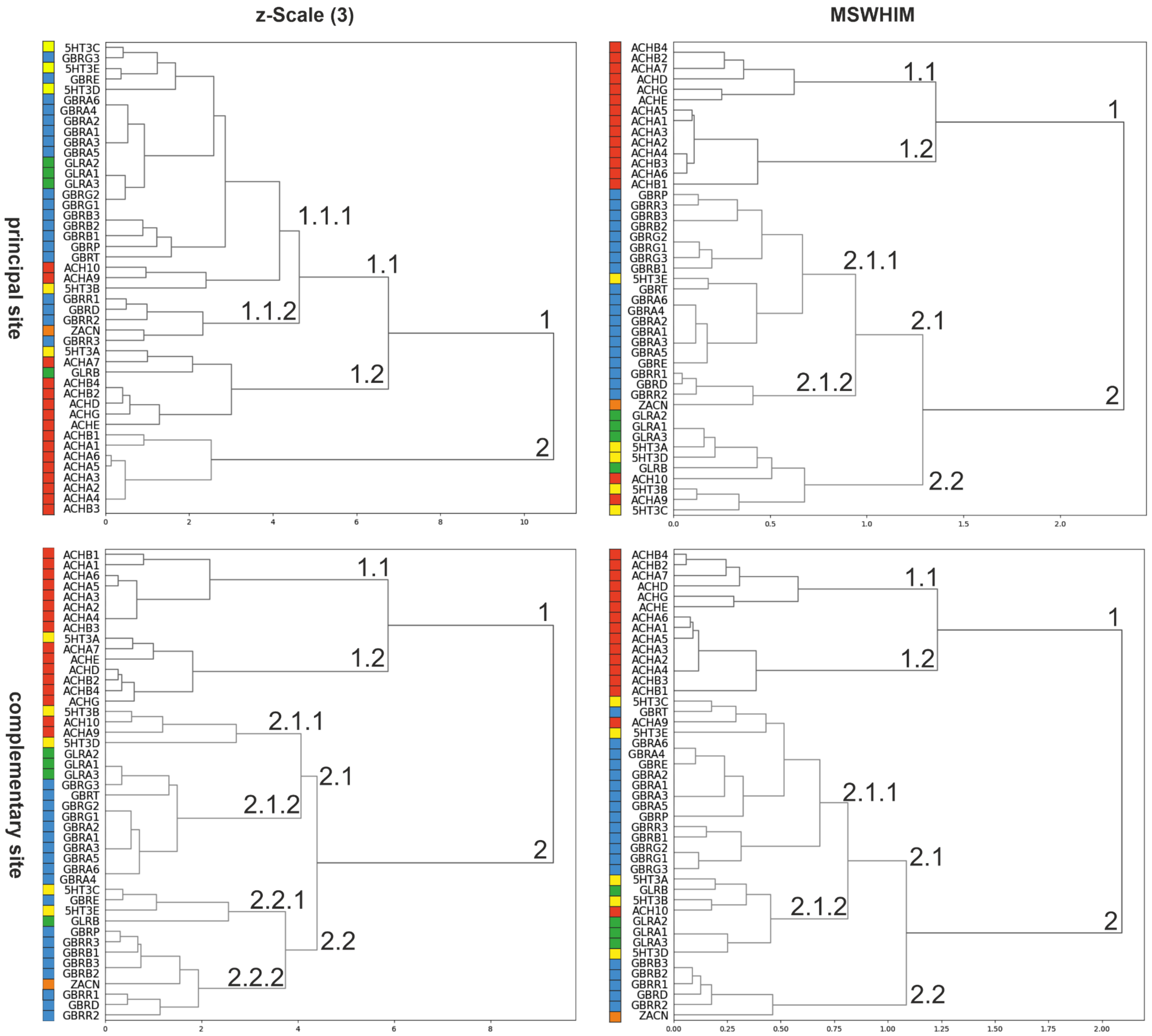

Supplementary Figure 14c. Descriptor set results of the hierarchical clustering for the z-Scale(3) and MSWHIM for the ivermectin binding site. The numbers on the branching points refer to the comparison diagrams in Supplementary Figure 14d.

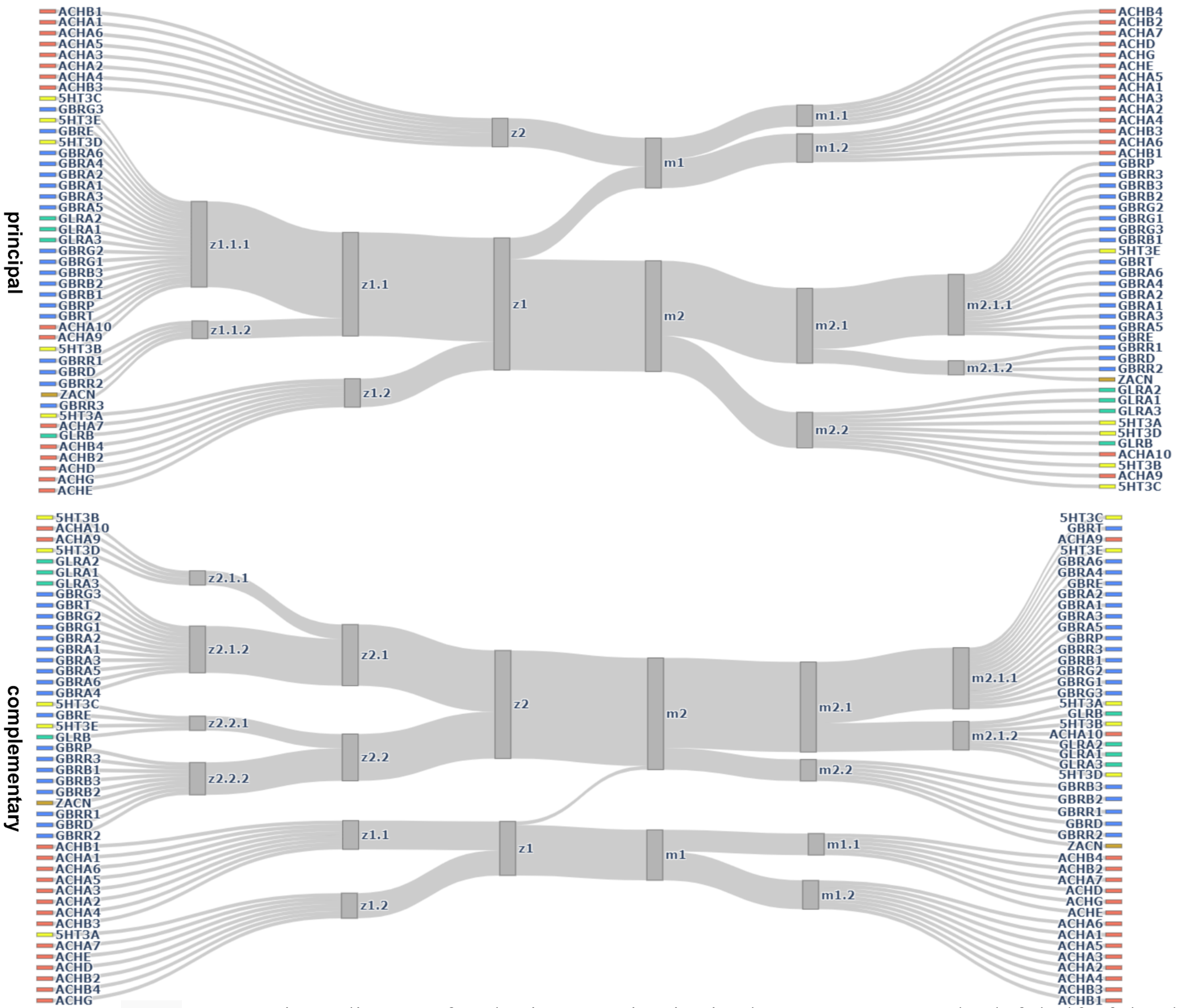

Supplementary Figure 14d. Comparison diagram for the ivermectin site in the upper TMD. The left half of the diagram reflects the z- Scale (3) results, and the right half the MSWHIM results.

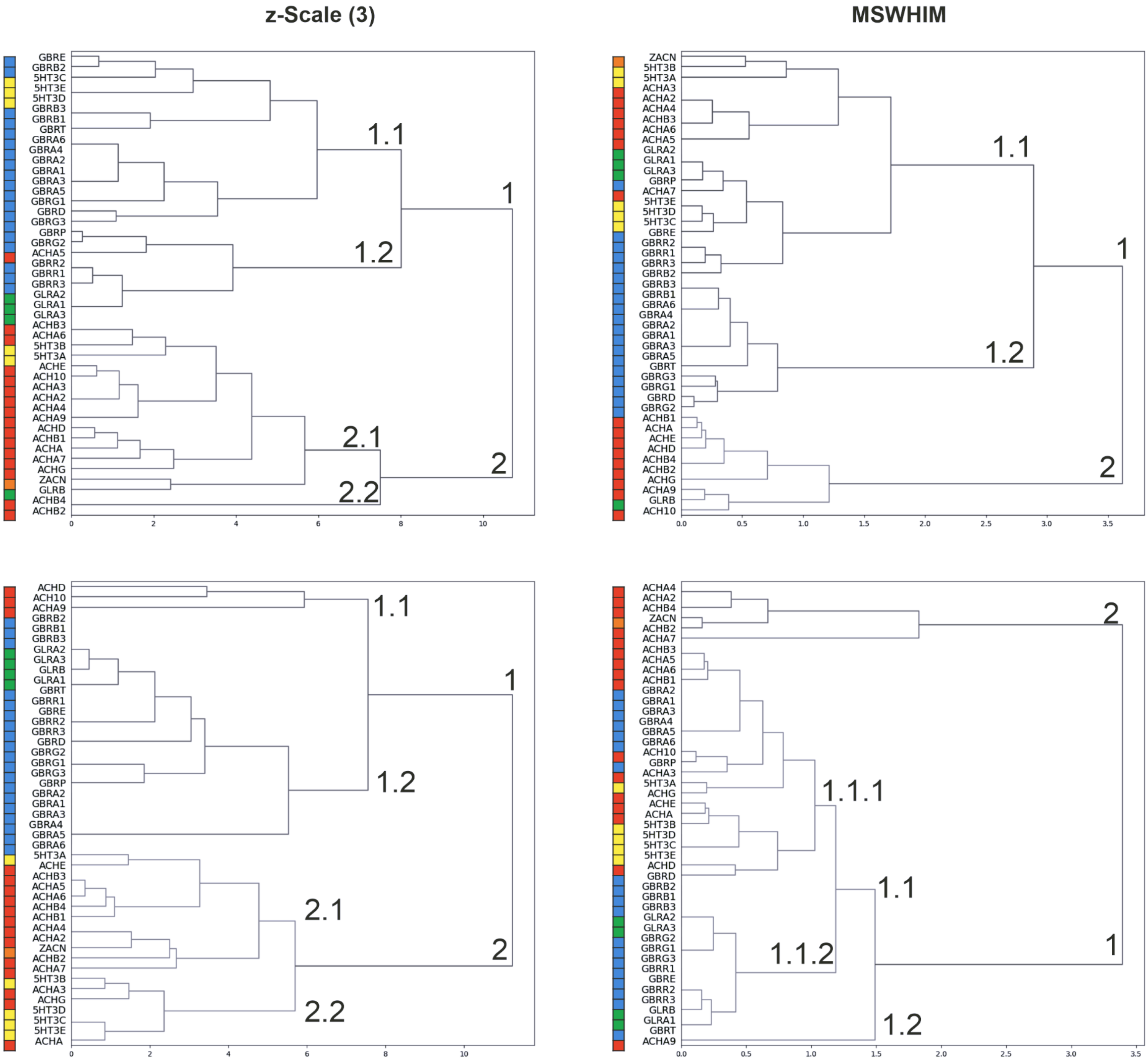

Supplementary Figure 15. Descriptor set results of the hierarchical clustering for the z-Scale(3) and MSWHIM for the site 4. The numbers on the branching points refer to the comparison diagrams in Figure 6.

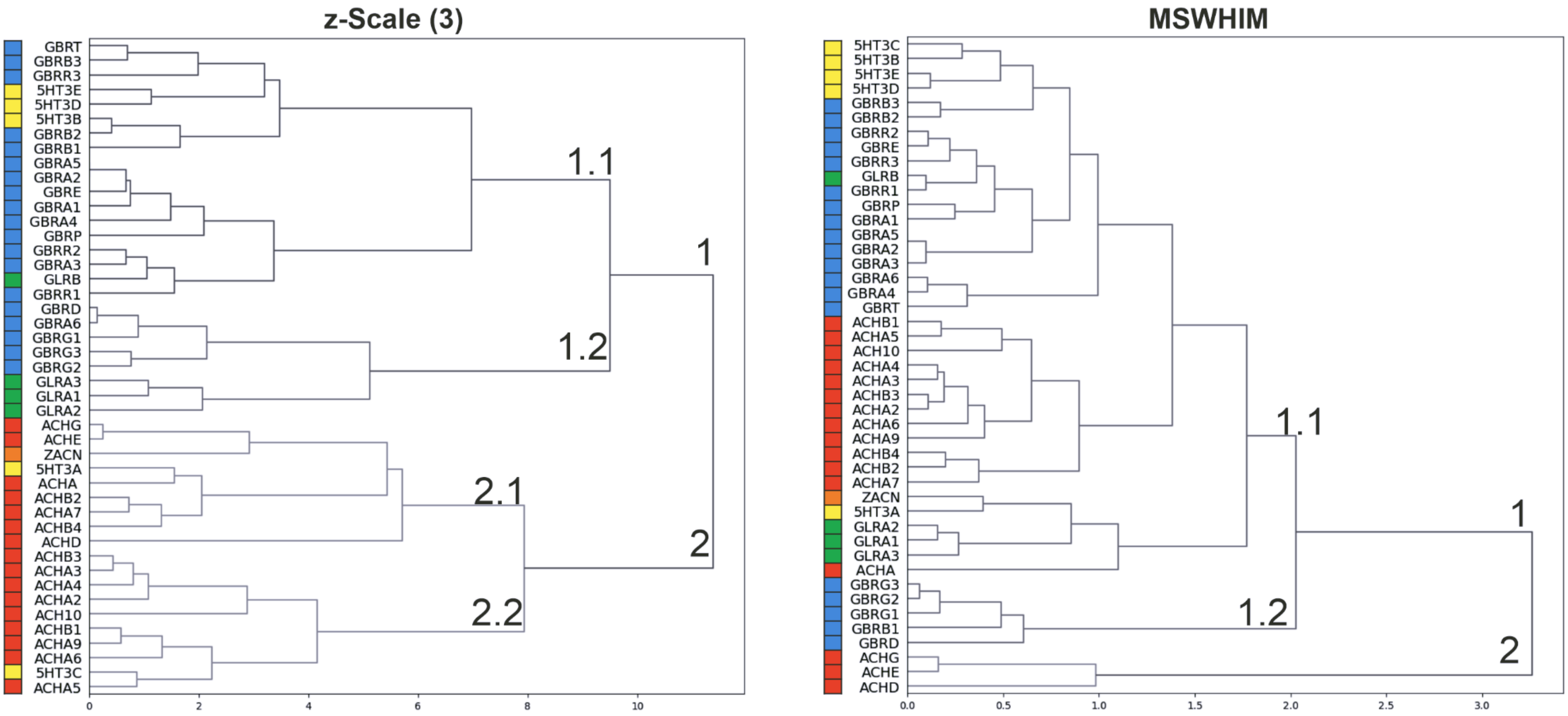

Supplementary Figure 16. Descriptor set results of the hierarchical clustering for the z-Scale(3) and MSWHIM for the site 5. The numbers on the branching points refer to the comparison diagrams in Figure 7.

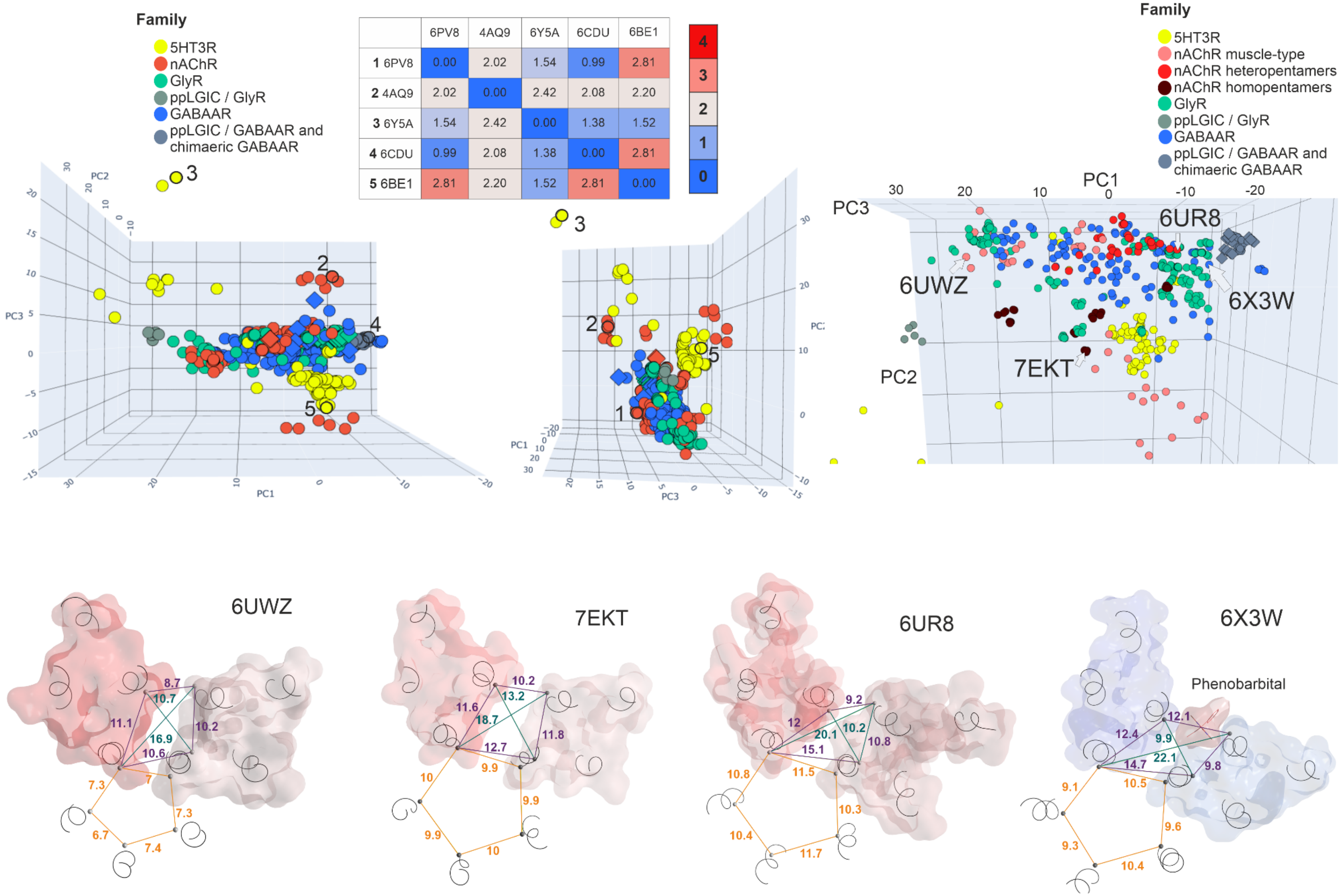

	6PV8	4AQ9	6Y5A	6CDU	6BE1
1 6PV8	0.00	2.02	1.54	0.99	2.81
2 4AQ9	2.02	0.00	2.42	2.08	2.20
3 6Y5A	1.54	2.42	0.00	1.38	1.52
4 6CDU	0.99	2.08	1.38	0.00	2.81
5 6BE1	2.81	2.20	1.52	2.81	0.00

Supplementary Figure 17a: Multiple views of the 3d scatter plot from figure 5 with some conformational differences of three different receptor subtypes from the nicotinic acetylcholine receptors (red structures) and a phenobarbital bound site 3 of a $GABA_A$ receptor (blue). The numbers in the scatter plots identify the structures used for the RMSD matrix, reflecting representative conformations. Specific examples are displayed in the same style as in Figure 5: Different conformations result in different distances between alpha carbon atoms: A plane through the upper TMD is depicted with all five M2 segments and two subunits. Distances between alpha carbons are shown to illustrate backbone conformation, where yellow lines are distances between homologous pore forming M2 residues as an indication for asymmetry, purple lines are distances between pocket forming residues defining the circumference, and green lines the pocket "diagonals".

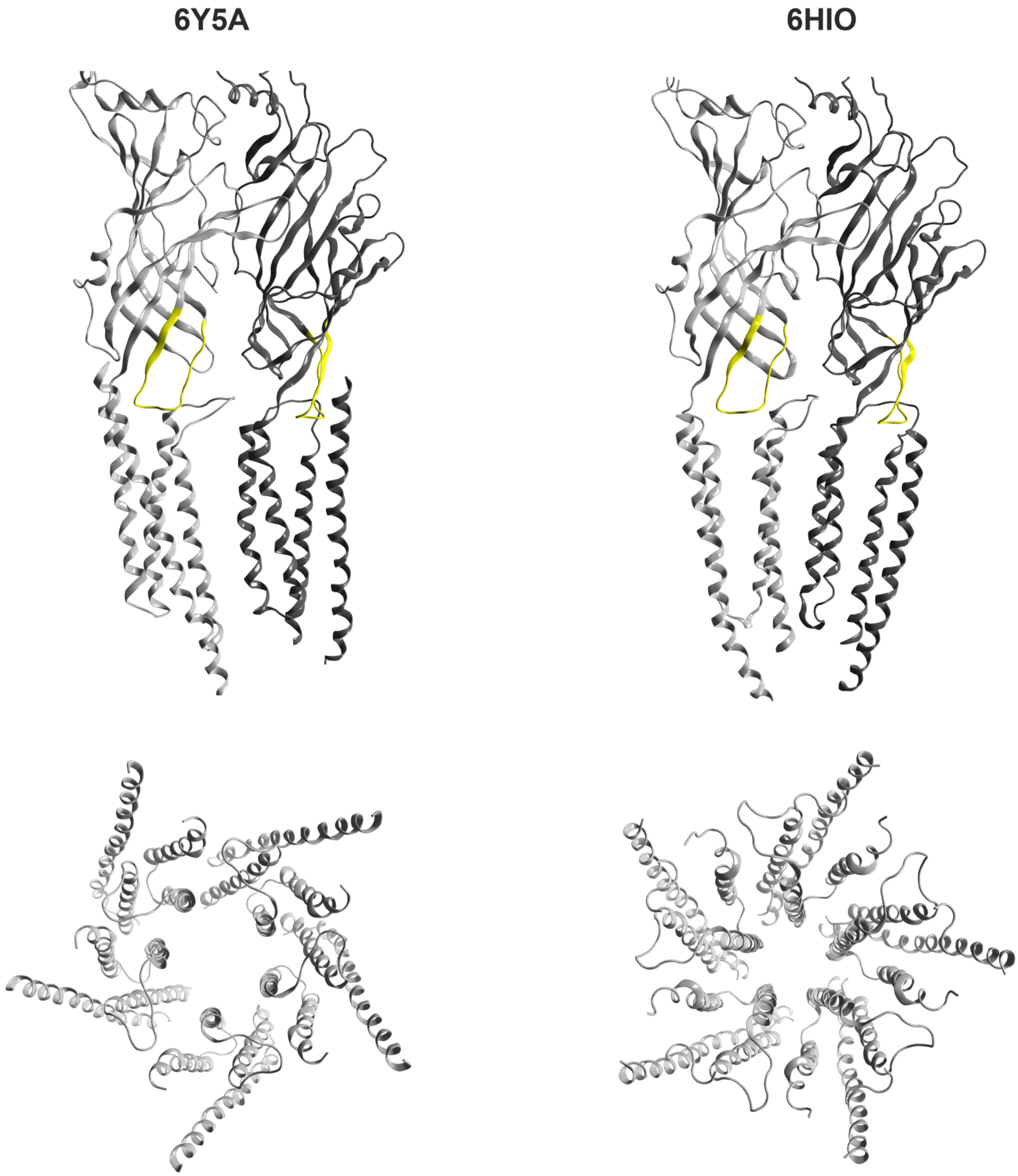

Supplementary Figure 17b: Example of two different conformations of 5HT3A.

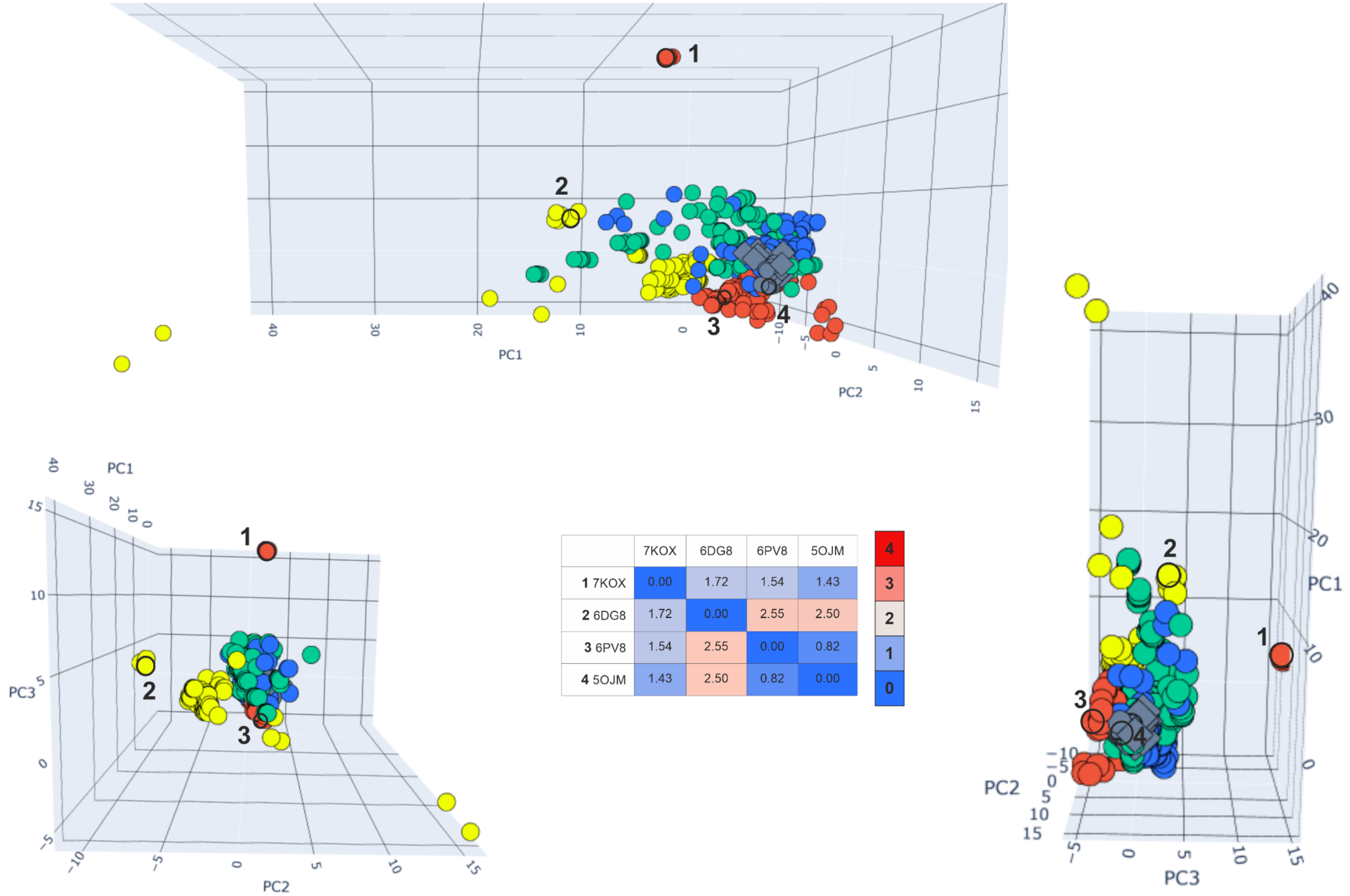

	7KOX	6DG8	6PV8	5OJM
1 7KOX	0.00	1.72	1.54	1.43
2 6DG8	1.72	0.00	2.55	2.50
3 6PV8	1.54	2.55	0.00	0.82
4 5OJM	1.43	2.50	0.82	0.00

Supplementary Figure 17c: Scatter plots of the site 4 conformation analysis matching Figure 8, with representative RMSD values from the labeled structures

frontiers

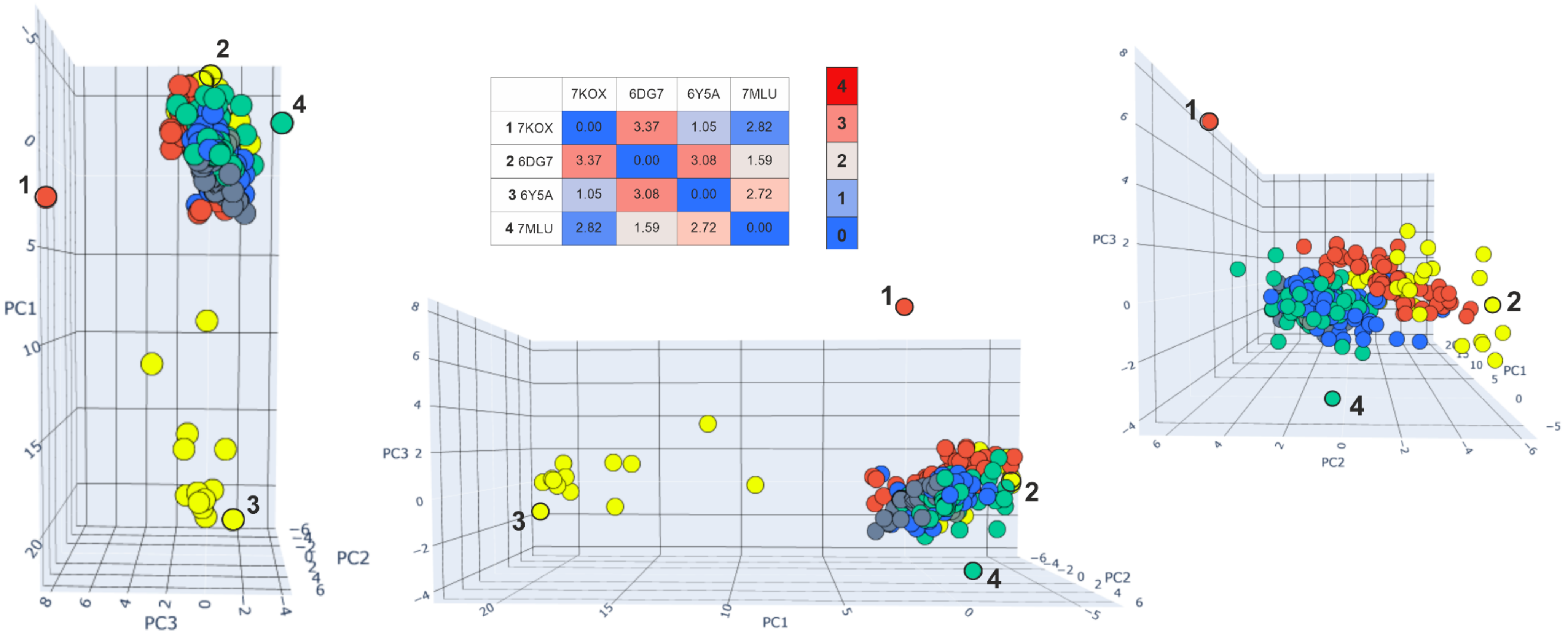

	7KOX	6DG7	6Y5A	7MLU
**1** 7KOX	0.00	3.37	1.05	2.82
**2** 6DG7	3.37	0.00	3.08	1.59
**3** 6Y5A	1.05	3.08	0.00	2.72
**4** 7MLU	2.82	1.59	2.72	0.00

Supplementary Figure 17d: Scatter plots of the site 5 conformation analysis matching Figure 8, with representative RMSD values from the labeled structures

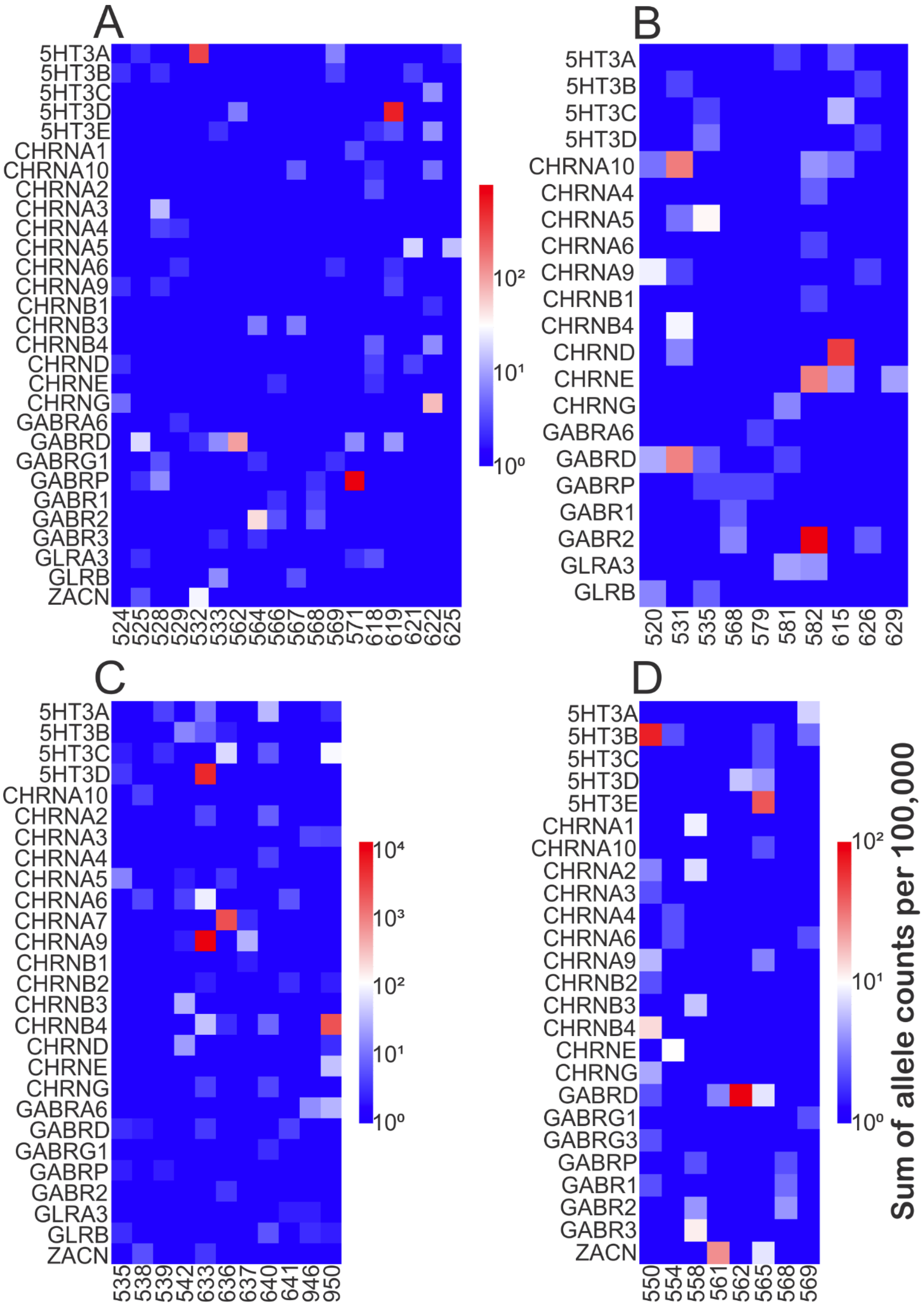

Supplementary Figure 18. Minor allele frequency of variants in TMD subregions. A. Heatmap representing the upper TMD binding site. B. Heatmap representing the extension of the upper TMD binding site with amino acids involved in binding of ivermectin. C. Heatmap representing the steroid binding site. D. Heatmap representing amino acids on M2 helix oriented with side

chains towards the pore. Heatmaps show the sum of minor allele counts per 100,000 in gene and amino acid combinations. Amino acids are numbered according to the reference alignment (Supplementary Item 3). The amino acids correspond to those which are color coded in Supplementary Figure 18 for the upper and lower TMD binding sites, the PS-site.

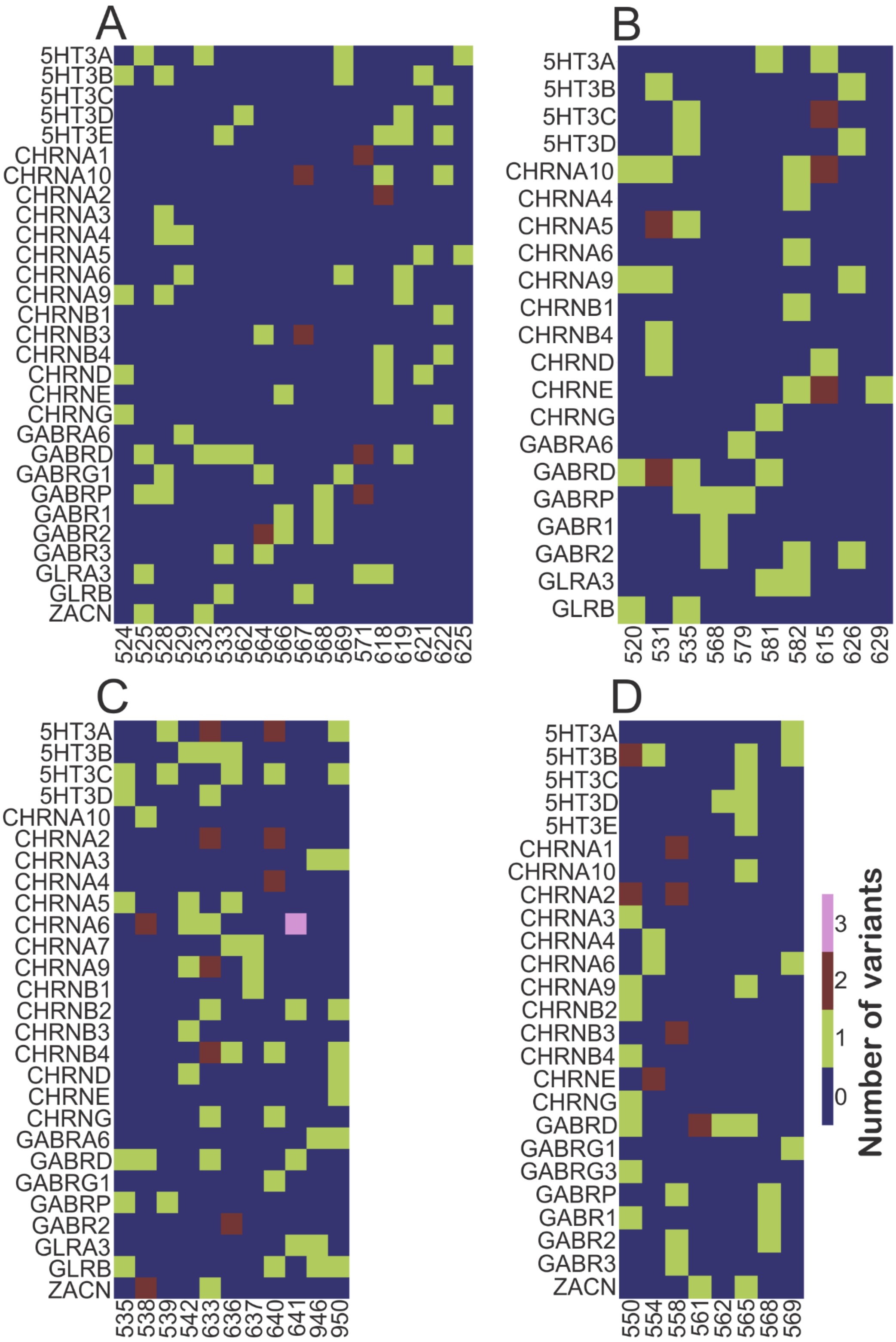

Supplementary Figure 19. Number of different variants in TMD subregions. A. Heatmap representing the upper TMD binding site. B. Heatmap representing the extension of the upper

TMD binding site with amino acids involved in binding of ivermectin. C. Heatmap representing the steroid binding site. D. Heatmap representing amino acids on M2 helix oriented with side chains towards the pore. Amino acids are numbered according to the reference alignment.

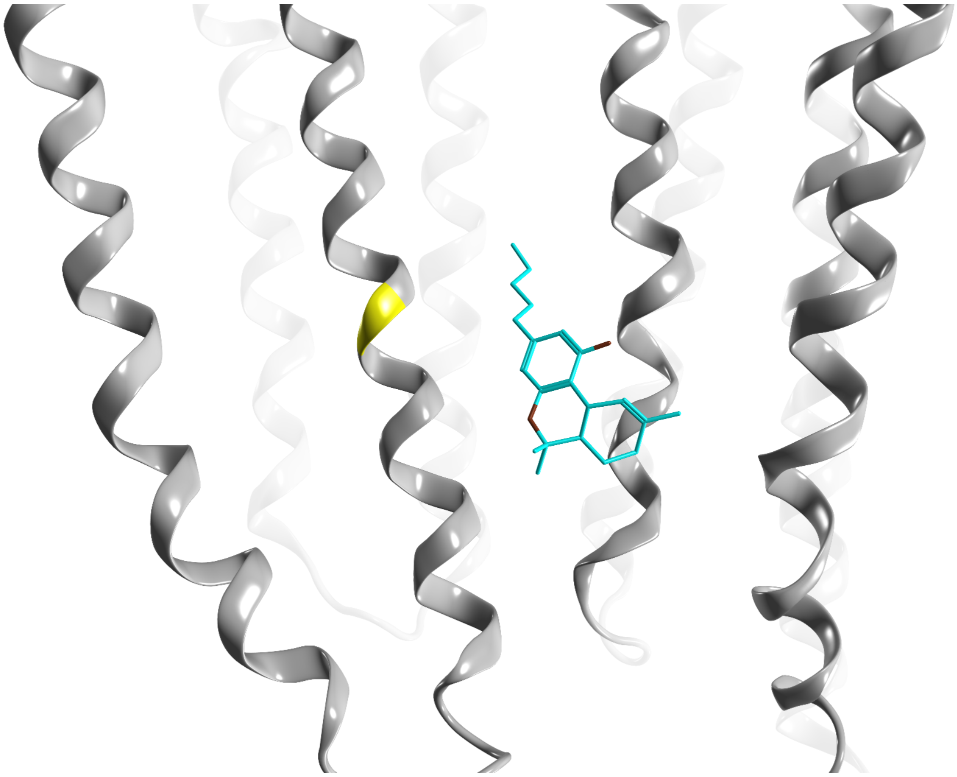

Supplementary Figure 20. An alternative binding mode for the docking of THC into 5OSC showing close proximity to the glycine α3.

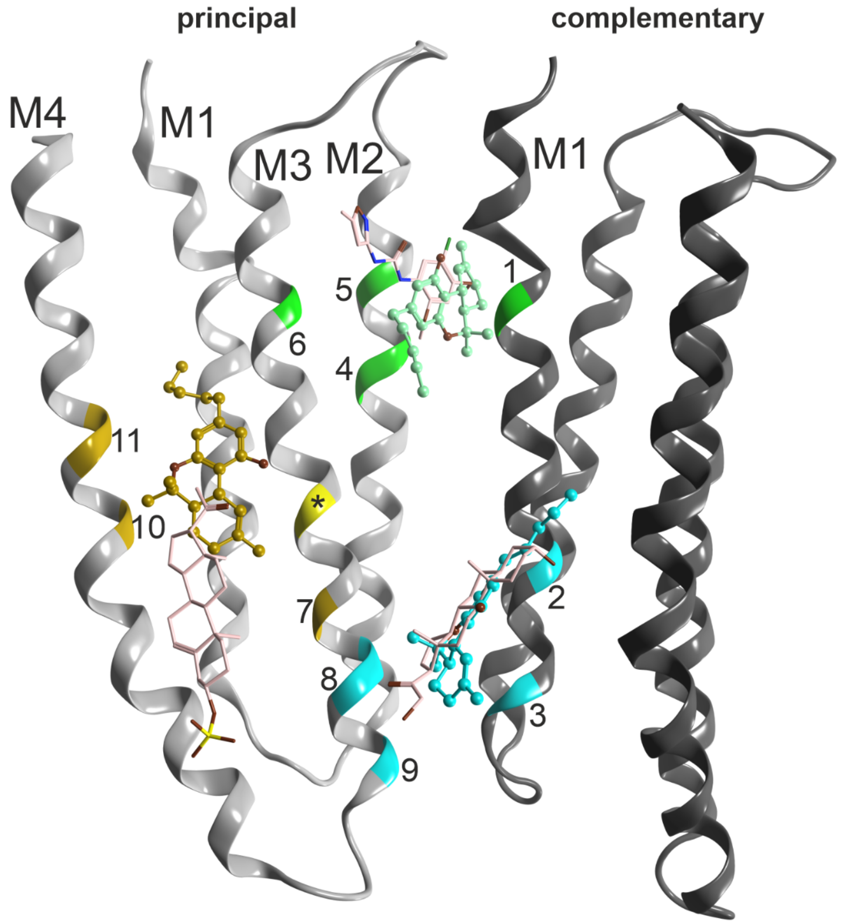

	1	2	3	4	5	6
	231	241	245	265 266	269	290
GABRA1	L	Q	W	MT	S	A
GABRA6	T	Q	W	MT	S	A
GABRB2	M	W	W	MT	N	M
GLRA3	I	W	W	MT	S	A

	7	8	9	10	11
	302	304 305	308	399	402 403
GABRA1	E	AT	Y	F	LF
GABRA6	E	AA	Y	F	AF
GABRB2	E	AL	Y	F	VF
GLRA3	E	AA	F	F	AF

Supplementary Figure 21. Tentative THC binding sites and candidate positions for mutational analysis. Ribbon rendering of two subunits' TMD domains, helices that contribute to sites 3, 4 or 5 are labeled. Colour codes match Figure 9. Site 3 in the upper TMD: Representative docking pose of THC (green) in superposition with PNU-120596 (light pink, 7EKT). Green ribbon segments on principal M2, M3 and on complementary M1 form ligand contacts. The segment numbering matches the table below the image, amino acid numbering as

in (PDBID - 5OSB) for all sites. Site 4: Representative docking pose of THC (cyan) in superposition with THDOC (light pink, 5OSB). Cyan ribbon segments on principal M3 and complementary M4 form ligand contacts. Site 5: Representative docking pose of THC (gold) in (partial) superposition with preganolone sulfate (PS, light pink, 5OSC). The best scoring docking poses overlap only to a small extent with PS. Golden ribbon segments on M3 and M4 form ligand contacts. The bright yellow segment with the * marks the position which has been mutated in the GlyR α3 subunit, see Figure 9.

2.2 Supplementary Tables

Supplementary Table 1: Gene names and their corresponding uniprot IDs.

Gene name	Uniprot ID
GABRA1	P14867
GABRA2	P47869
GABRA3	P34903
GABRA4	P48169
GABRA5	P31644
GABRA6	Q16445
GABRB1	P18505
GABRB2	P47870
GABRB3	P28472
GABRG1	Q8N1C3
GABRG2	P18507
GABRG3	Q99928
GABRD	O14764
GABRE	P78334
GABRQ	Q9UN88

GABRR1	P24046
GABRR2	P28476
GABRR3	A8MPY1
GABRP	O00591
GLRA1	P23415
GLRA2	P23416
GLRA3	O75311
GLRB	P48167
ACHA1	P02708-2
ACHA2	Q15822
ACHA3	P32297
ACHA4	P43681
ACHA5	P30532
ACHA6	Q15825
ACHA7	P36544
ACHA9	Q9UGM1
ACHA10	Q9GZZ6
ACHB1	P11230
ACHB2	P17787
ACHB3	Q05901
ACHB4	P30926
ACHE	Q04844
ACHD	Q07001
ACHG	P07510
HTR3A	P46098

HTR3B	O95264
HTR3C	Q8WXA8
HTR3D	Q70Z44
HTR3E	A5X5Y0
ZACN	Q401N2

Supplementary Table 2: Structures used for the pharmacophore screens.

**Pocket location**	**Binding site forming subunits**	**PDB files used for merged Ph4**	**Ligands bound to template structure**
novel upper ECD interface - **2**	Gly (a3+/a3-)	5TIN; 5TIO	AM-3607
upper TMD interface - **3**	GABA_(b(2/3)+/a1-)	6HUP, 6X3T, 6X3V	Propofol, Diazepam, Etomidate
	GABA (g2+/b2-)	6X3X; 6X3W	Diazepam, Phenobarbital
	GABA (a1+/b2-); (g2+/b2-)	6X3W	Phenobarbital
	Ach (a7+/a7-)	7EKT	PNU-120596
	Gly (a3+/a3-)	5VDH	Ivermectin

Lower TMD interface - **4**	GABA (a1+/a1-)	5OSB; 5O8F; 6CDU	Alfaxalone, THDOC, Pregnanolone
Lipid M3/M4 associated site - **5**	GABA (a1)	5OSC	Pregnanolone sulfate

Pharmacophore-based virtual screening:

Pharmacophores (Ph4s) are ensembles of ligand features which are deemed essential for an optimal drug-target interaction in order to trigger a biological response (https://doi.org/10.1351/pac199870051129). 3D structures of protein bound ligands can be utilized to derive features abstracting observed non-bonding interactions as well as features describing the binding site structure (exclusion volumes). An in-silico screening of large compound databases using such structure-based PH4s as filters allows to quickly identify smaller subsets of compounds likely to be active and thus to prioritize for time and cost intensive in-vitro assays. Inherent limitations of the method come from several sources, where the general availability of target-bound ligand 3D structures and the resolution and quality of the experimental structure are the most important ones. When multiple 3D structures of different ligands bound to identical or highly homologous target sites exist, shared-feature pharmacophores can be constructed. This modeling technique allows to identify features that are not essential for ligand binding and might represent just ligand and/ -or binding site-specific artifacts present in the experimental structures. Likewise, merged-feature pharmacophores can be generated which offer a combined description of multiple observed ligand binding modes and thus provide a more comprehensive coverage of the chemical space of potential binders to the investigated target site. In this study, hit lists obtained for the structure-based pharmacophores of a ligand bound to multiple equivalent sites of the same target were generally merged together for the sake of simplicity and to average out slight site-specific pharmacophore differences (feature positions and additional observed interactions). Hit lists that were obtained for the generated merged pharmacophore models get presented as they were retrieved from the performed screening runs.
